# Effect of Clinical Decision Support at Community Health Centers on the Risk of Cardiovascular Disease: A Cluster Randomized Clinical Trial

**DOI:** 10.1001/jamanetworkopen.2021.46519

**Published:** 2022-02-04

**Authors:** Rachel Gold, Annie E. Larson, JoAnn M. Sperl-Hillen, David Boston, Christina R. Sheppler, John Heintzman, Carmit McMullen, Mary Middendorf, Deepika Appana, Vijayakumar Thirumalai, Ann Romer, Julianne Bava, James V. Davis, Nadia Yosuf, Jenny Hauschildt, Kristin Scott, Susan Moore, Patrick J. O’Connor

**Affiliations:** 1 Center for Health Research, Kaiser Permanente Northwest, Portland, Oregon; 2 OCHIN Inc, Portland, Oregon; 3 HealthPartners Institute, Minneapolis, Minnesota

## Abstract

**Importance:**

Management of cardiovascular disease (CVD) risk in socioeconomically vulnerable patients is suboptimal; better risk factor control could improve CVD outcomes.

**Objective:**

To evaluate the impact of a clinical decision support system (CDSS) targeting CVD risk in community health centers (CHCs).

**Design, Setting, and Participants:**

This cluster randomized clinical trial included 70 CHC clinics randomized to an intervention group (42 clinics; 8 organizations) or a control group that received no intervention (28 clinics; 7 organizations) from September 20, 2018, to March 15, 2020. Randomization was by CHC organization accounting for organization size. Patients aged 40 to 75 years with (1) diabetes or atherosclerotic CVD and at least 1 uncontrolled major risk factor for CVD or (2) total reversible CVD risk of at least 10% were the population targeted by the CDSS intervention.

**Interventions:**

A point-of-care CDSS displaying real-time CVD risk factor control data and personalized, prioritized evidence-based care recommendations.

**Main Outcomes and Measures:**

One-year change in total CVD risk and reversible CVD risk (ie, the reduction in 10-year CVD risk that was considered achievable if 6 key risk factors reached evidence-based levels of control).

**Results:**

Among the 18 578 eligible patients (9490 [51.1%] women; mean [SD] age, 58.7 [8.8] years), patients seen in control clinics (n = 7419) had higher mean (SD) baseline CVD risk (16.6% [12.8%]) than patients seen in intervention clinics (n = 11 159) (15.6% [12.3%]; *P* < .001); baseline reversible CVD risk was similarly higher among patients seen in control clinics. The CDSS was used at 19.8% of 91 988 eligible intervention clinic encounters. No population-level reduction in CVD risk was seen in patients in control or intervention clinics; mean reversible risk improved significantly more among patients in control (−0.1% [95% CI, −0.3% to −0.02%]) than intervention clinics (0.4% [95% CI, 0.3% to 0.5%]; *P* < .001). However, when the CDSS was used, both risk measures decreased more among patients with high baseline risk in intervention than control clinics; notably, mean reversible risk decreased by an absolute 4.4% (95% CI, −5.2% to −3.7%) among patients in intervention clinics compared with 2.7% (95% CI, −3.4% to −1.9%) among patients in control clinics (*P* = .001).

**Conclusions and Relevance:**

The CDSS had low use rates and failed to improve CVD risk in the overall population but appeared to have a benefit on CVD risk when it was consistently used for patients with high baseline risk treated in CHCs. Despite some limitations, these results provide preliminary evidence that this technology has the potential to improve clinical care in socioeconomically vulnerable patients with high CVD risk.

**Trial Registration:**

ClinicalTrials.gov Identifier: NCT03001713

## Key Points

**Question:**

Does a clinical decision support system (CDSS) proven to reduce cardiovascular risk in integrated care settings also reduce cardiovascular risk in community health centers?

**Findings:**

In this cluster randomized clinical trial of 18 578 eligible patients, although CDSS adoption rates were low, CDSS use was associated with significantly improved reversible risk of cardiovascular disease among patients with the highest levels of baseline risk.

**Meaning:**

The use of a CDSS in community health centers has the potential to improve reversible risk of cardiovascular disease among socioeconomically vulnerable high-risk patients; strategies to increase CDSS adoption in this setting are needed.

## Teaser

This cluster randomized clinical trial assesses whether a clinical decision support system developed in an integrated care setting improves outcomes of patients at risk for cardiovascular disease who are treated in community health centers.

## Introduction

Evidence-based management of modifiable risk factors for cardiovascular disease (CVD) can substantially reduce CVD-related morbidity and mortality risks. However, a deficit persists between recommended and observed CVD risk management, especially among socioeconomically vulnerable patients.^1,2,3,4,5,6^ One reason for this is that primary care clinicians must consider multiple factors affecting CVD risk for a given patient^7^ and determine which to address to optimally affect that patient’s risk within a brief encounter.^8,9,10,11,12,13,14,15^

Electronic health record (EHR)–based clinical decision support systems (CDSS) address such barriers by alerting clinicians when patients have uncontrolled CVD risks and suggesting treatment options.^16,17,18,19,20,21,22,23,24,25,26,27,28,29,30,31^ CV Wizard, for example, is a nonproprietary, web-based CDSS developed at HealthPartners Institute, a large, nonprofit, integrated health care system.^32,33,34,35,36^ The CV Wizard algorithms reflect current CVD care guidelines^37,38,39,40,41,42,43^ and account for a patient’s blood pressure (BP), laboratory results, distance from goals, medications, and comorbidities. At HealthPartners Institute and in similar settings, rates of use and reported user satisfaction were high, and use was associated with significant decreases in CVD risk measures.^32,33,34,35,36^

Little evidence exists on the effects of CDSS in underresourced settings^23,24,25,26,27,28,29,30,31,33,44,45,46,47^ such as community health centers (CHCs), which serve more than 38 million socioeconomically vulnerable US residents annually. Implementing a CDSS that has proven effective in other settings could enhance CVD risk management in CHCs. Evidence is needed about the effect of CDSS such as CV Wizard in CHCs, whose patients have high rates of uncontrolled CVD risk and medical and social complexity.^2,3,4,48,49^ This cluster randomized clinical trial is one of the first to assess whether a CDSS developed in an integrated care setting improves outcomes in CHCs.

## Methods

### Setting

OCHIN Inc is a national nonprofit operating the largest network of community-based care organizations in the country. Its members (96 CHC organizations running 493 clinic sites in 14 states as of September 2018) share an OCHIN Epic EHR. In 2017 to 2018, CV Wizard was set up to work in this EHR^50^ and pilot-tested in 2 OCHIN Inc member organizations (9 clinics).

### Ethics Approval and Safety Monitoring

The Kaiser Permanente Northwest institutional review board approved all research activities and monitored study progress. A data and safety monitoring board monitored safety outcomes. The institutional review board granted a waiver for obtaining patient consent in this cluster randomized clinical trial, and all OCHIN Inc members sign an agreement that their EHR data may be used for research. The original and current institutional review board–approved study protocols are available in Supplement 1. This study followed the Consolidated Standards of Reporting Trials (CONSORT) reporting guideline.

### Sample Size, Recruitment, and Randomization

Our power calculations conservatively estimated needing 30 clinics per group. They then varied effect size associated with group × time interaction and intraclass correlation to assess power to identify a 1.5%, 2.0%, or 3.0% absolute reduction in risk score over time in intervention vs control sites.

In Spring 2018, 70 OCHIN Inc member clinics run by 15 CHC organizations were recruited (10 more than needed, in case of dropout). Eligible clinics served at least 35 adults with hypertension annually and were not the pilot sites. They were cluster randomized 1:1 to the intervention or the control group by organization (to avoid contamination between clinics in the same organization) as follows. Random numbers were generated for each organization, which were then assigned to groups above or below the random numbers’ median. This process was repeated 5 times. The grouping with the most even distribution of organization size (number of encounters in the prior year) and patient characteristics (percentage of tobacco users and percentage with hypertension) was used to assign intervention and control status.

### Study Design

CV Wizard was activated in the intervention and control organizations in September 2018. It ran invisibly in the control clinics to collect data without giving access to the tool. Patients seen at study clinics in the first 6 months after tool activation were followed up for at least 1 year during an 18-month comparison period (September 20, 2018, to March 15, 2020). Control sites received the CDSS after follow-up.

### Intervention

At the point of care, CV Wizard identified patients aged 40 to 75 years with diabetes or atherosclerotic CVD and at least 1 uncontrolled CVD risk factor, or high reversible risk (>10% ten-year risk of a cardiovascular event), calculated based on other CVD risk factors. Reversible risk was calculated as the difference between the patient’s current state and the expected risk should the patient achieve control goals recommended by national guidelines (details are provided hereinafter).^43^ When a patient met CDSS eligibility criteria, rooming staff saw an EHR alert containing a link to the CV Wizard interfaces. The recommended workflow was to then view (1 click) and print (1 click) the interfaces, which show the patient’s 10-year CVD risk and how they could lower it by following personalized care recommendations. Users could access a clinician version (eFigure 1 in Supplement 2) and a patient version (eFigure 2 in Supplement 2), the latter in either English or Spanish. Tool use was defined as the proportion of primary care encounters among eligible patients where the CDSS tool’s output was viewed and/or printed. Tool use counts included use at the index visit or subsequent study period visits, excluding the patient’s last visit during the study period.

### Data Source

Data on 10-year CVD risk and reversible risk were collected through CV Wizard’s web service for all eligible patients. Additional EHR-extracted data came from the Accelerating Data Value Across a National Community Health Center Network (ADVANCE) Clinical Research Network, a Patient-Centered Clinical Research Network member, including demographic characteristics, BP, medications, laboratory values, diagnostic codes, and clinic characteristics. Outcomes were assessed using encounter data from all postindex visits.

### Study Participants

The target population consisted of patients meeting the aforementioned CV Wizard eligibility criteria excluding those with current or recent pregnancy, active cancer, or hospice or palliative care. Analyses included target patients with an index visit at a study clinic in the 6 months after CV Wizard activation and 1 or more postindex encounters during 12 months of follow-up. This ensured adequate follow-up data for the study analyses.

Patients newly diagnosed with diabetes during the follow-up period (347 [1.9%]) were excluded from CVD risk analyses because diabetes may be diagnosed more often with CDSS use. Because the presence of diabetes substantially raises total CVD risk estimation, this exclusion removed the possibility of confounding based on the likelihood of a new diabetes diagnosis.

### Outcomes

The primary outcomes were 1-year change in total CVD risk and 1-year change in reversible CVD risk. Total CVD risk was estimated using the American College of Cardiology/American Heart Association pooled risk equations, which include age, race and ethnicity, sex, systolic BP, total cholesterol level, high-density lipoprotein cholesterol level, and diabetes, smoking, and antihypertensive medication status.^51^ Race and ethnicity data were extracted from the EHR; these data are collected by the study clinics as part of patient care. These data were considered relevant to the study because rates of uncontrolled CVD risk differ across racial/ethnic groups. Reversible CVD risk was calculated as follows (with additional details in the eMethods in Supplement 2 and methods described previously^32,33,34,35,36^). Standardized equations estimated the potential reduction in CVD risk if a patient’s uncontrolled risk factors reached evidence-based thresholds. Change was calculated by subtracting reversible risk at follow-up from that at index visit; negative values represent favorable changes. This approach focuses clinical attention on patients with high reversible CVD risk, rather than all patients with high total CVD risk.^34^ It also focuses on risk factors not adequately addressed by American College of Cardiology/American Heart Association equations: change in hemoglobin A_1c_ (HbA_1c_) level or body mass index and aspirin use. This estimation of patient-level reversible CVD risk, although imprecise, is likely superior to the demonstrably erroneous estimates of CVD benefits and risks broadly used in primary care and to intuitive estimates of benefit or risk.^32,33,52^ In exploratory analyses, these outcomes were stratified by baseline risk of less than 10%, 10% to less than 20%, and at least 20%, because analyses in other settings indicated CV Wizard’s potentially greater impact among patients with higher baseline risk,^34^ patients with lower baseline risk were less likely to improve, and clinical implications for these groups differ. Secondary outcomes assessed change in BP, low-density lipoprotein (LDL) level, and HbA_1c_ levels for those above goal at baseline; analyses included patients with at least 1 follow-up value.

### Statistical Analysis

Descriptive statistics compared baseline characteristics of intervention vs control groups and CHC organizations. We used a 2-tailed χ^2^ test, unparied *t* test, and nonparametric Wilcoxon rank sum test as appropriate. The threshold for statistical significance was *P* < .05 and was 2-tailed.

The extent of CDSS adoption drives its population-level impact,^53,54^ and therefore analyses differentiated between the tool’s population impact and its impact when used. Intention-to-treat (ITT) analyses (all targeted patients at intervention vs control organizations) assessed population impact. The effect of treatment on the treated (ETOT; also termed *per protocol*) analyses assessed impact when used, comparing patients in the intervention CHC group with matched controls (each group matched separately to control patients) based on exposure to the CDSS.

In 3-level random-intercept models (encounter nested within patient and clinic), the intraclass correlation coefficients at the clinic level were 0.05 for CVD risk, 0.04 for both reversible risk and BP, and less than 0.03 for HbA_1c_ and LDL levels. In 4-level random-intercept models (encounter nested within patient, clinic, and organization), intraclass correlation coefficients at the organization level were at least 0.02 for reversible risk, BP, and HbA_1c_ and LDL levels, and 0.05 for CVD risk. Because of the complexity of fitting the 4-level models, and the low organization-level intraclass correlation coefficient, all models were fit using a 3-level random intercept.

#### ITT Analysis

Differences in outcome changes were assessed using multilevel mixed models adjusted for these individual-level fixed effects: baseline CVD risk; distribution of eligible patients by age, race and ethnicity, sex, rural-urban commuting area status, and federal poverty level at index visit; and number of ambulatory visits during the follow-up period. These variables, selected a priori, were confirmed by descriptive analyses showing significant differences between baseline intervention and control organization and patient characteristics. Residual distributions indicated that linear-mixed models were appropriate for all outcomes except the reversible risk model that included all study patients, which had a negative binomial distribution.

#### ETOT Analysis

To assess the CDSS tool’s impact when it was used, analyses were conducted among patients for whom the tool was ever used during follow-up. To assess association with increasing use, we also considered 3 categories of tool use (never, once, and more than once).

Per protocol analyses require adjusting for loss to follow-up and off-protocol therapies or treatments,^55^ but if group differences vary too greatly, model misspecification can yield biased estimates. Propensity score methods are an alternative.^56^ In ETOT analyses of change in CVD risk, patients in the intervention organizations were matched to those in control organizations based on age, federal poverty level, outcome of interest at baseline, race and ethnicity, sex, count of ambulatory care visits after the index visit, time from the index visit to the last study period visit, and clinic rural or urban status. The BP, HbA_1c_, and LDL analyses also matched on presence of diabetes. Propensity scores were estimated using nearest-neighbor matching with replacement. Analyses of change in total and reversible CVD risk included all patients meeting study inclusion criteria; for other outcomes, analyses were restricted to patients with uncontrolled baseline risk. Residual distributions indicated that linear mixed models were appropriate for all outcomes. All analyses were performed using Stata, version 15.1 (StataCorp LLC).

## Results

### Participants

A total of 18 578 eligible patients were seen at the study clinics during the study period. The mean (SD) age was 58.7 (8.8) years, and there were 9490 (51.1%) women and 9088 (48.9%) men. In terms of race and ethnicity, 4934 (26.6%) were Hispanic, 3351 (18.0%) were non-Hispanic Black, 8434 (45.4%) were non-Hispanic White, 1038 (5.6%) were non-Hispanic in another racial group, and 821 (4.4%) did not have documented race and ethnicity data (Table 1). Randomization (Figure) yielded 42 intervention clinics from 8 organizations (11 159 patients) and 28 control clinics from 7 organizations (7419 patients). Distribution of patient and clinic characteristics differed significantly between intervention and control organizations (3891 [34.9%] vs 4543 [61.2%] non-Hispanic White, respectively; urban clinic location, 35 of 42 [83.3%] vs 15 of 28 [53.6%]) (Table 1 and eTable in Supplement 2).

**Table 1.** Patient Characteristics at Baseline

	Patient group^a^				
	Intervention clinics (n = 11 159)	Intervention clinic subsets by times CV Wizard used			Control clinics (n = 7419)
		Once (n = 2828)	More than once (n = 3939)	Never (n = 4392)	
No. of visits/patient after index visit, mean (SD)	8.6 (7.4)	7.3 (5.6)	11.3 (9.0)	7.0 (5.9)	8.3 (6.9)
Age, mean (SD), y	58.3 (8.9)	58.5 (8.9)	58.9 (8.6)	57.8 (8.9)	59.3 (8.7)
Sex					
Women	5872 (52.6)	1492 (47.2)	2150 (45.4)	2230 (50.8)	3618 (48.8)
Men	5287 (47.4)	1336 (52.8)	1789 (54.6)	2162 (49.2)	3801 (51.2)
Race and ethnicity					
Hispanic	2785 (25.0)	731 (25.9)	676 (17.2)	1378 (31.4)	1196 (16.1)
Non-Hispanic					
Black	2385 (21.4)	631 (22.3)	1196 (30.4)	558 (12.7)	966 (13.0)
White	3891 (34.9)	963 (34.1)	1362 (34.6)	1566 (35.7)	4543 (61.2)
Other^b^	658 (5.9)	161 (5.7)	215 (5.5)	282 (6.4)	380 (5.1)
Unknown	1440 (12.9)	342 (12.1)	490 (12.4)	608 (13.8)	334 (4.5)
Uncontrolled CVD risk					
Blood pressure >140/90	4062 (36.2)	1098 (38.8)	1271 (32.3)	1666 (37.9)	3501 (47.2)
Statin use	5668 (50.8)	1427 (50.5)	2100 (53.3)	2141 (48.8)	4089 (55.1)
BMI >25	9039 (81.0)	2282 (80.7)	3223 (81.8)	3534 (80.5)	5952 (80.2)
Hemoglobin A_1c_ level >8%	1947 (17.5)	461 (16.3)	738 (18.7)	748 (17.0)	1163 (15.7)
Current tobacco use	3177 (28.5)	802 (28.4)	1171 (29.7)	1204 (27.4)	2816 (38.0)
Federal poverty level					
≤138%	6776 (60.7)	1679 (59.4)	2624 (66.6)	2473 (56.3)	3385 (45.6)
>138%	2612 (23.4)	669 (23.7)	823 (20.8)	1120 (25.5)	1062 (14.3)
Unknown	1771 (15.9)	480 (17.0)	492 (12.5)	799 (18.2)	2972 (40.1)
Insurance at index visit					
Uninsured	2016 (18.5)	500 (17.7)	618 (15.7)	943 (21.5)	1165 (15.0)
Medicare	3550 (31.8)	887 (31.4)	1422 (36.1)	1241 (28.3)	2733 (36.8)
Medicaid	3779 (33.9)	951 (33.6)	1278 (32.4)	1550 (35.3)	2186 (29.5)
Private insurance	1312 (11.8)	367 (13.0)	496 (12.6)	449 (10.2)	1256 (16.9)
Other public insurance	457 (4.1)	123 (16.9)	598 (15.2)	209 (4.8)	32 (0.4)
Select diagnoses					
Diabetes	9180 (82.3)	2316 (81.9)	3379 (85.8)	3485 (79.4)	5264 (71.0)
End-stage kidney disease	224 (2.0)	54 (1.9)	79 (2.0)	91 (2.1)	63 (0.9)
Chronic kidney disease	1852 (16.6)	471 (16.7)	753 (19.1)	628 (14.3)	1121 (15.1)
Characteristics that trigger the CDSS alert^c^					
Uncontrolled CVD	2419 (21.7)	562 (19.9)	915 (23.2)	942 (21.5)	1824 (24.6)
Reversible risk >10%	3579 (32.1)	951 (33.6)	1251 (31.8)	1377 (31.4)	3267 (44.0)
Uncontrolled diabetes	9026 (80.9)	2272 (80.3)	3331 (84.6)	3423 (77.9)	5105 (68.8)

Abbreviations: BMI, body mass index (calculated as weight in kilograms divided by height in meters squared); CDSS, clinical decision support system; CVD, cardiovascular disease.

^a^ Unless otherwise indicated, data are expressed as number (%) of patients.

^b^ Includes American Indian or Alaska Native, Asian, Native Hawaiian or Other Pacific Islander, and other race or ethnicity.

^c^ These categories are not mutually exclusive.

**Figure.**
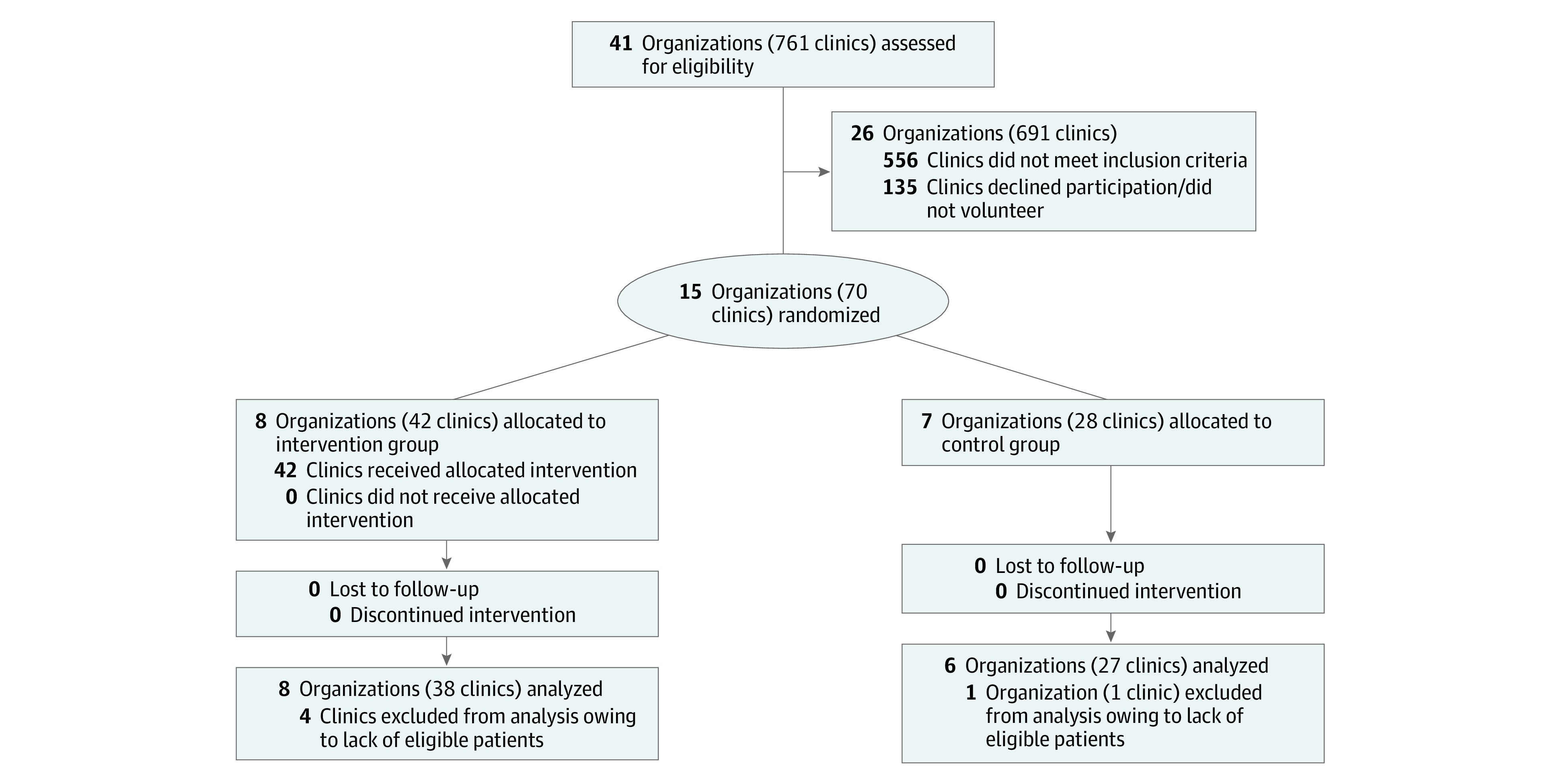
Flow Diagram of Participating Organizations

### Use Rates

CV Wizard was used at 34.7% of index encounters (clinic range, 0%-59.0%). Among patients for whom it was used, it was used a mean of 2.4 (1.9) times during follow-up. It was used at 19.8% of all 91 988 eligible encounters during the study period, including index and follow-up encounters.

### Intervention Impact on 10-Year CVD Risk: ITT Analysis

In the ITT analysis, patients in control clinics had significantly higher mean (SD) baseline 10-year CVD risk (16.6% [12.8%]) than patients in intervention clinics (15.6% [12.3%]; *P* < .001) (Table 2). Change in this risk did not differ significantly between intervention vs control clinics overall. In a subgroup analysis, mean 10-year CVD risk did not improve among patients with baseline CVD risk of less than 20% (risk <10%, 1.8% [95% CI, 1.0-2.5] vs 1.3% [95% CI, 0.7-2.0], *P* < .001; risk of 10% to <20%, 0.3% [95% CI, −0.1 to 0.7] vs 0.6% [95% CI, 0.2-1.0], *P* < .001]). Among those with baseline risk of at least 20%, the mean 10-year risk improved significantly more among patients in control clinics (−1.4% [95% CI, −1.6% to −1.2%]) than those in intervention clinics (−1.0% [95% CI, −1.2% to −0.8%]; *P* = .004).

**Table 2.** Results of ITT Analyses Comparing Intervention and Control Clinic Changes in Measures of CVD Risk During Follow-up Period^a^

CVD measure	Clinic group		*P* value
	Intervention (n = 11 159)	Control (n = 7419)	
**10-y CVD risk results^b^**				
All patients			
No.	10 984	7247	NA
Baseline CVD risk, mean (SD), %	15.6 (12.3)	16.6 (12.8)	<.001
Mean annual change in CVD risk, % (95% CI)	0.4 (0.3-0.5)	0.2 (0.1-0.3)	.001
Patients with baseline CVD risk <10%			
No.	4234	2514	NA
Baseline CVD risk, mean (SD), %	4.4 (3.0)	4.3 (3.2)	.10
Mean annual change in CVD risk, % (95% CI)	1.3 (1.2-1.4)	1.8 (1.6-1.9)	<.001
Patients with baseline CVD risk ≥10% to <20%			
No.	3307	2229	NA
Baseline CVD risk, mean (SD), %	14.7 (2.9)	14.7 (2.9)	.29
Mean annual change in CVD risk, % (95% CI)	0.6 (0.5-0.7)	0.3 (0.1-0.4)	<.001
Patients with baseline CVD risk ≥20%			
No.	3443	2504	NA
Baseline CVD risk, mean (SD), %	30.6 (9.4)	31.1 (9.6)	.06
Mean annual change in CVD risk, % (95% CI)	−1.0 (−1.2 to −0.8)	−1.4 (−1.6 to −1.2)	.004
**Reversible CVD risk results** ^b^				
All patients			
No.	10 984	7247	NA
Baseline reversible risk, mean (SD), %	7.9 (9.0)	9.7 (10.0)	<.001
Mean annual change in reversible risk, % (95% CI)	0.4 (0.3 to 0.5)	−0.1 (−0.3 to −0.02)	<.001
Patients with baseline reversible risk <10%			
No.	7496	4090	NA
Baseline reversible risk, mean (SD), %	3.2 (2.7)	3.4 (2.7)	<.001
Mean annual change in reversible risk, % (95% CI)	1.3 (1.2-1.4)	1.2 (1.1-1.3)	.11
Patients with baseline reversible risk 10% to <20%			
No.	2552	2386	
Baseline reversible risk, mean (SD), %	13.6 (2.8)	13.6 (2.7)	.53
Mean annual change in reversible risk, % (95% CI)	−0.7 (−0.9 to −0.5)	−1.2 (−1.5 to –1.0)	.001
Patients with baseline reversible risk ≥20%			
No.	936	771	NA
Baseline reversible risk, mean (SD), %	29.8 (11.8)	30.7 (13.3)	.11
Mean annual change in reversible risk, % (95% CI)	−4.8 (−5.4 to −4.1)	−4.5 (−5.2 to −3.7)	.54
Patients with high baseline BP			
No.	4035	3503	NA
Baseline, mean (SD), mm Hg			
Systolic	152.5 (15.0)	154.7 (15.2)	<.001
Diastolic	85.0 (11.4)	86.7 (11.6)	<.001
Mean annual change, mm Hg (95% CI)			
Systolic	−7.5 (−7.9 to −7.0)	−8.0 (−8.5 to −7.6)	.10
Diastolic	−3.5 (−3.8 to −3.3)	−3.6 (−3.8 to −3.3)	.84
Patients with high baseline HbA_1c_ level			
No.	1947	1163	NA
Baseline HbA_1c_ level, mean (SD), %	10.2 (1.8)	10 (1.7)	.001
Mean annual change in HbA_1c_ level, % (95% CI)	−0.7 (−0.7 to −0.6)	−0.8 (−0.9 to −0.7)	.16
Patients with high baseline LDL level			
No.	872	570	NA
Baseline LDL level, mean (SD), mg/dL	135.8 (29.5)	136.6 (33.2)	.65
Mean annual change in LDL level, mg/dL (95% CI)	−19.7 (−22.0 to −16.8)	−17.6 (−20.8 to −14.2)	.34

Abbreviations: BP, blood pressure; CVD, cardiovascular disease; HbA_1c_, hemoglobin A_1c_; ITT, intention to treat; LDL, low-density lipoprotein.

SI conversion factors: To convert HbA_1c_ to proportion of total hemoglobin, multiply by 0.01; LDL to mmol/L, multiply by 0.0259.

^a^ Models adjust for age, race and ethnicity, number of visits, rural-urban commuting area, sex, and federal poverty line.

^b^ Excludes patients with new diabetes diagnosis during study period.

### Intervention Impact on 10-Year CVD Risk: ETOT Analysis

In ETOT analyses, among patients with baseline CVD risk of less than 10%, risk did not improve in any CDSS use categories (Table 3). However, the mean change in CVD risk was significantly greater among patients in intervention clinics with a baseline risk of at least 20% (−0.9% [95% CI, −1.2% to −0.7%]) compared with matched control patients (−0.3% [95% CI, −0.5% to −0.1%]; *P* < .001) (Table 4).

**Table 3.** Results of ETOT (per Protocol) Analyses Comparing Intervention and Control Clinic Changes in Measures of CVD Risk During Follow-up Period by Frequency of CV Wizard Use^a^

Patient risk level	Intervention group: tool used once (n = 2828)^b^	Matched control group^c^	*P* value	Intervention group: tool used more than once (n = 3939)^b^	Matched control group^c^	*P* value	Intervention group: tool never used (n = 4392)^b^	Matched control group^c^	*P* value
**10-y CVD risk^c^**									
Baseline risk <10%									
No.	1034	686	NA	1335	674	NA	1865	881	NA
Mean (SD) baseline CVD risk, %	4.4 (2.9)	4.3 (3.1)	.54	4.6 (2.9)	4.3 (3.1)	.03	4.3 (3.0)	4.1 (3.2)	.13
Mean annual change in CVD risk, % (95% CI)	1.3 (1.1-1.5)	1.5 (1.3-1.7)	.19	1.4 (1.3-1.6)	1.6 (1.5-1.8)	.03	1.1 (1.0-1.3)	2.0 (1.9-2.2)	<.001
Baseline risk ≥10%									
No.	1741	1023	NA	2569	1115	NA	2440	1250	NA
Mean (SD) baseline CVD risk, %	23.1 (10.6)	23.6 (11.4)	.27	23.1 (10.5)	24.0 (11.2)	.03	22.2 (10.7)	22.9 (11.1)	.10
Mean annual change in CVD risk, % (95% CI)	−0.6 (−0.9 to −0.3)	−0.8 (−1.1 to −0.6)	.23	<0.1 (−0.2 to 0.2)	−0.4 (−0.6 to −0.2)	.002	−0.4 (−0.6 to −0.2)	−0.9 (−1.1 to −0.7)	.001
**Reversible CVD risk^d^**									
Baseline risk <10%									
No.	1855	1125	NA	2673	1120	NA	2968	1441	NA
Mean (SD) baseline reversible risk, %	3.1 (2.7)	3.2 (2.6)	.32	3.4 (2.7)	3.2 (2.7)	.02	3.1 (2.7)	3.2 (2.7)	.25
Mean annual change in reversible risk, % (95% CI)	1.2 (1.1-1.4)	1.3 (1.2-1.5)	.29	1.5 (1.3-1.6)	1.7 (1.5-1.8)	.02	0.9 (0.8-1.1)	1.1 (1.0-1.2)	.09
Baseline risk ≥10%									
No.	920	633	NA	1231	604	NA	1337	847	NA
Mean (SD) baseline reversible risk, %	17.6 (8.6)	17.7 (10.3)	.84	18.9 (10.2)	18.3 (9.8)	.17	17.3 (9.9)	17.7 (9.7)	.38
Mean annual change in reversible risk, % (95% CI)	−2.4 (−2.9 to −1.8)	−2.3 (−2.8 to −1.8)	.83	−1.7 (−2.0 to −1.3)	0.1 (−0.5 to −0.2)	<.001	−2.4 (−2.8 to −1.9)	−2.9 (−3.3 to −2.5)	.10
High baseline blood pressure									
No.	1098	725	NA	1271	723	NA	1666	973	NA
Mean (SD) baseline, mm Hg									
Systolic	152.6 (15.2)	154.9 (15.5)	.002	152.3 (14.6)	152.6 (14.0)	.73	152.5 (15.3)	154.3 (15.4)	.003
Diastolic	85.5 (11.2)	87.1 (12.4)	.01	84.6 (11.2)	86.1 (11.2)	.003	85.0 (11.6)	86.9 (12.1)	<.001
Mean annual change, mm Hg (95% CI)									
Systolic	−8.3 (−9.2 to −7.3)	−8.3 (−9.3 to −7.3)	.99	−6.6 (−7.3 to −6.0)	−6.1 (−6.7 to −5.4)	.22	−9.6 (−10.4 to −8.8)	−9.4 (−10.2 to −8.6)	.76
Diastolic	−3.9 (−4.4 to −3.4)	−3.4 (−3.9 to −2.8)	.13	−3.2 (−3.5 to −2.8)	−2.5 (−2.8 to −2.2)	.01	−4.5 (−5.0 to −4.1)	−3.9 (−4.4 to −3.5)	.07
High baseline HbA_1c_ level									
No.	461	284	NA	738	315	NA	748	425	NA
Mean (SD) baseline HbA_1c_ level ,%	10.2 (1.8)	10.2 (1.7)	.83	10.2 (1.8)	10.0 (1.7)	.15	10.3 (1.8)	10.0 (1.7)	.005
Mean annual change in HbA_1c_ level, % (95% CI)	−0.5 (−0.7 to −0.4)	−1.0 (−1.2 to −0.9)	<.001	−0.7 (−0.8 to −0.6)	−1.0 (−1.0 to −0.8)	<.001	−0.9 (−1.0 to −0.8)	−0.9 (−1.0 to −0.8)	.94
High baseline LDL level									
No.	244	163	NA	288	124	NA	340	198	NA
Mean (SD) baseline LDL level, mg/dL	135.8 (30.2)	135.7 (29.6)	.98	134.0 (28.8)	135.3 (31.6)	.69	137.4 (29.7)	134.4 (26.7)	.23
Mean annual change in LDL level, mg/dL (95% CI)	−20.8 (−26.0 to −15.7)	−18.2 (−22.5 to −13.8)	.44	−18.6 (−22.9 to −14.2)	−17.1 (−21.1 to −13.0)	.62	−21.7 (−26.1 to −17.4)	−19.2 (−23.2 to −15.2)	.41

Abbreviations: CVD, cardiovascular disease; ETOT, effect of treatment on the treated; HbA_1c_, hemoglobin A_1c_; LDL, low-density lipoprotein; NA, not applicable.

SI conversion factors: To convert HbA_1c_ to proportion of total hemoglobin, multiply by 0.01; LDL to mmol/L, multiply by 0.0259.

^a^ Regression models included random effects for clinic and patient.

^b^ Counts of tool use exclude use at the last visit of the study period.

^c^ Patients matched on race and ethnicity, sex, age, baseline federal poverty level, rural-urban commuting area, ambulatory visits to a study clinic during the study period, time from index visit to last visit, and baseline score for each outcome.

^d^ Excludes patients diagnosed with diabetes during the study period.

**Table 4.** Results of ETOT (per Protocol) Analyses Comparing Intervention and Control Clinic Changes in CVD Risk During Follow-up Period, Stratified by Baseline CVD Risk^a^

	Clinic group		*P* value
	Intervention	Matched control^b^	
**CVD risk**			
Patients with baseline 10-y CVD risk <10%			
No.	2369	852	NA
Mean (SD) baseline CVD risk, %	4.5 (2.0)	4.3 (3.1)	.06
Mean annual change in CVD risk, % (95% CI)	1.4 (1.3-1.5)	1.5 (1.4-1.6)	.10
Patients with baseline 10-y CVD risk 10% to <20%			
No.	2032	734	NA
Mean (SD) baseline CVD risk, %	14.8 (2.9)	14.8 (2.9)	.78
Mean annual change in CVD risk, % (95% CI)	0.7 (0.5-0.8)	0.4 (0.3-0.6)	.02
Patients with baseline 10-y CVD risk ≥20%			
No.	2274	757	NA
Mean (SD) baseline CVD risk, %	30.6 (9.2)	31.4 (10.0)	.04
Mean annual change in CVD risk, % (95% CI)	−0.9 (−1.2 to −0.7)	−0.3 (−0.5 to −0.1)	<.001
**Reversible CVD risk**			
Patients with baseline reversible risk <10%			
No.	4528	1694	NA
Mean (SD) baseline reversible risk, %	3.3 (2.7)	3.4 (2.7)	.45
Mean annual change in reversible risk, % (95% CI)	1.4 (1.3-1.5)	1.2 (1.1-1.3)	.008
Patients with baseline reversible risk 10% to <20%			
No.	1521	719	NA
Mean (SD) baseline reversible risk, %	13.7 (2.8)	13.6 (2.7)	.15
Mean annual change in reversible risk, % (95% CI)	−0.7 (−0.9 to −0.4)	−0.2 (0.5 to 0.04)	.04
Patients with baseline reversible risk ≥20%			
No.	630	243	NA
Mean (SD) baseline reversible risk, %	29.5 (10.9)	30.7 (13.1)	.18
Mean annual change in reversible risk, % (95% CI)	−4.4 (−5.2 to −3.7)	−2.7 (−3.4 to −1.9)	.001

Abbreviations: CVD, cardiovascular disease; ETOT, effect of treatment on the treated; NA, not applicable.

^a^ Group 1 patients who had the tool used at least once with baseline risk score in each baseline risk group were matched to group 2 patients with baseline risk score in same range.

^b^ Matched on age, race and ethnicity, rurality, number of visits, time from first to last visit, federal poverty level, sex, and diabetes diagnosis.

### Intervention Impact on 10-Year Reversible CVD Risk: ITT Analysis

Patients in control clinics had significantly higher mean (SD) baseline reversible risk (9.7% [10.0%]) than those in intervention clinics (7.9% [9.0%]; *P* < .001). Overall, in ITT analyses, reversible risk improved significantly more among patients in control clinics (mean, −0.1% [95% CI, −0.3% to −0.02%]) compared with patients in intervention clinics (mean, 0.4% [95% CI, 0.3%-0.5%]; *P* < .001).

In ITT analyses, change in reversible risk did not differ across study groups in patients with baseline risk of less than 10% or at least 20%. Reversible risk improved significantly more among patients in control clinics (mean, −1.2% [95% CI, −1.5% to –1.0%]) compared with patients in intervention clinics (mean, −0.7% [95% CI, −0.9% to −0.5%]; *P* = .001) with a baseline reversible risk of at least 10% to less than 20% (Table 2).

### Intervention Impact on 10-Year Reversible CVD Risk: ETOT Analysis

In ETOT analyses, no improvement was seen among patients with a baseline reversible CVD risk of less than 10%, regardless of tool use. Among patients with a baseline risk of at least 10%, no significant differences were seen when the tool was used once or never (Table 3), but when it was used more than once, reversible CVD risk for patients in intervention clinics improved significantly more than that of patients in control clinics (mean, −1.7% [95% CI, −2.0% to −1.3%] vs 0.1% [95% CI, −0.5% to −0.2%]; *P* < .001). When stratified, no improvement was seen among those with baseline risk of less than 10% (Table 4), but patients in intervention clinics improved significantly more than patients in control clinics among those with baseline risk of at least 10% to less than 20% (mean, −0.7% [95% CI, −0.9% to −0.4%] vs −0.2% [95% CI, −0.5% to 0.04%], respectively; *P* = .04) and at least 20% (mean, −4.4% [95% CI, −5.2% to −3.7%] vs −2.7% [95% CI, −3.4% to −1.9%], respectively; *P* = .001).

### Impact on Specific CVD Risk Factors

In ITT analyses, no significant difference was seen in change in systolic BP or diastolic BP (DBP) or in HbA_1c_ or LDL levels among those with high baseline measures of each biomarker (Table 2). In ETOT analyses, LDL levels did not improve significantly more in patients in intervention or control clinics in any tool use categories. Although mean DBP improved significantly more in patients in intervention clinics (−3.2 [95% CI, −3.5 to −2.8] mm Hg) than in control clinics (−2.5 [95% CI, −2.8 to −2.2] mm Hg; *P* = .01) when the tool was used more than once, this difference was not clinically significant. Mean levels of HbA_1c_ decreased significantly less among patients for whom the tool was used once (−0.5% [95% CI, −0.7% to −0.4%]) compared with controls (−1.0% [95% CI, −1.2% to −0.9%]; *P* < .001) and among those for whom it was used more than once (−0.7% [95% CI, −0.8% to −0.6%]) compared with controls (−1.0% [95% CI, −1.0% to −0.8%]; *P* < .001). We note that for most patients with more than 1 uncontrolled risk factor, the CDSS would emphasize more effective ways to reduce CVD risk than tightening control of HbA_1c_ levels.

## Discussion

CV Wizard was effective in integrated care settings primarily serving insured patients; this trial assessed its impact in CHCs. Because CDSS use rates affect population-level outcomes, ITT and ETOT analyses were conducted. In ITT results, total CVD risk did not improve significantly more in patients in intervention clinics overall but improved more among patients in intervention than control clinics among those with a baseline risk of greater than 20%. No consistent, significant impact on reversible CVD risk was seen. In ETOT analyses, however, among patients for whom the tool was used at least once, CVD risk decreased significantly more among those in the highest baseline risk group compared with controls. Although this risk reduction was modest (absolute improvement of 4.4% vs 2.7%), if maintained over time it could represent a population-level reduction in cardiovascular events.^57,58^ Among those with a baseline reversible risk of at least 10%, intervention patients improved significantly more than controls when the tool was used more than once, suggesting a possible dose-response effect.

The ITT results are unsurprising given the overall tool adoption rate and may explain why these findings contrast with the largely positive earlier findings for this CDSS in better-resourced health care systems.^33,34,59^ In those studies, use of CV Wizard improved glucose levels and BP control in adults with diabetes, overall CVD risk in adults without diabetes or heart disease,^33,34^ and BP management in patients aged 6 to 18 years.^59^ In those studies, however, CDSS results were printed at 70% to 80% of targeted encounters. Many other CDSS studies were unable to demonstrate impact owing to low adoption^23,59,60^ (eg, a recent implementation in Belgium that had single-digit use rates and no improvement in targeted outcomes).^53^

Factors that affect point-of-care CDSS use include workflow integration, competing clinical demands, number of clicks to access the CDSS, and clinician confidence in the validity of the advice provided.^23,24^ This CDSS achieved much higher use rates in centralized care systems with established tool use workflows. The present study included numerous care organizations in which heterogeneity in rooming protocols impeded training and sustained high CDSS use. Future studies should identify strategies for increasing CDSS use in CHCs. Analyses designed to understand CDSS adoption in this setting will be presented in future reports.

### Limitations

In this cluster randomized clinical trial, randomization accounted for organization size, but could not balance on other characteristics, so analyses controlled for baseline factors likely to affect outcomes. Other variables may have affected outcomes. In addition, intraclass correlation coefficients for key study outcomes indicated high heterogeneity across intervention and control clinics, which dilutes power to detect intervention effects. This study was conducted in a heterogenous network of CHCs sharing a single EHR. Extrapolation to different settings requires caution. CV Wizard supports both CDS and shared decision-making, but these analyses did not assess which elements were used. Similarly, even if the tool’s output was viewed or printed, we do not know how it was used to engage individual patients; however, further analyses are underway.

## Conclusions

This CDSS intervention appeared to have a benefit for CVD risk when it was consistently used for CHC patients with high baseline risk. Future research is needed on how CDSS tools are used in clinical encounters and to develop strategies to increase CDSS use in CHCs and similar settings. Despite limitations, these results provide preliminary evidence that this technology has the potential to improve clinical care among CHC patients with high CVD risk.

## Notes

Supplement 1.
Trial Protocol

Supplement 2. **eFigure 1.** CV Wizard Provider View (Prioritizes Patient Risks and Provides Decision Support Suggestions) **eFigure 2.** CV Wizard Patient View (Prioritizes Patient Risks and Presents the Information Visually) **eMethods.** Reversible Risk Calculations **eTable.** Clinic Characteristics by Study Group at Baseline
eReferences

Supplement 3.
Data Sharing Statement
