# Supplementary material for: Effect of Clinical Decision Support at Community Health Centers on the Risk of Cardiovascular Disease: A Cluster Randomized Clinical Trial
Source: JAMA Netw Open. 2022 Feb 4;5(2):e2146519. doi: 10.1001/jamanetworkopen.2021.46519 (PMC8817199; doi:10.1001/jamanetworkopen.2021.46519)
Supplement: Supplement 1. — Trial Protocol [file jamanetwopen-e2146519-s001.pdf]

INSTRUCTIONS:

1. Protocol Title

CV Wizard: Does a Prioritized, Point-of-Care Clinical Decision Support Tool Improve Guideline-Based CVD Risk Factor Control in Safety Net Clinics?

2. Objectives

This project aims to reduce disparities in cardiovascular disease (CVD) risk factor control and in rates of heart attacks and strokes among the low-income, racially / ethnically diverse Americans who receive primary care at safety net community health centers (CHCs). To achieve this objective, we will adapt a successful clinical decision support (CDS) system (CV WIZARD), currently used in CVD care at several large, integrated health care systems) to meet the patient needs and workflow processes of 60 CHCs. We will determine if use of this CDS improves CVD care, reduces disparities in CVD care and outcomes, and increases patient engagement in CVD treatment choices, in CHCs. The goal is to address gaps in guideline-based care in high-risk populations with targeted, innovative, multi-level strategies; considering setting-specific needs; and supporting patient engagement.

Our overarching aims are to:

Aim 1. Conduct a clinic-randomized trial of the impact of an evidence-based point-of-care CDS system on (i) overall CVD risk scores, and (ii) control of individual CVD risks (blood pressure; HbA1c, lipid levels; aspirin use; smoking; body mass index), among high CVD risk CHC adult patients.

Human related components and activities: We will recruit 60 Community Health Clinics (CHCs) to participate in either Arm 1 (N=30) or Arm 2 (N=30) of the study. Recruitment efforts may include verbal conversations and/or email conversations with CHC leadership, via OCHIN's routine process for research studies. We will submit a study modification with examples of recruitment materials, as requested by the IRB, prior to recruitment efforts.

Aim 2. Develop and hone need-based implementation support protocols to help Arm 1 CHCs implement the CV Wizard CDS system into their standard workflows; assess whether use of the protocols developed for Arm 1 CHCs accelerates implementation and adoption of the CDS system in the Arm 2 CHCs.

Human related components and activities: Provide coaching and practice facilitation for clinic staff in Arm 1 and Arm 2.

Aim 3. Conduct a mixed methods process evaluation, guided by the Technology Acceptance Model, to identify and address patient, provider, and delivery system barriers to uptake / impact of this CDS in CHCs.

Human related components and activities: We will conduct semi structured phone interviews with clinic staff in Arm 1. We will recruit and conduct qualitative data collection in two Arm 1 case study clinics. We will conduct in-person semi structured interviews with clinic staff in Arm 1. We will conduct observation of relevant workflows, patient encounters, and clinic meetings. We will collect relevant artifacts (process maps, communications, etc.) We will conduct 10 patient interviews with patients whose most recent office visit involves CV WIZARD. We will submit a study modification with examples of recruitment materials, as requested by the IRB. No PHI will be collected during any of these activities. We will request submit a study modification with study fact sheets, examples of structured interview questions for CHC staff and patients, etc., prior to qualitative data collection.

3. Background

Substantial progress in reducing cardiovascular disease (CVD) morbidity and mortality would be achieved if evidence-based guidelines for CVD risk factor control were implemented consistently in primary care settings. Electronic health record (EHR)-based clinical decision support (CDS) systems that identify uncontrolled CVD risk factors and provide individualized care recommendations improved rates of guideline-concordant CVD care in large, integrated healthcare settings, but little is known about how effective such CDS may be in safety net community health centers (CHCs). CHCs' socioeconomically vulnerable patients have far worse CVD risk factor control and higher rates of major CVD events than the general population. Implementing CDS that leads to improved CVD risk factor control in CHCs could reduce national disparities in CVD outcomes, but CHCs rarely have the resources to develop sophisticated CDS, and very few currently have such systems for CVD care. The proposed study is designed to address this.

Please refer the end of this document for a bibliography.

4. Study Design

We will randomize 60 safety net community health centers (CHCs) to implement CV Wizard either at the start of study year 2 (Arm 1, N=30), or 18 months later (Arm 2, N=30).

CV WIZARD is a clinical decision support (CDS) tool that provides point-of-care cardiovascular disease (CVD) care recommendations to the primary care provider and the patient. CV Wizard identifies a patient's uncontrolled CVD risk factors, prioritizes those factors based on potential CVD risk reduction for that patient, and generates specific guideline-based treatment recommendations for each uncontrolled risk factors.

This study affects human subjects because we will interview CHC staff and patients to determine if use of CV WIZARD improves CVD care, reduces disparities in CVD care and outcomes, and increases patient engagement in CVD treatment choices, in CHCs. No PHI will be collected during these interviews.

We will compare outcomes from Arm 1 (immediate implementation) and Arm 2 (delayed implementation); this will enable us to measure the intervention's impact on CVD risk factor control in CHCs. Arm 1 CHCs will receive implementation support to address any barriers to adoption / sustained use of the CDS that are identified through study activities. We will apply these learnings to improve adoption rates in Arm 2.

Through this design, we will: 1) assess whether a CDS tool is effective in CHCs, 2) identify and address barriers to effectiveness and adoption, and 3) fine-tune strategies to support implementation of sophisticated CDS systems in CHCs. Mixed methods analyses will identify factors affecting uptake of CV WIZARD.

5. Study Population

a. Number of Subjects

This study does not include any KP subjects. Instead we will randomize 60 CHCs from within the OCHIN membership with a shared EHR, to immediate (Arm, N=30) vs. delayed (Arm 2, N=30) implementation.

b. Inclusion and Exclusion Criteria

Inclusion Criteria:

- Adult clinic attendees with high CVD risk, including women and minorities
- Persons aged 18-21 with high-CVD risk
- May include some subjects with mental health conditions of various types; however, it is important to systematically address CV risk in this population, because such patients may be at risk for elevated CV risk and because such patients often have been excluded or underrepresented in previous research studies.

Exclusion Criteria:

- Children aged younger than 18

Note: The investigators are not enrolling patients for this clinic-randomized study, but rather studying the uptake and impact of a set of EHR-based clinical decision support tools into regular care at the participating clinics. In this clinic-randomized trial, the intervention / randomization are clinic level. The intervention targets clinic processes that are part of the regular care patients receive, and will not require special visits.

The numbers below show adult patients with hypertension (as a proxy for CVD risk) as of August 2015, in 60 clinics from the pool of clinics from which we will recruit.

Comments:

In this clinic-randomized trial, patients will not be 'recruited.' The intervention / randomization are clinic level. The intervention targets clinic processes that are part of the regular care patients receive, and will not require special visits. The trial targets high CVD risk adults seen in 60 study clinics. The numbers below show adult patients with hypertension (as a proxy for CVD risk) as of August 2015, in 60 clinics from the pool of clinics from which we will recruit.

Racial Categories	Ethnic Categories									
	Not Hispanic or Latino			Hispanic or Latino			Unknown/Not Reported Ethnicity			Total
	Female	Male	Unknown/ Not Reported	Female	Male	Unknown/ Not Reported	Female	Male	Unknown/ Not Reported	
American Indian/ Alaska Native	297	224		141	107					769
Asian	1187	895		0	0					2082
Native Hawaiian or Other Pacific Islander	297	224		141	107					769
Black or African American	4746	3580		2264	1708					12298
White	20171	15217		9620	7258					52266
More than One Race	0	0		0	0					0
Unknown or Not Reported										
Total	26698	20140		12166	9180					68184

c. Vulnerable Populations

Persons aged 18-21 will be included if they fall into the targeted group of high-CVD risk patients. Children aged younger than 18 will not be included, as different clinical guidelines often apply to children with CVD risk.

The study will not preferentially recruit any special populations. All affected patients subjects will be adults. This may include some subjects with mental health conditions of various types; however, it is important to systematically address CV risk in this population, because such patients may be at risk for

elevated CV risk and because such patients often have been excluded or underrepresented in previous research studies.

d. Setting

As with past CHR and OCHIN collaborative research studies (e.g. OCHIN ALL, SPREAD-NET, ASSESS & DO), OCHIN staff will recruit a total of 60 member clinics (from the pool of eligible clinics) to take part in CV WIZARD.

OCHIN, Inc., is a non-profit, community-based health center-controlled network. Its members (>480 CHCs, in 19 states) share characteristics of other CHCs, so results will be generalizable to many CHCs. As the nation's largest CHC network with a single EHR system, OCHIN pioneered the development of EHR tools for CHCs. OCHIN's member CHCs share a single Epic© EHR, which is unduplicated, centrally maintained, and network-wide. Data are standardized and quality-checked: thus, validated data are already linked between all study sites.

HealthPartners Institute is one of the largest medical research and education centers in the Midwest. As part of HealthPartners integrated care system, the Institute uses research and education to accelerate improvements in quality, experience and affordability for our members, patients and the community. Dr. Patrick O'Connor, a study team member, has conducted substantial research to develop CV Wizard, and implement it in integrated care delivery settings. CV Wizard's precursor – DM Wizard – significantly improved A1c control and aspects of BP control in diabetes patients at HealthPartners. CV Wizard, the next generation of this CDS system, substantially builds on DM Wizard. Neither DM Wizard nor CV Wizard has ever been implemented in the CHC setting, but as use rates, provider satisfaction, and impact on care in integrated care settings were exceptional. CV Wizard is now used to support optimal cardiometabolic care for 1,500,000 patients in 3 large care delivery systems. CV WIZARD builds on two groups of researchers' preliminary data on (i) optimizing CV Wizard in an integrated care setting, and (ii) implementing EHR-based CDS in CHCs.

Both OCHIN and Health Partners will rely on CHR's IRB for IRB review. Please see the 'cede letter' from Health Partners - attached in the IRQ as a supporting document. OCHIN will submit their letter after we are funded.

e. Recruitment Methods

CHC Recruitment*:* OCHIN's Community Research Associate has expertise in operational leadership with an emphasis on projects that serve low-income populations. During Year 1, she will work with KPCHR and the OCHIN research team to recruit 60 study CHCs (from the baseline pool of CHCs that annually provide primary care to >35 adults with high CVD risk, and have been using OCHIN's EHR for >18 months). OCHIN's clinical leadership (including CEOs of its member CHC organizations) may also help with recruitment, as needed. Recruitment will be targeted to optimize diversity in clinic baseline characteristics.

OCHIN utilizes a variety of recruitment methods when they invite CHCs to participate in research studies. For example, recruitment activities may involve a verbal conversation with CHCs (that they have standing relationships with), or possibly sending an introductory email (to newer CHCs or those they don't know well). Another possibility is announcing the study in a standing Clinical Operations Review Committee (CORC) meeting and asking for interested CHCs to identify themselves offline.

For many study CHCs, recruitment will simply involve agreeing to be randomized to have the CDS tool 'turned on' at their site at study month 13 or 31. This falls within OCHIN's normal QI activities. Another possibility is that once a clinic agrees to participate, OCHIN's Community Research Associate may follow up with a Memorandum

of Understanding (MOU) with the study timeline, expectations, etc. This MOU is typically signed by OCHIN's leadership, the Study PI and leadership at the participating CHC.

Similarly, once recruitment for each Arm is complete, OCHIN's Community Research Associate will identify and engage four (4) (two per arm) of the study CHCs to be a case study site.

CHC Staff recruitment:

Phone interviews: OCHIN's Community Research Associate, working by email and phone with clinic contacts identified during the recruitment process, will identity and recruit one staff member for a single phone interview at each of 20 clinics with extensive experience using CV Wizard in patient care. OCHIN's Community Research Associate will perform similar liaison and recruitment activities for any necessary follow-up interviews.

In-person interviews and observations at 4 case study clinics: Each case study clinic will be asked to appoint a member of the clinic staff to be the liaison between researchers and staff. The KPCHR qualitative research team will work with this person to identify and recruit appropriate staff to observe and interview over the 1.5-2.5 years of data collection.

CHC Patient recruitment: We will seek patient input as follows.

Patients from OCHIN's Patient Engagement Panel (PEP): this panel includes >20 patients from OCHIN member CHCs, who meet regularly to contribute input on research conducted at OCHIN. The study team will meet with the PEP monthly to engage them in a user-centered process on whether and how to redesign the CV Wizard 'Patient View'.

Patients from the study CHCs: Post implementation, the qualitative team will interview 20 patients (who were given the CV Wizard CVD risk assessment) to identify barriers / facilitators to the tool's use from the patient perspective. Participation will be completely voluntary – on days the researchers are in clinic (one of the four case study clinics) clinic providers will tell patients (with whom they used the CV Wizard tool) that a researcher would be interested in talking with them about their perceptions of the acceptability / utility of CV Wizard and related discussion with the provider; if interested the patient will approach the researcher.

f. Consent Process

CHC staff interviews: We are planning to record the staff interviews and will ask CHC staff for verbal consent, prior to recording these interviews. No PHI will be collected during the staff interviews. As the risk to CHC staff is minimal, we will request IRB approval to provide study fact sheets and to ask for verbal consent for all qualitative data collection from CHC staff. We will submit the study fact sheets as a study modification, prior to distribution to CHC staff.

CHC clinic observations: At the four case study clinics, we will conduct observation of workflows, patient encounters, and clinic meetings relevant to CV Wizard. These observations will not be audio recorded; researchers will take field notes (although PHI may be observed, no PHI will be written down). As with staff interviews, we will request IRB approval to provide study fact sheets and obtain verbal consent from participants. No PHI will be collected during the clinic observations. As the focus of any observations during patient encounters will be provider/staff behavior and interaction with CV Wizard, and a signed consent form would be the only document linking the patient with the study data, we will ask for verbal (not written) patient consent to observe patient encounters.

CHC patient consent: We will interview a sample of patients who receive the CV Wizard CVD risk summary, to assess their experience with it. No PHI will be collected during patient interviews. Participation will be completely voluntary – providers will let patients with whom they used the CV Wizard tool that a researcher would be interested in talking with them about their perceptions of the acceptability / utility of CV Wizard and related discussion with the provider. Because a signed consent form would be the only document linking the patient's name with the interview data, we will request IRB approval to provide a study fact sheet and obtain recorded verbal consent from interviewees prior to data collection.

6. Study Procedures

Year 1: Months 1 – 12

- Conduct baseline assessment of all potential CHCs.
- Recruit 60 CHCs to participate in the study.
- Randomize 60 CHCs to implement CV WIZARD in Arm 1 (N=30) or Arm 3 (N=30).
- Link CV WIZARD to OCHIN's EHR.
- Conduct extensive validity testing.
- Refine how the Health Partners system performs with OCHIN EHR data.
- Develop training materials.
- Engage patient stakeholders to adapt patient-facing aspect of CV WIZARD to meet needs of CHC patients.

Year 2 - 3: Months 13 – 30

- Implement CVD WIZARD in Arm 1.
  - Provide CHCs with iterative, need-based training and implementation support.
- Provide coaching and practice facilitation for clinic staff in Arm 1.
- Conduct mixed-methods process evaluation of factors affecting CV WIZARD's uptake:
  - Conduct semi structured phone interviews with clinic staff in Arm 1.
  - Recruit and conduct qualitative data collection in two Arm 1 case study clinics.
  - Conduct in-person semi structured interviews with clinic staff in Arm 1.
  - Conduct observation of relevant workflows, patient encounters, and clinic meetings.
  - Collect relevant artifacts (process maps, communications, etc.)
  - Conduct 10 patient interviews with patients whose most recent office visit involve CV WIZARD.
  - Begin iterative qualitative analysis.

Year 3 - 4: Months 31 –60

- Implement CVD WIZARD in Arm 2 (18 months after Arm 1 implementation).
  - Provide training/ support, as fine tuned in Arm 1.
  - Adapt further support as indicated.
- Provide coaching and practice facilitation for clinic staff in Arm 1 and Arm 2.
- Conduct mixed-methods process evaluation of factors affecting CV WIZARD's uptake:

    - Conduct semi structured phone interviews with clinic staff in Arm 2.
    - Recruit and conduct qualitative data collection in two Arm 2 case study clinics.
    - Conduct in-person semi structured interviews with clinic staff in Arm 2.
    - Conduct observation of relevant workflows, patient encounters, and clinic meetings.
    - Collect relevant artifacts (process maps, communications, etc.)
    - Conduct 10 patient interviews with patients whose most recent office visit involve CV WIZARD.
    - Begin iterative qualitative analysis.
- Assess sustainability and differences in rate and speed of uptake across arms.

Year 5: Months 49 –60

- Analyze qualitative and quantitative data on CV WIZARD tool use and outcomes
- Create CVD WIZARD implementation guide for use by any CHCs

Quantitative data points: Below are details about the specific outcomes and measures for each. Please refer to Table 6 further below for additional information.

Primary Outcome Measure:

1. **Comparison of CVD outcomes for patients in CHCs**
    - Compare patients' CVD outcomes in the 30 Arm 1 vs. 30 Arm 2 CHCs, in months 13-30 (Aim 1); assess whether the revised implementation support materials expedite CDS adoption (Aim 2); and use mixed methods to identify multi-level barriers / facilitators to adoption of the CDS tool (Aim 3).

Secondary Outcome Measures:

2. **Reach: Encounters affected**
    - % clinic encounters where CV Wizard suggests running the full risk assessment tool, i.e., identified a target patient.

3. **Effectiveness (impact): Patient outcomes - 10 year pooled ASCVD risk score**
    - ASCVD risk score (American College of Cardiology-ACC/ American Heart Association - AHA) 10-year pooled ASCVD risk score, 40-75 year olds

4. **Effectiveness (impact): Patient outcomes - Framingham 30-year CVD risk score**
    - Framingham 30-year CVD risk score, 20-39 year olds.

5. **Effectiveness (impact): Patient outcomes - last BP ≤140/90**
    - Control of individual CVD risk factors: last BP ≤140/90

6. **Effectiveness (impact): Patient outcomes - last A1c≤8**
    - Control of individual CVD risk factors: last A1c≤8

7. **Effectiveness (impact): Patient outcomes - last LDL<100**
    - Control of individual CVD risk factors: last LDL<100

8. **Effectiveness (impact): Patient outcomes - appropriate aspirin use**
    - Control of individual CVD risk factors: appropriate aspirin use

9. **Effectiveness (impact): Patient outcomes - not current smoker**
    - Control of individual CVD risk factors: not current smoker

10. **Effectiveness (impact): Patient outcomes - last BMI ≤25**
    - Control of individual CVD risk factors: last BMI≤25

11. **Effectiveness (impact): Patient outcomes - appropriate cardioprotective medications (e.g., statins)**
    - Control of individual CVD risk factors: appropriate cardioprotective prescriptions (e.g., statins)

12. **Adoption: CDS uptake**
    - % of encounters where care team member opts to run the CV Wizard risk assessment.

13. **Implementation: User perceptions**
    - Perceived ease of use, usefulness, acceptability of CV Wizard; intent to use it.

14. **Maintenance over time**
    - All measures over 2.5 years of follow-up, Arm 1; 1.5 years, Arm 2.

Table 6. Impact and uptake of the CV Wizard system: Quantitative Measures. See Appendix D for details.

**Outcomes, per RE-AIM**	**Measurement**
**Reach:** Encounters affected	% clinic encounters where CV Wizard suggests running the full risk assessment tool, *i.e.*, identified a target patient
**Effectiveness (impact):** Patient outcomes	**(i) ASCVD risk score** (ACC/AHA 10-year pooled ASCVD risk score, 40-75 year olds; Framingham 30-year CVD risk score, 20-39 year olds)[64-67]. **(ii) Control of individual CVD risk factors**: last BP ≤140/90; last A1c≤8; last LDL<100; appropriate aspirin use; not current smoker; last BMI≤25); appropriate cardioprotective prescriptions (*e.g.*, statins)
**Adoption**: CDS uptake	% of encounters where care team member opts to run the CV Wizard risk assessment
**Implementation:** User perceptions	Perceived ease of use, usefulness, acceptability of CV Wizard; intent to use it; see 3.3.f.ii
**Maintenance** over time	All measures over 2.5 years of follow-up, Arm 1; 1.5 years, Arm 2
**Potential covariates**	**Measurement**
Patient demographic characteristics	Age; gender; race / ethnicity; primary language; poverty level; insurance status at visit; # visits to that site / provider in last year. Others TBD based on input from clinician advisors.
Other patient comorbid conditions	Renal function: Impaired if eGFR < 60 cc/min/1.72m2, or microalbumin / creatinine ratio ≥30 or equivalent; Charlson Comorbidity Score (Modified): Indicator of serious comorbid conditions that may shorten life expectancy, modified to exclude CV components; depression 296.xx, 311.xx, or prescribed antidepressant. Other relevant comorbidities TBD.
Visit type	DM as chief complaint YN; CVD as chief complaint YN. Others TBD.
Provider type	Degree (MD, RN, PA, etc.); Prescribing privileges YN; # in patient panel
Other clinic factors	See Table 5; data from baseline survey
**CVD care / process**	**Measurement**
Recommended care that is ordered.	Whether care suggestions were acted on within 7 days of the encounter; *e.g.*, if CV Wizard suggests starting a statin, we will ask whether a prescription was issued.

[illegible]

<u>Qualitative:</u> We will take a 3-tiered approach to qualitative data collection. Below is a detailed description of the process and potential data points.

All clinics: Collect i) baseline data on clinic characteristics ii) all ‘trouble-tickets’ relevant to CV Wizard submitted to OCHIN via its member help request system, and all user feedback submitted via CV Wizard’s feedback mechanism; and iii) all exchanges from the implementation support webinars; iv) periodically debrief with the practice facilitator and others involved with implementation support.

Selected clinics: Conduct semi-structured phone interviews with one care team member involved with implementing / using CV Wizard, at 20 clinics (10 per Arm), about 10 months post-implementation. Sites

will be purposively sampled for diversity in CV Wizard use rates. Follow-up interviews will be conducted as needed, to explore perceptions, acceptance, and use of CV Wizard and the implementation support provided. Prior to data collection, we will submit a study modification with an example of structured interview questions for CHC staff.

Case study clinics: We will recruit two clinics per Arm for in-depth ethnographic case study, which uses observation / personal interaction to explore the dynamics underlying implementation outcomes. We will follow Arm 1 clinics for 2.5 years, and Arm 2 clinics for 1.5 years. Methods will include naturalistic observation (of workflows, patient encounters, clinic meetings), key informant and in-depth interviews, and collecting relevant artifacts (process maps, communications, etc.) Prior to data collection, we will submit a study modification with an example of in-depth interview questions for CHC staff.

Data Analysis

a. Analysis Plan

In this clinic-randomized pragmatic trial, 60 CHCs will implement CV Wizard via a staggered process. We will compare patients' CVD outcomes in the 30 Arm 1 vs. 30 Arm 2 CHCs, in months 13-30 (Aim 1); assess whether the revised implementation support materials expedite CDS adoption (Aim 2); and use mixed methods to identify multi-level barriers / facilitators to adoption of the CDS tool (Aim 3).

<u>Quantitative data</u>: Study data will be audited to detect outliers and check variable distributions to ensure they meet analysis assumptions. We will look for between-arm differences in the study CHCs' baseline characteristics for all elements in Table 5, via chi-square, t-test, and nonparametric Wilcoxon rank-sum tests, to verify that randomization yielded similar groups; analyses will be adjusted for any differences. Analysis datasets will be hierarchically structured as encounters within patients within CHCs. To be included in Aim 1 analysis a patient must have (a) an index visit in the 6 months after the Arm 1 implementation date, at an Arm 1 or Arm 2 CHC, and (b) ≥2 CHC visits in the 12-month post-index visit period. We will describe those who had inadequate follow-up data. For each study subject in Aim 1 analyses, CVD risk score / risk factor management status will be calculated at index visit and each encounter during follow-up. Since clinics' ability to implement CDS is influenced by diverse contextual factors, our analyses will consider the impact of factors expected to affect acceptance and uptake of CV Wizard, specified based on our intervention logic model (Table 3) and past research from our team's implementation scientists and others on uptake of EHR tools in CHCs.

CV Wizard can accept data from any EHR; the study CHCs use Epic, the same EHR as at HealthPartners. Working with HealthPartners programmers who previously disseminated CV Wizard to other large care systems, OCHIN programmers will (1) build tables for data extraction (e.g., medication, laboratory, diagnostic / problem list codes, vitals, smoking); (2) develop the interconnect routines needed to transmit EHR data to the CV Wizard web service, save response information in EHR flow sheets, and display the CDS results in the EHR; (3) create the alert to prompt CDS use in targeted patients; (4) develop 'smart tools' (e.g., dot phrases) to facilitate documenting CV Wizard use and results in encounter notes; (5) program the dynamic order sets that are tailored to facilitate clinical actions recommended for each patient; (6) create CDS use rate feedback reports; and (7) develop a process to manage provider feedback on clinical aspects of CV Wizard. The programmers involved in this work have extensive experience in conducting such tasks, and have verified the feasibility of these steps.

We will conduct intent to treat (ITT) analyses (primary) to compare targeted patients in Arm 1 vs. Arm 2 CHCs in months 13-30, and effect of treatment on the treated (ETOT) (secondary) analyses to compare

CVD treatment outcomes among Arm 1 CHC patients for whom the CV Wizard risk assessment was used at the index visit versus those targeted patients for whom it was not used.

Statistical power: To conservatively calculate power for our main outcome (CVD risk score) we estimated 60 clinics with >35 high-CVD risk patients, randomized to two arms; then, using means and standard deviations for this measure from previous studies, we varied effect size associated with the group x time interaction and intraclass correlation. We examined a 1.5%, 2%, 3% greater absolute reduction in risk score over time in intervention vs. control CHCs; Table 7. Aggressive management can reduce 10-year CVD risk by an absolute 4-5% in high-risk patients in a short time. We do not expect so large an effect, and have power to detect smaller effects.

Qualitative data: Using a realist approach our process evaluation will explore 'what works, for whom and in what circumstances' related to uptake / impact of the CDS system. It will be guided by our logic model and the TAM's conceptualization of the relationship between user perception and CDS acceptance / use. We will identify mechanisms of change (barriers / facilitators; impact of implementation strategy) at the clinician and health care system levels, and assess how contextual factors impact outcomes.

We will triangulate process data from all sources for a deep understanding of CV Wizard's acceptance / use in the CHCs. We will look at areas of consistency / inconsistency in these data (where different data sources show the same thing, or do not). A grounded theory approach coupled with an immersion-crystallization process will be used to identify themes and patterns in the qualitative data. We will emphasize factors influencing use of CV Wizard, and its impact on care decisions. Data collection and analyses will be parallel and iterative, letting us identify salient constructs and knowledge gaps while implementation is ongoing. The study team will meet regularly to discuss and integrate qualitative and quantitative process evaluation and outcome data to help us identify factors that affect success. The integration of mixed methods data will provide a more complete, nuanced understanding of the impact and use of the CV Wizard system than either method alone, and permit examination of the reliability and validity of the data sources.

b. Sharing of Results with Subjects

When / if the CV Wizard decision support tool is used, to ensure patient confidentiality, the name of the patient is printed on the CDS sheet given to the patient and their care provider at the clinic visit.

This study does not carry the risk of incidental findings.

This study does not involve laboratory results.

c. Data and Specimen Banking

Not applicable. Please note that all data storage will be at OCHIN.

7. Privacy, Confidentiality, and Data Security

Quantitative data management, sharing and protection: Yearly datasets will be created with data from CV Wizard's web server, imported into SAS, and securely maintained at OCHIN. Data will be extracted for all target patients seen in study CHCs, and stored (limited data set) in a Web-based secure repository. Data from subsequent encounters is stored and linked to the same patient. OCHIN, CHR, and HealthPartners all have

expertise in high-quality data management, project operations, and multisite research collaborations. Data standards in place exceed Level 1 HIPAA requirements, and meet most Level 2 requirements. Data structures will remove personal identifiers. Analyses will be done in SAS® v9 or higher. Only aggregate EHR data will be shared outside of OCHIN.

Qualitative data management, sharing and protection: Qualitative data will be securely saved at CHR, in a database on a secure network; only de-identified data will be shared. Data will be catalogued within 1 week of collection, and field notes within 24 hours. Interviews will be recorded and professionally transcribed by an outside vendor (with who KPCHR has a Business Agreement with). Data will be entered into QSR NVivo; data files will be password protected; data sharing agreements / protections will be set as necessary; transfers will use a secure website.

Quantitative data: Data from the CHCs will come from OCHIN's EHR. OCHIN uses safeguards similar to those used at CHR to ensure confidentiality when handling data. All patient-related study data will be de-identified and unique patient identification codes will be used, and all data sources will be linked through a secure relational database at OCHIN. Data sent to CV Wizard, and then returned to OCHIN for analysis, will be de-identified and protected as described below. Aggregate data will be shared with CHR through a secure data transfer web site, as necessary. The CV Wizard CDS system is a web service that uses input EHR data to identify eligible patients, compute CV risks, prioritize CV risk reduction, and provide treatment recommendations based on a complex set of evidence-based algorithms. A web-based display of the results is then provided to the primary care providers.

Confidentiality measures: All CHR investigators and project staff, all HealthPartners data managers, and all OCHIN staff who handle data, sign annual confidentiality pledges and receive annual IRB and HIPAA training and certification.

Security measures: Multiple measures are in place to ensure security of PHI. Data transfer to and from the EHR (at OCHIN), the web service (at Health Partners), and the web display (accessed at OCHIN member CHCs) uses a Simple Object Access Protocol (SOAP) with Secure Sockets Layer (SSL) encryption over a Hypertext Transfer Protocol Secure (HTTPS) computer network. There is a double firewall in the Web service so that once the data flow through the initial Web service firewall, the data cross another firewall into a new secure pathway that once again employs SOAP, SSL, and HTTPS to process the data and provide recommendations. This includes sending the data through a batch server for more efficient processing, but all within the double-firewall Web service. Limited clinical data for all adults are initially extracted from the EHR to determine CDS eligibility.

We estimate that 15-25% may meet the eligibility criteria at which point more clinical information is extracted and full processing through the clinical algorithms occurs and is displayed back to the providers. When / if the CV Wizard decision support tool is used, to ensure patient confidentiality, the name of the patient is printed on the CDS sheet given to the patient and their care provider at the clinic visit. This is needed to avoid mixing up printed pages and giving protected health information (PHI) to the wrong patient. It is also desirable to have each patient's name on this sheet of paper to assure the patient that the information on the paper is related to their own health state and not someone else's. The technical aspects of this system are also relevant; the printout is controlled from the CDS Web site, so the Web site must have the patient's name and other PHI. With the analytic databases, measures will be taken to protect PCPs and patients from the risk of breach of confidentiality: A unique study ID code unrelated to the EHR record number or other study subject-specific information will be assigned to each patient and provider study subject and used to link data from various sources needed for analysis. A crosswalk table linking this code number to a provider PCP or patient name or medical record number will be destroyed within 12 months of completion of the linked databases needed for study analyses. To minimize the risk that a PCP will act wrongly on the basis of information provided through

CDS developed for this study, communication to providers will have a written explanation that the CDS is a suggestion, not a mandate, and that the action should only be taken if judged to be clinically appropriate by the treating provider on the basis of the patient's current clinical status and preferences.

8. Provisions to Monitor the Data to Ensure the Safety of Subjects

This is required when research involves more than Minimal Risk to subjects. Describe:

- *Who will monitor the study data for safety.*
- *Who will verify data accuracy and conduct quality assessments.*
- *How objectivity in the monitoring process will be ensured.*
- *What data and/or events will be reported to the monitor or monitoring board and how frequently.*
- *The procedures and methods that the monitor or board will use to evaluate the data.*
- *Criteria for taking action on monitoring findings (for instance, stopping rules, reporting, protocol changes, changes to monitoring frequency or plan).*
- *For studies monitored by a DSMB/C, describe the committee membership and structure, meeting format, and quorum requirements. Upload the board/committee charter, if one exists.*

This study involves data from OCHIN, and through OCHIN from 60 safety net CHCs. The web service for CV WIZARD is hosted by Health Partners. All EHR data from OCHIN clinics are stored at OCHIN. Each clinic has business use agreements with OCHIN to handle and manage PHI from their clinical data. EHR data will be linked using OCHIN's unique patient identification codes, and data sources will be linked into a secure relational database at OCHIN. When data linkages have been completed, the data will be fully de-identified. Data analysis will be conducted at OCHIN. De-identified, aggregate data will be shared with study team members from CHR and HealthPartners.

If necessary to share PHI, we will use a secure data transfer website with access limited to appropriate members of the research team. IRB and HIPAA approval will be obtained for all study steps. CHR and OCHIN have expertise in high quality data management and project operations and multisite research collaborations. Data structures are typically designed to separate personal identifiers from other critical data, further enhancing protections. CHR, HealthPartners, and OCHIN standards meet or exceed requirements for patient data safety established in the federal HIPAA guidelines. Data structures will derive from the data confidentiality, security, and privacy standards that CHR /OCHIN/HealthPartners have in place to meet or exceed all current HIPAA requirements. These standards exceed Level 1 requirements, and meet the majority of Level 2 requirements, as specified by DHHS in its Automated Information Systems Security Handbook. All analyses will be carried out using SAS® version 9 or later. Quality control will begin with real-time, inter-field checks in the data at OCHIN. Additional back-end checks (for missing data and logical inconsistencies) will be conducted to ensure the highest standards of data reliability. We will examine the distribution and measurement properties of variables before making final decisions about analyses.

In addition, we will compose a data safety and monitoring board (DSMB) of three members with expertise in clinical trials methodology and the clinical domains addressed in the proposed research. Members will include one clinician with expertise in CV disease and treatment, one expert in information technology, and one statistical expert. A representative of the funding agency will be invited to participate via conference call at all formal DSMB meetings. The PI will participate in the DSMB meetings in a limited way, as recommended in NIH policy. The DSMB will provide input and guidance on the study evaluation and intervention protocols, including quality assurance and safety issues related to the protocols and intervention strategy, as well as data-handling activities. The DSMB will provide periodic input via email, conference calls, and annual meetings. The DSMB will

meet within 3 months of the beginning of the study and twice annually through the study. A special focus of interest will be the safety of patients exposed to the study intervention. The intervention provides point-of-care CDS related to management of elevated CV risk and suggests evidence-based treatment options based on national guidelines, and further vetted by clinical leaders at OCHIN and HealthPartners. CDS recommendations provided as part of the intervention are designed to support clinicians' decision-making, not to override clinical judgment. Adverse-events information will be collected by the Institute Project Manager and recorded on standard forms based on those used in other trials (HL102144). Consistent with NIH, CHR, and HealthPartners IRB policy, all adverse events will be promptly reported in writing to the NIH, the DSMB, the CHR IRB, and the HealthPartners IRB.

9. Risks and Benefits

a. Risks to Subjects

One risk to clinic patients is: breach of privacy and subsequent loss of confidentiality, but this is extremely unlikely. All CHR, HealthPartners, and OCHIN staff are highly trained and appropriately certified in data security and confidentiality, and the current protection measures represent the cutting edge of electronic protection. All patient data used in analyses will be de-identified by OCHIN staff, who work with patient data from their member clinics on a regular basis and are appropriately trained in data security. Thus, we feel any risk due to loss of confidentiality is remote.

The other potential risk is: the possibility that the intervention may provide advice on the basis of national evidence-based CVD guidelines, which may be inappropriate for a given individual patient and, if applied without further checking the clinical status of the patient, could lead to erroneous therapy, or adverse events. However, the recommendations are evidence-based and operationalize current national and regional standards of care and, therefore, the risk of untoward consequences of such clinical actions is considered minimal. Moreover, this potential risk is routinely present in every clinical encounter in the healthcare system. All treatment recommendations will be evidence-based, but acting on them will ultimately be up to the provider's judgement; thus, we do not anticipate any clinical harm to patients based on patients / their providers seeing the CV Wizard recommendations. We have described below the methods used to minimize this risk. In addition, our qualitative data collection will include a focus on potential harms to patients – such as anxiety – that may be incurred by their exposure to the CV Wizard CVD risk assessment.

b. Potential Benefits to Subjects

Overall benefits: This study is expected to yield several key benefits. It will determine whether and how clinical decision support (CDS) tools that address multiple aspects of guideline-based CVD prevention and care, have provider- and patient-facing elements (enabling patient engagement), and involve some workflow changes, can and will be adopted by, and will successfully increase rates of guideline-concordant care in CHCs. The use of sophisticated CDS in populations with persistent disparities is a long overdue, critical step towards addressing CVD risk / outcome disparities in socioeconomically vulnerable (i.e., CHC) populations in the US. This study will determine how persistent guideline-to-practice gaps impacting high-risk / prevalence patients can be addressed using targeted, innovative, multi-level, team-based decision support tools. Thus, key benefits include: 1) Collecting, documenting and presenting data on prioritized CVD risk to care teams and patients may result in patients receiving care that ultimately improves their health. This potential benefit will incur no additional clinic visits or costs to patients. 2) The intervention will increase care teams' knowledge about CVD risks that may influence their patients'

health. 3) This intervention may bring improvements in EHR functionality to CHCs that would otherwise not receive them. 4) Because this study develops and tests different ways of presenting CVD risk data in clinic EMRs, it will provide needed information on how prioritized CVD risk data can be used to improve care and services in community health center populations nationwide.

Findings will support further improvements in the US healthcare system to mitigate health disparities, and will inform future efforts to bring cutting-edge CDS to CHCs. If the intervention significantly improves primary care with respect to identification or management of elevated CV risk factors in adults, the risk of CVD events and/or mortality related to elevated CV risk may be reduced later in life for large numbers of patients. If the intervention fails to improve identification or management of CV risk factors, that knowledge will also be important because it will direct the attention of investigators to other potentially more fruitful lines of investigation. Thus, regardless of specific findings, the results of this trial will provide important new knowledge that may ultimately contribute to improved care for adults with elevated CV risk.

<u>Quantitative</u>: Patients at the study CHCs will have no defined personal benefit from this project.

<u>Qualitative:</u> Care team members at the study CHCs will have no defined benefits from participating in this project. However, the intervention is designed to optimize identification and management of adult subjects with elevated CV risk. Some providers exposed to this potentially useful CDS may use it to improve their clinical care during the study or after.

10. Costs to Participants

There are no new costs to participants as a result of this study; however they will continue to pay for co-pays, routine treatment costs, etc. as part of normal care at their CHC.

11. Compensation to Participants

Describe any compensation provided to participants, for example, for time inconvenience, discomfort, travel, or in the event of research related injury.

If applicable, describe how you will inform participants of this prior to their enrollment in the study, including if payment will be prorated if the subject withdraws early from the study.

Note: *payment may not be withheld as an incentive for participants to complete the study.*

We will recruit two clinics per arm for an in-depth case study. These four clinics will receive annual impact fees to compensate for staff time spent on this evaluation. Clinic impact fees are estimated at $1,000 per clinic per year and total $10,000 in Years 2-5.

Clinic staff who participate in qualitative phone interviews, and patients who participate in an in-person qualitative interview, will each receive a $25 gift card.

12. Resources Available

No special resources or expertise are required to conduct this study.

13. Drugs or Devices

Not applicable.

14. Multi-Site Coordination

CHR will act as the coordinating center for this study. We will ensure that:

- All sites have the most current version of the protocol, consent document, and HIPAA authorization.
- All required approvals have been obtained at each site (including approval by the site's IRB of record).
- All modifications have been communicated to sites, and approved (including approval by the site's IRB of record) before the modification is implemented.
- All engaged participating sites will safeguard data as required by local information security policies.
- All local site investigators conduct the study appropriately.
- All non-compliance with the study protocol or applicable requirements will be reported in accordance with local policy.
- Communication of problems, interim results, and study closure.

15. Community-Based Participatory Research

<u>This study has system-level support:</u> OCHIN's leadership strongly supports activating CV Wizard in its member CHCs, which aligns with OCHIN's ongoing efforts to improve care and outcomes in CHCs via EHR-based strategies. In all study steps, the study team will engage OCHIN's operational leadership (including co-I Karen Parr, a CHC clinician and informaticist). We will also engage OCHIN CHC clinicians via existing communication structures. OCHIN has a long history of engaging stakeholders in all system-wide efforts; CHC clinicians serve on standing committees that direct all changes made to OCHIN's EHR. These committees include: OCHIN's Executive Leadership Team, the Practice-based Research Network (PBRN) formed in 2006, the Clinical Operations Group, and the Clinical Review Advisory Committee. We received enthusiastic support from these groups for this proposal. In the proposed work, we will engage OCHIN leaders and clinicians by working directly with these groups to obtain their input and direction at key junctures.

Bibliography

1. Gilmer TP, O'Connor PJ, Sperl-Hillen JM, Rush WA, Johnson PE, Amundson GH, Asche SE, Ekstrom HL. Cost-effectiveness of an electronic medical record based clinical decision support system. Health Serv Res 2012 Dec;47(6):2137-58. PMCID:PMC3459233

2. Dudl RJ, Wang MC, Wong M, Bellows J. Preventing myocardial infarction and stroke with a simplified bundle of cardioprotective medications. Am J Manag Care 2009 Oct 1;15(10):e88-e94

3. Wong W, Jaffe M, Wong M, Dudl RJ. Implementation Study-Vohs National Quality Award. Community Implementation and Translation of Kaiser Permanente's Cardiovascular Disease Risk-Reduction Strategy. Perm J 2011;15(1):36-41

4. Feldstein AC, Perrin NA, Unitan R, Rosales AG, Nichols GA, Smith DH, Schneider J, Davino CM, Zhou YY, Lee NL. Effect of a patient panel-support tool on care delivery. Am J Manag Care 2010 Oct;16(10):e256-e266

5. O'Connor PJ, Sperl-Hillen JM, Rush WA, Johnson PE, Amundson GH, Asche SE, Ekstrom HL, Gilmer TP. Impact of electronic health record clinical decision support on diabetes care: a randomized trial. Ann Fam Med 2011 Jan;9(1):12-21. PMCID:PMC3022040

6. Ash JS, Sittig DF, Guappone KP, Dykstra RH, Richardson J, Wright A, Carpenter J, McMullen C, Shapiro M, Bunce A, et al. Recommended practices for computerized clinical decision support and knowledge management in community settings: a qualitative study. BMC Med Inform Decis Mak 2012;12:6. PMCID:PMC3334687

7. Ash JS, Sittig DF, Dykstra R, Wright A, McMullen C, Richardson J, Middleton B. Identifying best practices for clinical decision support and knowledge management in the field. Stud Health Technol Inform 2010;160(Pt 2):806-10

8. Bright TJ, Wong A, Dhurjati R, Bristow E, Bastian L, Coeytaux RR, Samsa G, Hasselblad V, Williams JW, Musty MD, et al. Effect of clinical decision-support systems: a systematic review. Ann Intern Med 2012 Jul 3;157(1):29-43

9. Kawamoto K, Houlihan CA, Balas EA, Lobach DF. Improving clinical practice using clinical decision support systems: a systematic review of trials to identify features critical to success. BMJ 2005 Apr 2;330(7494):765. PMCID:PMC555881

10. Lobach D, Sanders GD, Bright TJ, Wong A, Dhurjati R, Bristow E, Bastian L, Coeytaux R, Samsa G, Hasselblad V, et al. Enabling health care decisionmaking through clinical decision support and knowledge management. Evid Rep Technol Assess (Full Rep) 2012 Apr;(203):1-784

11. Souza NM, Sebaldt RJ, Mackay JA, Prorok JC, Weise-Kelly L, Navarro T, Wilczynski NL, Haynes RB. Computerized clinical decision support systems for primary preventive care: a decision-maker-researcher partnership systematic review of effects on process of care and patient outcomes. Implement Sci 2011;6:87. PMCID:PMC3173370

12. Roshanov PS, You JJ, Dhaliwal J, Koff D, Mackay JA, Weise-Kelly L, Navarro T, Wilczynski NL, Haynes RB. Can computerized clinical decision support systems improve practitioners' diagnostic test ordering behavior? A decision-maker-researcher partnership systematic review. Implement Sci 2011 Aug 3;6:88

13. Jaspers MW, Smeulers M, Vermeulen H, Peute LW. Effects of clinical decision-support systems on practitioner performance and patient outcomes: a synthesis of high-quality systematic review findings. J Am Med Inform Assoc 2011 May 1;18(3):327-34. PMCID:PMC3078663

14. Cleveringa FG, Gorter KJ, van den Donk M, van GJ, Rutten GE. Computerized decision support systems in primary care for type 2 diabetes patients only improve patients' outcomes when combined with feedback on performance and case management: a systematic review. Diabetes Technol Ther 2013 Feb;15(2):180-92

15. Moja L, Kwag KH, Lytras T, Bertizzolo L, Brandt L, Pecoraro V, Rigon G, Vaona A, Ruggiero F, Mangia M, et al. Effectiveness of computerized decision support systems linked to electronic health records: a systematic review and meta-analysis. Am J Public Health 2014 Dec;104(12):e12-e22. PMCID:PMC4232126

16. Murphy EV. Clinical decision support: effectiveness in improving quality processes and clinical outcomes and factors that may influence success. Yale J Biol Med 2014 Jun;87(2):187-97. PMCID:PMC4031792

17. Davis AM, Vinci LM, Okwuosa TM, Chase AR, Huang ES. Cardiovascular health disparities: a systematic review of health care interventions. Med Care Res Rev 2007 Oct;64(5 Suppl):29S-100S. PMCID:PMC2367222

18. Vazquez-Benitez G, Desai JR, Xu S, Goodrich GK, Schroeder EB, Nichols GA, Segal J, Butler MG, Karter AJ, Steiner JF, et al. Preventable major cardiovascular events associated with uncontrolled glucose, blood pressure, and lipids and active smoking in adults with diabetes with and without cardiovascular disease: a contemporary analysis. Diabetes Care 2015 May;38(5):905-12

19. Graham G. Disparities in cardiovascular disease risk in the United States. Curr Cardiol Rev 2015;11(3):238-45. PMCID:PMC4558355

20. Mueller M, Purnell TS, Mensah GA, Cooper LA. Reducing racial and ethnic disparities in hypertension prevention and control: what will it take to translate research into practice and policy? Am J Hypertens 2015 Jun;28(6):699-716. PMCID:PMC4447820

21. Lewey J, Choudhry NK. The current state of ethnic and racial disparities in cardiovascular care: lessons from the past and opportunities for the future. Curr Cardiol Rep 2014;16(10):530

22. Lillie-Blanton M, Evadne Rushing O, Ruiz S, Mayberry R, Boone L. Racial/Ethnic Differences in Cardiac Care: The Weight of the Evidence 2002 [cited 2015 Sep 30] Available from http://kff.org/disparities-policy/fact-sheet/racialethnic-differences-in-cardiac-care-the-weight/.

23. Spranger CB, Ries AJ, Berge CA, Radford NB, Victor RG. Identifying gaps between guidelines and clinical practice in the evaluation and treatment of patients with hypertension. Am J Med 2004 Jul 1;117(1):14-8

24. Parchman ML, Pugh JA, Romero RL, Bowers K. Competing demands or clinical inertia: the case of elevated glycosylated hemoglobin. Annals of Family Medicine 2007;5(3):196-201

25. Yawn B, Goodwin MA, Zyzanski SJ, Stange KC. Time use during acute and chronic illness visits to a family physician. Fam Pract 2003 Aug;20(4):474-7

26. Desai JR, Vazquez-Benitez G, Xu Z, Schroeder EB, Karter AJ, Steiner JF, Nichols GA, Reynolds K, Xu S, Newton K, et al. Who Must We Target Now to Minimize Future Cardiovascular Events and Total Mortality? Lessons From the SUPREME-DM Cohort Study. Circ Cardiovasc Qual Outcomes 2015 Aug 25;

27. Yoon PW, Tong X, Schmidt SM, Matson-Koffman D. Clinical preventive services for patients at risk for cardiovascular disease, National Ambulatory Medical Care Survey, 2005-2006. Prev Chronic Dis 2011 Mar;8(2):A43. PMCID:PMC3073436

28. Dimitropoulos L. Health IT Research Priorities To Support the Health Care Delivery System of the Future. (Prepared for the Agency for Healthcare Research and Quality under Contract No. 290-2009-00023-I.). Rockville, MD: Agency for Healthcare Research and Quality; 2014. Report No.: AHRQ Publication No. 14-0072-EF.

29. U.S.Government Accountability Office. Comparative Effectiveness Research: HHS Needs to Strengthen Dissemination and Data-Capacity-Building Efforts 2015 [cited 2015 Mar 3] Available from http://www.gao.gov/assets/670/668804.pdf.

30. Agency for Healthcare Research and Quality. The National Quality Strategy (NQS) 2015 [cited 2015 Sep 17].

31. A Ten Year Vision to Achieve Interoperable Health IT Infrastructure. Connecting Health and Care for the Nation: A 10-Year Vision to Achieve an Interoperable Health IT Infrastructure 2015 Available from http://www.healthit.gov/sites/default/files/ONC10yearInteroperabilityConceptPaper.pdf.

32. Care Coordination 2015 Available from http://www.ahrq.gov/professionals/prevention-chronic-care/improve/coordination/index.html.

33. Davis F. Perceived usefulness, perceived ease of use, and user acceptance of information technology. MIS Quarterly 1989;13(3):319-40

34. Davis FD, Bagozzi RP, Warshaw PR. User acceptance of computer technology: a comparison of two theoretical models. Management Science 1989;35(8):982-1003

35. Venkatesh V, Davis FD. A model of the antecedents of perceived ease of use: development and test. Decision Sciences 1996 Sep 1;27(3):451-81

36. Venkatesh V, Davis F. A theoretical extension of the technology acceptance model: four longitudinal field studies. Management Science 2000;46(2):186-204

37. Venkatesh V, Morris MG, Davis GB, Davis FD. User acceptance of information technology: Toward a unified view. User acceptance of information technology: Toward a unified view 2003;27:425-78

38. Holden RJ, Karsh BT. The technology acceptance model: its past and its future in health care. J Biomed Inform 2010 Feb;43(1):159-72. PMCID:PMC2814963

39. Witteman HO, Dansokho SC, Colquhoun H, Coulter A, Dugas M, Fagerlin A, Giguere AM, Glouberman S, Haslett L, Hoffman A, et al. User-centered design and the development of patient decision aids: protocol for a systematic review. Syst Rev 2015;4:11. PMCID:PMC4328638

40. Beasley JW, Wetterneck TB, Temte J, Lapin JA, Smith P, Rivera-Rodriguez AJ, Karsh BT. Information chaos in primary care: implications for physician performance and patient safety. J Am Board Fam Med 2011 Nov;24(6):745-51. PMCID:PMC3286113

41. Karsh BT, Holden RJ, Alper SJ, Or CK. A human factors engineering paradigm for patient safety: designing to support the performance of the healthcare professional. Qual Saf Health Care 2006 Dec;15 Suppl 1:i59-i65. PMCID:PMC2464866

42. Wickens CD. Multiple resources and mental workload. Hum Factors 2008 Jun;50(3):449-55

43. Altmann EM, Gray WD. Forgetting to remember: the functional relationship of decay and interference. Psychol Sci 2002 Jan;13(1):27-33

44. Committee on Patient Safety and Health Information Technology. Health IT and Patient Safety: Building Safer Systems for Better Care. Institute of Medicine 2011 Nov 10;

45. Computational Technology for Effective Health Care:Immediate Steps and Strategic Directions. Washington, DC: The National Academies Press; 2009.

46. Bailey SR, O'Malley JP, Gold R, Heintzman J, Marino M, DeVoe JE. Receipt of diabetes preventive services differs by insurance status at visit. Am J Prev Med 2015 Feb;48(2):229-33. PMCID:PMC4301980

47. Gold R, DeVoe J, Shah A, Chauvie S. Insurance continuity and receipt of diabetes preventive care in a network of federally qualified health centers. Med Care 2009 Apr;47(4):431-9. PMCID:PMC2730766

48. Gold R, DeVoe JE, McIntire PJ, Puro JE, Chauvie SL, Shah AR. Receipt of diabetes preventive care among safety net patients associated with differing levels of insurance coverage. J Am Board Fam Med 2012 Jan;25(1):42-9. PMCID:PMC3305239

49. Richard P, Alexandre PK, Lara A, Akamigbo AB. Racial and ethnic disparities in the quality of diabetes care in a nationally representative sample. Prev Chronic Dis 2011 Nov;8(6):A142. PMCID:PMC3221581

50. Jean-Jacques M, Persell SD, Thompson JA, Hasnain-Wynia R, Baker DW. Changes in disparities following the implementation of a health information technology-supported quality improvement initiative. J Gen Intern Med 2012 Jan;27(1):71-7

51. Goud R, de Keizer NF, ter RG, Wyatt JC, Hasman A, Hellemans IM, Peek N. Effect of guideline based computerised decision support on decision making of multidisciplinary teams: cluster randomised trial in cardiac rehabilitation. BMJ 2009;338:b1440. PMCID:PMC2674147

52. Lopez L, Green AR, Tan-McGrory A, King R, Betancourt JR. Bridging the digital divide in health care: the role of health information technology in addressing racial and ethnic disparities. Jt Comm J Qual Patient Saf 2011 Oct;37(10):437-45

53. Jaffe MG, Lee GA, Young JD, Sidney S, Go AS. Improved blood pressure control associated with a large-scale hypertension program. JAMA 2013 Aug 21;310(7):699-705. PMCID:PMC4270203

54. Shaw KM, Handler J, Wall HK, Kanter MH. Improving blood pressure control in a large multiethnic California population through changes in health care delivery, 2004-2012. Prev Chronic Dis 2014;11:E191. PMCID:PMC4215570

55. Comparative Effectiveness Research: HHS Needs to Strengthen Dissemination and Data-Capacity-Building Efforts, GAO-15-280 2015 Mar 5 [cited 2015 Aug 21] Available from http://www.gao.gov/products/GAO-15-280.

56. Office of the National Coordinator for Health Information Technology. Health IT Enabled Quality Improvement: A Vision to Achieve Better Health and Health Care 2014 [cited 2015 Sep 28] Available from https://www.healthit.gov/sites/default/files/HITEnabledQualityImprovement-111214.pdf.

57. Centers for Medicare and Medicaid Services. CMS Quality Strategy 2013 - Beyond 2013 [cited 2013 Nov 18] Available from https://www.cms.gov/medicare/quality-initiatives-patient-assessment-instruments/qualityinitiativesgeninfo/downloads/cms-quality-strategy.pdf.

58. Gold R, Nelson C, Cowburn S, Bunce A, Hollombe C, Davis J, Muench J, Hill C, Mital M, Puro J, et al. Feasibility and impact of implementing a private care system's diabetes quality improvement intervention in the safety net: a cluster-randomized trial. Implement Sci 2015;10(1):83. PMCID:PMC4461907

59. Stone NJ, Robinson J, Lichtenstein AH, Merz CNB, Blum CB, Eckel RH, Goldberg AC, Gordon D, Levy D, Lloyd-Jones DM, et al. 2013 ACC/AHA Guideline on the Treatment of Blood Cholesterol to Reduce Atherosclerotic Cardiovascular Risk in Adults: A Report of the American College of Cardiology/American Heart Association Task Force on Practice Guidelines. Circulation 2013 Nov 12;

60. Eckel RH, Jakicic JM, Ard JD, de Jesus JM, Houston MN, Hubbard VS, Lee IM, Lichtenstein AH, Loria CM, Millen BE, et al. 2013 AHA/ACC guideline on lifestyle management to reduce cardiovascular risk: a report of the American College of Cardiology/American Heart Association Task Force on Practice Guidelines. Circulation 2014 Jun 24;129(25 Suppl 2):S76-S99

61. Bibbins-Domingo K. Aspirin Use for the Primary Prevention of Cardiovascular Disease and Colorectal Cancer: U.S. Preventive Services Task Force Recommendation Statement. Ann Intern Med 2016 Apr 12;

62. Standards of Medical Care in Diabetes-2016: Summary of Revisions. Diabetes Care 2016 Jan;39 Suppl 1:S4-S5

63. Persell SD, Dolan NC, Baker DW. Medical exceptions to decision support: a tool to identify provider misconceptions and direct academic detailing. AMIA Annu Symp Proc 2008;1090

64. D'Agostino RB, Sr., Vasan RS, Pencina MJ, Wolf PA, Cobain M, Massaro JM, Kannel WB. General cardiovascular risk profile for use in primary care: the Framingham Heart Study. Circulation 2008 Feb 12;117(6):743-53

65. Pencina MJ, D'Agostino RB, Sr., Larson MG, Massaro JM, Vasan RS. Predicting the 30-year risk of cardiovascular disease: the framingham heart study. Circulation 2009 Jun 23;119(24):3078-84. PMCID:PMC2748236

66. Ram CV. Hypertension guidelines in need of guidance. J Clin Hypertens (Greenwich) 2014 Apr;16(4):251-4

67. Goff DC, Jr., Lloyd-Jones DM, Bennett G, Coady S, D'Agostino RB, Sr., Gibbons R, Greenland P, Lackland DT, Levy D, O'Donnell CJ, et al. 2013 ACC/AHA guideline on the assessment of cardiovascular risk: a report of the American College of Cardiology/American Heart Association Task Force on Practice Guidelines. J Am Coll Cardiol 2014 Jul 1;63(25 Pt B):2935-59

68. Hayes AJ, Leal J, Gray AM, Holman RR, Clarke PM. UKPDS outcomes model 2: a new version of a model to simulate lifetime health outcomes of patients with type 2 diabetes mellitus using data from the 30 year United Kingdom Prospective Diabetes Study: UKPDS 82. Diabetologia 2013 Sep;56(9):1925-33

69. Clarke PM, Gray AM, Briggs A, Farmer AJ, Fenn P, Stevens RJ, Matthews DR, Stratton IM, Holman RR. A model to estimate the lifetime health outcomes of patients with type 2 diabetes: the United Kingdom Prospective Diabetes Study (UKPDS) Outcomes Model (UKPDS no. 68). Diabetologia 2004 Oct;47(10):1747-59

70. Diabetes Care. Vol. 38, 2015.

71. James PA, Oparil S, Carter BL, Cushman WC, Dennison-Himmelfarb C, Handler J, Lackland DT, LeFevre ML, MacKenzie TD, Ogedegbe O, et al. 2014 evidence-based guideline for the management of high blood pressure in adults: report from the panel members appointed to the Eighth Joint National Committee (JNC 8). JAMA 2014 Feb 5;311(5):507-20

72. Third Report of the National Cholesterol Education Program (NCEP) Expert Panel on Detection, Evaluation, and Treatment of High Blood Cholesterol in Adults (Adult Treatment Panel III) final report. Circulation 2002 Dec 17;106(25):3143-421

73. Stone NJ, Robinson JG, Lichtenstein AH, Bairey Merz CN, Blum CB, Eckel RH, Goldberg AC, Gordon D, Levy D, Lloyd-Jones DM, et al. 2013 ACC/AHA guideline on the treatment of blood cholesterol to reduce atherosclerotic cardiovascular risk in adults: a report of the American College of Cardiology/American Heart Association Task Force on Practice Guidelines. Circulation 2014 Jun 24;129(25 Suppl 2):S1-45

74. Chobanian AV, Bakris GL, Black HR, Cushman WC, Green LA, Izzo JL, Jr., Jones DW, Materson BJ, Oparil S, Wright JT, Jr., et al. The Seventh Report of the Joint National Committee on Prevention, Detection, Evaluation, and Treatment of High Blood Pressure: the JNC 7 report. JAMA 2003 May 21;289(19):2560-72

75. Jensen MD, Ryan DH, Apovian CM, Ard JD, Comuzzie AG, Donato KA, Hu FB, Hubbard VS, Jakicic JM, Kushner RF, et al. 2013 AHA/ACC/TOS guideline for the management of overweight and obesity in adults: a report of the American College of Cardiology/American Heart Association Task Force on Practice Guidelines and The Obesity Society. Circulation 2014 Jun 24;129(25 Suppl 2):S102-S138

76. Redmon B, Caccamo D, Flavin P, Michels R, O'Connor P, Roberts J, Smith S, Sperl-Hillen J. Diagnosis and management of type 2 diabetes mellitus in adults. Bloomington (MN): Institute for Clinical Systems Improvement (ICSI); 2014 Jul.

77. Sherifali D, Nerenberg K, Pullenayegum E, Cheng JE, Gerstein HC. The effect of oral antidiabetic agents on A1C levels: a systematic review and meta-analysis. Diabetes Care 2010 Aug;33(8):1859-64. PMCID:PMC2909079

78. Bennett WL, Wilson LM, Bolen S, Maruthur N, Singh S, Chatterjee R, Marinopoulos SS, Puhan MA, Ranasinghe P, Nicholson WK, et al. Oral Diabetes Medications for Adults With Type 2 Diabetes: An Update [Internet]. Rockville (MD): 2011 Mar. Report No.: Report No.: 11-EHC038-EF. Agency for Healthcare Research and Quality (US) p.

79. Wald DS, Law M, Morris JK, Bestwick JP, Wald NJ. Combination therapy versus monotherapy in reducing blood pressure: meta-analysis on 11,000 participants from 42 trials. Am J Med 2009 Mar;122(3):290-300

80. Law M, Wald N, Morris J. Lowering blood pressure to prevent myocardial infarction and stroke: a new preventive strategy. Health Technol Assess 2003;7(31):1-94

81. Law MR, Morris JK, Wald NJ. Use of blood pressure lowering drugs in the prevention of cardiovascular disease: meta-analysis of 147 randomised trials in the context of expectations from prospective epidemiological studies. BMJ 2009;338:b1665. PMCID:PMC2684577

82. Brown MJ. Heterogeneity of blood pressure response to therapy. Am J Hypertens 2010 Sep;23(9):926- 8

83. O'Connor PJ, Sperl-Hillen J, Johnson PE, Rush WA, Crain AL. Customized feedback to patients and providers failed to improve safety or quality of diabetes care: a randomized trial. Diabetes Care 2009 Jul;32(7):1158-63. PMCID:PMC2699722

84. Institute for Clinical Systems Improvement, Kottke T. Chronic Disease Risk Factors, Primary Prevention of (Guideline) 2008 [cited 2015 Oct 6] Available from http://www.manyfacesconference.org/conference2008/ICSI-Primary%20Prevention%20of%20Chronic%20Disease-sec.pdf.

85. Institute for Clinical Systems Improvement. Diabetes Mellitus in Adults, Type 2; Diagnosis and Management of (Guideline) 2014 [cited 2015 Oct 3] Available from http://www.icsi.org/guidelines__more/catalog_guidelines_and_more/catalog_guidelines/catalog_endocri ne_guidelines/diabetes/.

86. Powell LH, Calvin JE, Jr., Mendes de Leon CF, Richardson D, Grady KL, Flynn KJ, Rucker-Whitaker CS, Janssen I, Kravitz G, Eaton C. The Heart Failure Adherence and Retention Trial (HART): design and rationale. Am Heart J 2008 Sep;156(3):452-60. PMCID:PMC3609705

87. van der Wal MH, Jaarsma T. Adherence in heart failure in the elderly: problem and possible solutions. Int J Cardiol 2008 Apr 10;125(2):203-8

88. Flynn KJ, Powell LH, Mendes de Leon CF, Munoz R, Eaton CB, Downs DL, Silver MA, Calvin JE. Increasing self-management skills in heart failure patients: a pilot study. Congest Heart Fail 2005 Nov;11(6):297-302

89. Epstein RM, Franks P, Shields CG, Meldrum SC, Miller KN, Campbell TL, Fiscella K. Patient-centered communication and diagnostic testing. Ann Fam Med 2005 Sep;3(5):415-21. PMCID:PMC1466928

90. O'Connor AM, Llewellyn-Thomas HA, Flood AB. Modifying unwarranted variations in health care: shared decision making using patient decision aids. Health Aff (Millwood) 2004;Suppl Variation:VAR63-VAR72

91. O'Connor PJ, Sperl-Hillen JM, Crain AL, Elkstrom HL, argolis KL. Eliciting Patient Treatment Preferences Using a Clinical Decision Support System. Poster Presentation. In 2016 Jun 10; New Orleans, LA: American Diabetes Association's 76th Scientific Sessions; 2016.

92. Sperl-Hillen J, Crain L, et al. A Clinical Decision Support System promotes Shared Decision and CV Risk Factor Management. Forthcoming.

93. NACHC. The Safety Net on the Edge. 2008. 2010 [cited 2008 May 21] Available from http://www.nachc.com/client/documents/issues-advocacy/policy-library/researchdata/ research-reports/SNreport2005.pdf.

94. National Association of Community Health Centers. A Sketch Book of Community Health Centers: Chartbook 2014 2014 [cited 2015 Sep 28] Available from http://www.nachc.com/client/Chartbook_2014.pdf.

95. Kaiser Family Foundation. Patients Served by Federally-Funded Federally Qualified Health Centers 2015 [cited 2015 Oct 6] Available from http://kff.org/other/state-indicator/total-patients-served-by-fqhcs/.

96. Ash JS, Sittig DF, Wright A, McMullen C, Shapiro M, Bunce A, Middleton B. Clinical decision support in small community practice settings: a case study. J Am Med Inform Assoc 2011 Nov;18(6):879-82. PMCID:PMC3197983

97. Ash JS, Sittig DF, McMullen CK, McCormack JL, Wright A, Bunce A, Wasserman J, Mohan V, Cohen DJ, Shapiro M, et al. Studying the vendor perspective on clinical decision support. AMIA Annu Symp Proc 2011;2011:80-7. PMCID:PMC3243293

98. Eddy DM, Schlessinger L. Validation of the archimedes diabetes model. Diabetes Care 2003 Nov;26(11):3102-10

99. Eddy DM, Schlessinger L. Archimedes: a trial-validated model of diabetes. Diabetes Care 2003 Nov;26(11):3093-101

100. Glasgow RE, Lichtenstein E, Marcus AC. Why don't we see more translation of health promotion research to practice? Rethinking the efficacy-to-effectiveness transition. Am J Public Health 2003 Aug;93(8):1261-7

101. Glasgow RE, Goldstein MG, Ockene JK, Pronk NP. Translating what we have learned into practice. Principles and hypotheses for interventions addressing multiple behaviors in primary care. Am J Prev Med 2004 Aug;27(2 Suppl):88-101

102. Korda H. Bringing evidence-based interventions to the field: the fidelity challenge. J Public Health Manag Pract 2013 Jan;19(1):1-3

103. Scutchfield FD, Lamberth CD. Public health systems and services research: bridging the practice-research gap. Public Health Rep 2010 Sep;125(5):628-33. PMCID:PMC2924997

104. Gautam K. Addressing the research-practice gap in healthcare management. J Public Health Manag Pract 2008 Mar;14(2):155-9

105. Cohen DJ, Crabtree BF, Etz RS, Balasubramanian BA, Donahue KE, Leviton LC, Clark EC, Isaacson NF, Stange KC, Green LW. Fidelity versus flexibility: translating evidence-based research into practice. Am J Prev Med 2008 Nov;35(5 Suppl):S381-S389

106. Gold R, Muench J, Hill C, Turner A, Mital M, Milano C, Shah A, Nelson C, DeVoe JE, Nichols GA. Collaborative development of a randomized study to adapt a diabetes quality improvement initiative for federally qualified health centers. J Health Care Poor Underserved 2012 Aug;23(3 Suppl):236-46. PMCID:PMC3730843

107. O'Connor PJ, Crain AL, Rush WA, Sperl-Hillen JM, Gutenkauf JJ, Duncan JE. Impact of an electronic medical record on diabetes quality of care. Ann Fam Med 2005 Jul;3(4):300-6

108. O'Connor P. Opportunities to Increase the Effectiveness of EHR-Based Diabetes Clinical Decision Support. Appl Clin Inform 2011;2(3):350-4. PMCID:PMC3631926

109. O'Connor PJ, Desai JR, Butler JC, Kharbanda EO, Sperl-Hillen JM. Current status and future prospects for electronic point-of-care clinical decision support in diabetes care. Curr Diab Rep 2013 Apr;13(2):172-6. PMCID:PMC3595375

110. Gilmer TP, O'Connor PJ, Manning WG, Rush WA. The cost to health plans of poor glycemic control. Diabetes Care 1997;20(12):1847-53

111. Gilmer TP, O'Connor PJ, Sinaiko AR, Kharbanda EO, Magid DJ, Sherwood NE, Adams KF, Parker ED, Margolis KL. Impact of hypertension on healthcare costs among children. Am J Manag Care 2014 Aug;20(8):622-8. PMCID:PMC4430834

112. Angier H, Marino M, Sumic A, O'Malley J, Likumahuwa-Ackman S, Hoopes M, Nelson C, Gold R, Cohen D, Dickerson K, et al. Innovative Methods for Parents And Clinics to Create Tools for Kids' Care (IMPACCT Kids' Care) Study Protocol. Contemp Clin Trials 2015 Aug 17;

113. Bazemore AW, Cottrell EK, Gold R, Hughes LS, Phillips RL, Angier H, Burdick TE, Carrozza MA, DeVoe JE. "Community Vital Signs": Incorporating geocoded social determinants into electronic records to promote patient and population health. J Am Med Inform Assoc 2015 Jul 13;

114. DeVoe JE, Gold R, McIntire P, Puro J, Chauvie S, Gallia CA. Electronic health records vs Medicaid claims: completeness of diabetes preventive care data in community health centers. Ann Fam Med 2011 Jul;9(4):351-8. PMCID:PMC3133583

115. DeVoe JE, Angier H, Burdick T, Gold R. Health information technology: an untapped resource to help keep patients insured. Ann Fam Med 2014 Nov;12(6):568-72

116. Shi L, Tsai J, Higgins PC, Lebrun LA. Racial/ethnic and socioeconomic disparities in access to care and quality of care for US health center patients compared with non-health center patients. J Ambul Care Manage 2009 Oct;32(4):342-50

117. Blackwell DL, Lucas JW, Clarke TC. Summary health statistics for U.S. adults: National Health Interview Survey, 2012. National Center for Health Statistics. Vital Health Stat 2014;10(260)

118. Division for Heart Disease and Stroke Prevention. Cholesterol Fact Sheet 2015 [cited 2015 Sep 17] Available from http://www.cdc.gov/dhdsp/data_statistics/fact_sheets/docs/fs_cholesterol.pdf.

119. Ali MK, Bullard KM, Saaddine JB, Cowie CC, Imperatore G, Gregg EW. Achievement of goals in U.S. diabetes care, 1999-2010. N Engl J Med 2013 Apr 25;368(17):1613-24

120. Nwankwo T, Yoon SS, Burt V, Gu Q. Hypertension among adults in the United States: National Health and Nutrition Examination Survey, 2011-2012. NCHS data brief, no 133. Hyattsville, MD: National Center for Health Statistics; 2013.

121. DeVoe JE, Gold R, Cottrell E, Bauer V, Brickman A, Puro J, Nelson C, Mayer KH, Sears A, Burdick T, et al. The ADVANCE network: accelerating data value across a national community health center network. J Am Med Inform Assoc 2014 May 12;21(4):271-8

122. Steele GC, Khan AI, Kuluski K, McKillop I, Sharpe S, Bierman AS, Lyons RF, Cott C. Improving Patient Experience and Primary Care Quality for Patients With Complex Chronic Disease Using the Electronic Patient-Reported Outcomes Tool: Adopting Qualitative Methods Into a User-Centered Design Approach. JMIR Res Protoc 2016;5(1):e28. PMCID:PMC4777883

123. LeRouge C, Wickramasinghe N. A review of user-centered design for diabetes-related consumer health informatics technologies. J Diabetes Sci Technol 2013 Jul;7(4):1039-56. PMCID:PMC3879771

124. De Vito Dabbs A, Myers BA, Mc Curry KR, Dunbar-Jacob J, Hawkins RP, Begey A, Dew MA. User-centered design and interactive health technologies for patients. Comput Inform Nurs 2009 May;27(3):175-83. PMCID:PMC2818536

125. Henderson VA, Barr KL, An LC, Guajardo C, Newhouse W, Mase R, Heisler M. Community-based participatory research and user-centered design in a diabetes medication information and decision tool. Prog Community Health Partnersh 2013;7(2):171-84. PMCID:PMC4117400

126. Gold R, Hollombe C, Bunce A, Nelson C, Davis JV, Cowburn S, Perrin N, DeVoe J, Mossman N, Boles B, et al. Practices Enabling Implementation and Adaptation in the Safety Net (SPREAD-NET)': A pragmatic trial comparing implementation strategies. Implementation Science 2015;10:144. PMCID:PMC4609090

127. Gold R, Bunce AE, Cohen DJ, Hollombe C, Nelson CA, Proctor EK, Pope JA, DeVoe JE. Reporting on the strategies needed to implement proven interventions: An example from a "real-world" cross-setting implementation study. Mayo Clin Proc 2016 Apr 22;

128. Bailey SR, O'Malley JP, Gold R, Heintzman J, Likumahuwa S, DeVoe JE. Diabetes care quality is highly correlated with patient panel characteristics. J Am Board Fam Med 2013 Nov;26(6):669-79. PMCID:PMC3922763

129. Gold R, Kawachi I, Kennedy BP, Lynch JW, Connell FA. Ecological analysis of teen birth rates: association with community income and income inequality. Matern Child Health J 2001 Sep;5(3):161-7

130. Gold R, Connell FA, Heagerty P, Cummings P, Bezruchka S, Davis R, Cawthon ML. Predicting time to subsequent pregnancy. Matern Child Health J 2005 Sep;9(3):219-28

131. Gold R, Connell FA, Heagerty P, Bezruchka S, Davis R, Cawthon ML. Income inequality and pregnancy spacing. Soc Sci Med 2004 Sep;59(6):1117-26

132. Gold R, Kennedy B, Connell F, Kawachi I. Teen births, income inequality, and social capital: developing an understanding of the causal pathway. Health Place 2002 Jun;8(2):77-83

133. Michael YL, Nagel CL, Gold R, Hillier TA. Does change in the neighborhood environment prevent obesity in older women? Soc Sci Med 2014 Feb;102:129-37. PMCID:PMC3980662

134. Michael YL, Gold R, Perrin N, Hillier TA. Built environment and change in body mass index in older women. Health Place 2013 Jul;22:7-10. PMCID:PMC3679308

135. Michael YL, Gold R, Perrin NA, Hillier TA. Built environment and lower extremity physical performance: prospective findings from the study of osteoporotic fractures in women. J Aging Health 2011 Dec;23(8):1246-62. PMCID:PMC3655537

136. Feldstein AC, Glasgow RE. A practical, robust implementation and sustainability model (PRISM) for integrating research findings into practice. Jt Comm J Qual Patient Saf 2008 Apr;34(4):228-43

137. Gaglio B, Glasgow R. Evaluation approaches for dissemination and implementation research. In: Brownson RCCGA, Proctor EK, editors. Dissemination and Implementation Research in Health. New York: Oxford; 2012.

138. Glasgow RE, Marcus AC, Bull SS, Wilson KM. Disseminating effective cancer screening interventions. Cancer 2004 Sep 1;101(5 Suppl):1239-50

139. Jilcott S, Ammerman A, Sommers J, Glasgow RE. Applying the RE-AIM framework to assess the public health impact of policy change. Ann Behav Med 2007 Oct;34(2):105-14

140. Tabak RG, Khoong EC, Chambers DA, Brownson RC. Bridging research and practice: models for dissemination and implementation research. Am J Prev Med 2012 Sep;43(3):337-50

141. Gold R, Hollombe C, Bunce A, Nelson C, Davis JV, Cowburn S, Perrin N, DeVoe J, Mossman N, Boles B, et al. Study protocol for "Study of Practices Enabling Implementation and Adaptation in the Safety Net (SPREAD-NET)": a pragmatic trial comparing implementation strategies. Implement Sci 2015;10:144. PMCID:PMC4609090

142. DeVoe JE, Hoopes M, Nelson CA, Cohen DJ, Sumic A, Hall J, Angier H, Marino M, O'Malley AJ, Baker L, et al. Impact of Electronic Health Record Tools on Children's Insurance Coverage: A Mixed Methods Study (Submitted May 2016). Pediatrics 2016;

143. Powell BJ, McMillen JC, Proctor EK, Carpenter CR, Griffey RT, Bunger AC, Glass JE, York JL. A compilation of strategies for implementing clinical innovations in health and mental health. Med Care Res Rev 2012 Apr;69(2):123-57

144. Powell BJ, Waltz TJ, Chinman MJ, Damschroder LJ, Smith JL, Matthieu MM, Proctor EK, Kirchner JE. A refined compilation of implementation strategies: results from the Expert Recommendations for Implementing Change (ERIC) project. Implement Sci 2015;10:21. PMCID:PMC4328074

145. Proctor EK, Powell BJ, McMillen JC. Implementation strategies: recommendations for specifying and reporting. Implement Sci 2013;8:139. PMCID:PMC3882890

146. Mendel P, Meredith LS, Schoenbaum M, Sherbourne CD, Wells KB. Interventions in organizational and community context: a framework for building evidence on dissemination and implementation in health services research. Adm Policy Ment Health 2008 Mar;35(1-2):21-37

147. Glasgow RE, Emmons KM. How can we increase translation of research into practice? Types of evidence needed. Annu Rev Public Health 2007;28:413-33.:413-33

148. Rubenstein LV, Pugh J. Strategies for promoting organizational and practice change by advancing implementation research. J Gen Intern Med 2006 Feb;21 Suppl 2:S58-64.:S58-S64

149. Godley SH, Garner BR, Smith JE, Meyers RJ, Godley MD. A large-scale dissemination and implementation model for evidence-based treatment and continuing care. Clin Psychol (New York) 2011 Mar;18(1):67-83. PMCID:PMC3086782

150. Greenhalgh T, Robert G, Macfarlane F, Bate P, Kyriakidou O. Diffusion of innovations in service organizations: systematic review and recommendations. Milbank Q 2004;82(4):581-629

151. Lanham HJ, Leykum LK, Taylor BS, McCannon CJ, Lindberg C, Lester RT. How complexity science can inform scale-up and spread in health care: Understanding the role of self-organization in variation across local contexts. Soc Sci Med 2012 Jul 4;

152. Katz DL, Murimi M, Gonzalez A, Njike V, Green LW. From controlled trial to community adoption: the multisite translational community trial. Am J Public Health 2011 Aug;101(8):e17-e27. PMCID:PMC3134505

153. Harris JR, Cheadle A, Hannon PA, Forehand M, Lichiello P, Mahoney E, Snyder S, Yarrow J. A framework for disseminating evidence-based health promotion practices. Prev Chronic Dis 2012;9:E22

154. Wandersman A, Duffy J, Flaspohler P, Noonan R, Lubell K, Stillman L, Blachman M, Dunville R, Saul J. Bridging the gap between prevention research and practice: the interactive systems framework for dissemination and implementation. Am J Community Psychol 2008 Jun;41(3-4):171-81

155. Glasgow RE, Vinson C, Chambers D, Khoury MJ, Kaplan RM, Hunter C. National Institutes of Health approaches to dissemination and implementation science: current and future directions. Am J Public Health 2012 Jul;102(7):1274-81

156. Simmons R, Fajans P, Ghiron L. Scaling up health service delivery: from pilot innovations to policies and programmes. Geneva: The World Health Organization. 2007 [cited 2013 Jan 9].

157. Nieva VF, Swift E, Fair S. AHRQ Health Care Innovations Exchange report on Scale up and Spread Activities 2011. AHRQ Report, 2011. Rockville, MD: AHRQ; 2011.

158. Dingfelder HE, Mandell DS. Bridging the research-to-practice gap in autism intervention: an application of diffusion of innovation theory. J Autism Dev Disord 2011 May;41(5):597-609. PMCID:PMC3077435

159. Cilenti D, Brownson RC, Umble K, Erwin PC, Summers R. Information-seeking behaviors and other factors contributing to successful implementation of evidence-based practices in local health departments. J Public Health Manag Pract 2012 Nov;18(6):571-6

160. US Department of Health & Human Services. U.S. Department of Health & Human Services Strategic Plan Fiscal Years 2010 - 2015 2010 [cited 2013 Jan 24].

161. Grimshaw JM, Shirran L, Thomas R, Mowatt G, Fraser C, Bero L, Grilli R, Harvey E, Oxman A, O'Brien MA. Changing provider behavior: an overview of systematic reviews of interventions. Med Care 2001 Aug;39(8 Suppl 2):II2-45

162. Forsetlund L, Bjorndal A, Rashidian A, Jamtvedt G, O'Brien MA, Wolf F, Davis D, Odgaard-Jensen J, Oxman AD. Continuing education meetings and workshops: effects on professional practice and health care outcomes. Cochrane Database Syst Rev 2009;(2):CD003030

163. Gilbody S, Whitty P, Grimshaw J, Thomas R. Educational and organizational interventions to improve the management of depression in primary care: a systematic review. JAMA 2003 Jun 18;289(23):3145- 51

164. Wensing M, Fluit C, Grol R. Educational strategies. In: Grol R, Wensing M, Eccles M, et al., editors. Improving Patient Care: The Implementation of Change in Health Care, 2nd Edition. Hoboken, NJ: Wiley-Blackwell; 2013. p. 197-209.

165. Davis D, Davis N, Johnson N. Formal educational interventions. In Knowledge Translation in Health Care. John Wiley & Sons, Ltd; 2013. p. 163-75.

166. Grol R, Grimshaw J. From best evidence to best practice: effective implementation of change in patients' care. Lancet 2003 Oct 11;362(9391):1225-30

167. Chaillet N, Dumont A. Evidence-based strategies for reducing cesarean section rates: a meta-analysis. Birth 2007 Mar;34(1):53-64

168. Ivers N, Jamtvedt G, Flottorp S, Young JM, Odgaard-Jensen J, French SD, O'Brien MA, Johansen M, Grimshaw J, Oxman AD. Audit and feedback: effects on professional practice and healthcare outcomes. Cochrane Database Syst Rev 2012;6:CD000259

169. Jamtvedt G, Young JM, Kristoffersen DT, Thomson O'Brien MA, Oxman AD. Audit and feedback: effects on professional practice and health care outcomes. Cochrane Database Syst Rev 2003;(3):CD000259

170. Khunpradit S, Tavender E, Lumbiganon P, Laopaiboon M, Wasiak J, Gruen RL. Non-clinical interventions for reducing unnecessary caesarean section. Cochrane Database Syst Rev 2011;(6):CD005528

171. Walsh J, McDonald KM, Shojania KG, Sundaram V, Nayak S, Davies S, Lewis R, Mechanic J, Sharp C, Henne M, et al. Closing the Quality Gap: A Critical Analysis of Quality Improvement Strategies (Vol. 3: Hypertension Care). 2005 Jan. Report No.: Report No.: 04-0051-3.

172. Bravata DM, Sundaram V, Lewis R, Gienger A, Gould MK, McDonald KM, Wise PH, Holty JEC, Hertz K, Paguntalan H, et al. Closing the Quality Gap: A Critical Analysis of Quality Improvement Strategies (Vol. 5: Asthma Care). Rockville (MD): Agency for Healthcare Research and Quality; 2007.

173. Hysong SJ. Meta-analysis: audit and feedback features impact effectiveness on care quality. Med Care 2009 Mar;47(3):356-63. PMCID:PMC4170834

174. Dixon-Woods M, Redwood S, Leslie M, Minion J, Martin GP, Coleman JJ. Improving quality and safety of care using "technovigilance": an ethnographic case study of secondary use of data from an electronic prescribing and decision support system. Milbank Q 2013 Sep;91(3):424-54. PMCID:PMC3790520

175. Ivers NM, Grimshaw JM, Jamtvedt G, Flottorp S, O'Brien MA, French SD, Young J, Odgaard-Jensen J. Growing literature, stagnant science? Systematic review, meta-regression and cumulative analysis of audit and feedback interventions in health care. J Gen Intern Med 2014 Nov;29(11):1534-41. PMCID:PMC4238192

176. Simon SR, Smith DH, Feldstein AC, Perrin N, Yang X, Zhou Y, Platt R, Soumerai SB. Computerized prescribing alerts and group academic detailing to reduce the use of potentially inappropriate medications in older people. J Am Geriatr Soc 2006 Jun;54(6):963-8

177. Van Hoof TJ, Harrison LG, Miller NE, Pappas MS, Fischer MA. Characteristics of Academic Detailing: Results of a Literature Review. Am Health Drug Benefits 2015 Nov;8(8):414-22. PMCID:PMC4684632

178. Soumerai SB, Avorn J. Principles of educational outreach ('academic detailing') to improve clinical decision making. JAMA 1990 Jan 26;263(4):549-56

179. Chhina HK, Bhole VM, Goldsmith C, Hall W, Kaczorowski J, Lacaille D. Effectiveness of academic detailing to optimize medication prescribing behaviour of family physicians. J Pharm Pharm Sci 2013;16(4):511-29

180. Yeh JS, Van Hoof TJ, Fischer MA. Key Features of Academic Detailing: Development of an Expert Consensus Using the Delphi Method. Am Health Drug Benefits 2016 Feb;9(1):42-50. PMCID:PMC4822978

181. Fleuren M, Wiefferink K, Paulussen T. Determinants of innovation within health care organizations: literature review and Delphi study. Int J Qual Health Care 2004 Apr;16(2):107-23

182. Solberg LI. Improving medical practice: a conceptual framework. Ann Fam Med 2007 May;5(3):251-6

183. Durlak JA, DuPre EP. Implementation matters: a review of research on the influence of implementation on program outcomes and the factors affecting implementation. Am J Community Psychol 2008 Jun;41(3-4):327-50

184. Massoud MR, Nielson GA, Nolan K, Schall MW, Sevin C. A Framework for Spread: From Local Improvements to System-Wide Change. IHI Innovation Series white paper. Cambridge, MA: Institute for Healthcare Improvement 2006 [cited 2013 Jan 8].

185. Chang ET, Rose DE, Yano EM, Wells KB, Metzger ME, Post EP, Lee ML, Rubenstein LV. Determinants of Readiness for Primary Care-Mental Health Integration (PC-MHI) in the VA Health Care System. J Gen Intern Med 2012 Oct 5

186. Gold R, Nichols G, Muench J, Hill C, Mital M, Dudl J, DeVoe J, Puro J, Nelson C. Implementing an Integrated Care Setting's Diabetes QI Initiative in Safety Net Clinics: A Practice-Based Randomized Trial. Oral presentation. In Bethesda, MD: 5th Annual NIH conference on the Science of Dissemination and Implementation; 2012.

187. Bunce AE, Gold R, Davis JV, McMullen CK, Jaworski V, Mercer M, Nelson C. Ethnographic process evaluation in primary care: explaining the complexity of implementation. BMC Health Serv Res 2014 Dec 5;14(1):607. PMCID:PMC4265455

188. Patel A, MacMahon S, Chalmers J, Neal B, Billot L, Woodward M, Marre M, Cooper M, Glasziou P, Grobbee D, et al. Intensive blood glucose control and vascular outcomes in patients with type 2 diabetes. N Engl J Med 2008 Jun 12;358(24):2560-72

189. Pyorala K, Pedersen TR, Kjekshus J, Faergeman O, Olsson AG, Thorgeirsson G. Cholesterol lowering with simvastatin improves prognosis of diabetic patients with coronary heart disease. A subgroup analysis of the Scandinavian Simvastatin Survival Study (4S) [see comments] [published erratum appears in Diabetes Care 1997 Jun;20(6):1048]. Diabetes Care 1997 Apr;20(4):614-20

190. Collins R, Armitage J, Parish S, Sleigh P, Peto R. MRC/BHF Heart Protection Study of cholesterol-lowering with simvastatin in 5963 people with diabetes: a randomised placebo-controlled trial. Lancet 2003 Jun 14;361(9374):2005-16

191. Patel A, MacMahon S, Chalmers J, Neal B, Billot L, Woodward M, Marre M, Cooper M, Glasziou P, Grobbee D, et al. Intensive blood glucose control and vascular outcomes in patients with type 2 diabetes. N Engl J Med 2008 Jun 12;358(24):2560-72

192. Bonell C, Fletcher A, Morton M, Lorenc T, Moore L. Realist randomised controlled trials: a new approach to evaluating complex public health interventions. Soc Sci Med 2012 Dec;75(12):2299-306

193. Pawson R, Tilley N. Realistic Evaluation. London: SAGE Publications; 1997.

194. Dalkin SM, Greenhalgh J, Jones D, Cunningham B, Lhussier M. What's in a mechanism? Development of a key concept in realist evaluation. Implement Sci 2015;10:49. PMCID:PMC4408605

195. Moore GF, Audrey S, Barker M, Bond L, Bonell C, Hardeman W, Moore L, O'Cathain A, Tinati T, Wight D, et al. Process evaluation of complex interventions: Medical Research Council guidance. BMJ 2015;350:h1258. PMCID:PMC4366184

196. Lincoln Y, Guba EG. Naturalistic inquiry. Newberry Park, CA: Sage; 1985.

197. LeCompte MD, Schensul JJ. Designing and conducting ethnographic research. Vol. 1. Lanham, MD: Rowman Altamira; 2010.

198. Patton MQ. The View from Evaluation. NAPA Bulletin 2005 May 1;24(1):31-40

199. Dixon-Woods M, Bosk C. Learning through observation: the role of ethnography in improving critical care. Curr Opin Crit Care 2010 Dec;16(6):639-42

200. Smith-Morris C, Lopez G, Ottomanelli L, Goetz L, Dixon-Lawson K. Ethnography, fidelity, and the evidence that anthropology adds: supplementing the fidelity process in a clinical trial of supported employment. Med Anthropol Q 2014 Jun;28(2):141-61

201. Nastasi BK, Berg MJ. Using ethnography to strengthen and evaluate intervention programs. In: Glasser R, editor. Using ethnographic data: Interventions, public programming and public policy. Walnut Creek, CA: AltaMira Press; 1999. p. 1-56.

202. Glaser BG, Strauss AL. The discovery of grounded theory: Strategies for qualitative research. New York: Aldine de Gruyter; 1999.

203. Strauss A, Corbin J. Basics of qualitative research: Techniques and procedures for developing grounded theory (2nd ed.). Thousand Oaks, CA: Sage; 1998.

204. Charmaz K. Grounded Theory Methods in Social Justice Research. In: Denzin NK, Lincoln YS, editors. The SAGE Handbook of Qualitative Research, 4th ed. Thousand Oaks, CA: Sage Publications, Inc.; 2011. p. 359-80.

205. Borkan J. Immersion/crystallization. In: Crabtree BF, Miller WL, editors. Doing qualitative research. 2nd ed ed. Thousand Oaks, CA: Sage Publications, Inc.; 1999. p. 179-94.

206. Bradley EH, Curry LA, Devers KJ. Qualitative data analysis for health services research: developing taxonomy, themes, and theory. Health Serv Res 2007 Aug;42(4):1758-72. PMCID:PMC1955280

207. Aarons GA, Fettes DL, Sommerfeld DH, Palinkas LA. Mixed methods for implementation research: application to evidence-based practice implementation and staff turnover in community-based organizations providing child welfare services. Child Maltreat 2012 Feb;17(1):67-79

208. Teddlie C, Tashakkori A. Mixed Methods Research: Contemporary Issues in an Emerging Field. In: Denzin N, Lincoln Y, editors. The SAGE Handbook of Qualitative Research, 4th ed. 2011. p. 285-99.

209. Miller WL, Crabtree BF, Harrison MI, Fennell ML. Integrating mixed methods in health services and delivery system research. Health Serv Res 2013 Dec;48(6 Pt 2):2125-33. PMCID:PMC4097838

210. Jick TD. Mixing Qualitative and Quantitative Methods: Triangulation in Action. Administrative Science Quarterly 1979 Dec 1;24(4):602-11

211. Angier H, Gold R, Crawford C, O'M P, Tillotson J, Marino M, DeVoe JE. Linkage methods for connecting children with parents in electronic health record and state public health insurance data. Matern Child Health J 2014 Nov;18(9):2025-33

212. Angier H, Hoopes M, Gold R, Bailey SR, Cottrell EK, Heintzman J, Marino M, DeVoe JE. An early look at rates of uninsured safety net clinic visits after the affordable care act. Ann Fam Med 2015 Jan;13(1):10-6. PMCID:PMC4291259

213. Bailey S, Gold R, Heintzman J, Marino M, DeVoe J. Receipt of diabetes preventive services at a visit when services are due: Does insurance status matter? Medical Care 2013;(in press)

214. DeVoe JE, Marino M, Gold R, Hoopes MJ, Cowburn S, O'Malley JP, Heintzman J, Gallia C, McConnell KJ, Nelson CA, et al. Community Health Center Use After Oregon's Randomized Medicaid Experiment. Ann Fam Med 2015 Jul;13(4):312-20. PMCID:PMC4508170

215. Gold R, Angier H, Mangione-Smith R, Gallia C, McIntire PJ, Cowburn S, Tillotson C, DeVoe JE. Feasibility of evaluating the CHIPRA care quality measures in electronic health record data. Pediatrics 2012 Jul;130(1):139-49. PMCID:PMC3382922

216. Beebe J. Rapid Assessment Process: An Introduction. Walnut Creek, CA: AltaMira Press; 2001.

CV WIZARD PROTOCOL – 05/27/2021

1. Protocol Title

CV Wizard: Does a Prioritized, Point-of-Care Clinical Decision Support Tool Improve Guideline-Based CVD Risk Factor Control in Safety Net Clinics?

2. Objectives

This project aims to reduce disparities in cardiovascular disease (CVD) risk factor control and in rates of heart attacks and strokes among the low-income, racially/ethnically diverse Americans who receive primary care at safety net community health centers (CHCs). To achieve this objective, we will implement a successful clinical decision support (CDS) system—CV WIZARD (currently used in CVD care at several large, integrated health care systems)—in at least 60 CHCs, with a staggered randomized implementation approach. We will modify CV WIZARD as needed and feasible to fit CHC preferences. We will determine if use of this CDS improves CVD care, reduces disparities in CVD care and outcomes, and increases patient engagement in CVD treatment choices, in CHCs.

Specific Aims

Aim 1. Conduct a clinic-randomized trial of the impact of an evidence-based point-of-care CDS system on (i) overall CVD risk scores, and (ii) control of individual CVD risk factors (blood pressure; HbA1c, lipid levels; smoking; body mass index), among high CVD risk adult CHC patients.

Aim 2. Develop and hone need-based implementation support protocols to help Arm 1 CHCs implement the CV Wizard CDS system into their standard workflows; assess whether use of the protocols developed for Arm 1 CHCs accelerates implementation and adoption of the CDS system in the Arm 2 CHCs. **Supplement Addendum:** Compare CVD risk management in high-risk patients at in-person vs. virtual care (VC) encounters, in the periods pre- and post-COVID's onset.

Aim 3. Conduct a mixed methods process evaluation, guided by the Technology Acceptance Model (TAM), to identify and address patient, provider, and delivery system barriers to uptake/impact of this CDS in CHCs. **Supplement Addendum:** In a subset of study clinics, explore how CV Wizard's individualized, prioritized CVD risk summary is used in VC to guide care decisions and engage patients in shared decision-making.

3. Background

Substantial progress in reducing cardiovascular disease (CVD) morbidity and mortality would be achieved if evidence-based guidelines for CVD risk factor control were implemented consistently in primary care settings. Electronic health record (EHR)-based clinical decision support (CDS) systems that identify uncontrolled CVD risk factors and provide individualized care recommendations improved rates of guideline-concordant CVD care in large, integrated healthcare settings, but little is known about how effective such CDS may be in safety net community health centers (CHCs). CHCs' socioeconomically vulnerable patients have far worse CVD risk factor control and higher rates of major CVD events than the general population. Implementing CDS that leads to improved CVD risk factor control in

CHCs could reduce national disparities in CVD outcomes, but CHCs rarely have the resources to develop sophisticated CDS, and very few currently have such systems for CVD care. The proposed study is designed to address this issue. **Supplement Addendum**: Many healthcare settings rapidly shifted to virtual care (VC; defined here as video or telephone clinical encounters) because of the COVID-19 pandemic. Little is known about whether or how the shift to VC is impacting CVD risk management in primary care. The potential detrimental impacts of this shift are particularly concerning in community health centers (CHCs), which serve vulnerable patients who face health disparities (e.g., higher rates of unmanaged CVD risk than in the general population). However, the impact of VC on disparities in CVD care quality and outcomes remains largely unexplored. In response, we adapted CV Wizard for use in the VC setting and will assess how the COVID-19-driven shift to VC impacts CVD risk management in CHC populations.

NOTE: The bibliography has been uploaded as a separate document in the eIRB.

4. Study Design

CV WIZARD is a clinical decision support (CDS) tool that provides point-of-care cardiovascular disease (CVD) care recommendations to the primary care team and the patient. CV Wizard identifies a patient's uncontrolled CVD risk factors, prioritizes those factors based on potential CVD risk reduction for that patient, and generates specific guideline-based treatment recommendations for each uncontrolled risk factor.

We will recruit at least 60 safety net community health centers (CHCs) and randomize them to implement CV Wizard in study year 2 (Arm 1), or 18 months later (Arm 2). Prior to full implementation of the CV Wizard in Arm 1, two CHC organizations will be engaged to pilot-test CV Wizard. The pilot sites will test the accuracy and usability of the tool in clinic workflows.

We will compare outcomes from Arm 1 (immediate implementation) and Arm 2 (delayed implementation), which will enable us to measure the intervention's impact on CVD risk factor control in CHCs. Arm 1 CHCs will receive implementation support to address any barriers to adoption/sustained use of the CDS tool that are identified through study activities. We will apply these learnings to improve adoption rates in Arm 2.

Through this design, we will (1) assess whether this CDS tool is effective in CHCs, (2) identify and address barriers to its effectiveness and adoption, and (3) fine-tune strategies to support implementation of CDS systems in CHCs. To meet these aims, we will conduct mixed methods analyses (described below). Our assessment includes interviews with CHC staff, providers, and patients to determine if use of CV WIZARD increases patient engagement in CVD treatment choices.

5. Study Population

a. Number of Subjects

NOTE: This study does *not* include any KP subjects.

Study Aim 1
Clinics – We will randomize at least 60 CHCs from within the OCHIN collaborative to either immediate (Arm 1) or delayed (Arm 2) CV Wizard tool implementation. While CV Wizard will be used with patients in those clinics, they are not considered study subjects.

Study Aim 2
Clinic Staff

- We will conduct semi-structured phone interviews with staff from 20 clinics (10 per study arm). We will interview at least one staff member from each of these clinics and may conduct follow-up "clarifying" interviews as needed with up to 5 additional staff per clinic. Up to 60 clinic staff members may be interviewed for this data collection component.
- We will ask selected clinics (may include main trial and pilot clinics) to send a survey to their providers. While we do not know how many providers will respond to the survey, it is possible we will receive up to 500 responses.

Study Aim 3
Case Study Clinics – We will recruit four clinics from those taking part in the study (two per study arm; may include main trial and pilot clinics) for additional qualitative data collection. For these case study clinics, we will conduct semi-structured interviews with:

- Patients whose most recent encounter involved CV Wizard. We will conduct up to 60 patient interviews in total (up to 30 per study arm).
- Staff (providers and non-providers) who use the CV Wizard tool with patients or are involved in implementation efforts. We will conduct up to 60 staff interviews in total (up to 30 per study arm).

Supplement Clinics – We will recruit 10 clinics from those taking part in the study (from both study arms; may include main trial and pilot clinics) to collect qualitative data on how CV Wizard's individualized, prioritized CVD risk summary is used in VC to guide care decisions and engage patients in shared decision-making.

- We will interview 40 providers for whom CV Wizard was recommended at ≥3 VC encounters in the period post-COVID's onset.
- We will interview 30 patients who had a VC encounter at which CV Wizard was used in the post-COVID period from these same clinics.

b. Inclusion and Exclusion Criteria

CV Wizard – Clinical Decision Support Tool (Study Aim 1)
NOTE: *Individual patients are not being enrolled* for this clinic-randomized study. The goal of the study is to examine the uptake and impact of implementing CV Wizard into regular care processes. In this clinic-randomized trial, the intervention is provided at the clinic level and targets clinic processes that are part of routine patient care. CV Wizard will automatically identify patients age 40-75 years with either (a) reversible risk of cardiovascular disease (CVD) >10%, (b) diabetes + ≥1 uncontrolled risk factor or high A1c, or (c) existing atherosclerotic cardiovascular disease (ASCVD) + ≥1 uncontrolled risk

factor for CVD events. However, clinics can *manually* run the CV Wizard tool for any patient aged 18 and older.

Interviews with Clinic Staff and Patients (Study Aims 2 & 3)

Inclusion Criteria:
- Aged 18 years and older
- Experience with CV Wizard tool

Exclusion Criteria:
- Non-English speaker

Provider Survey (Study Aims 2 & 3)

Inclusion Criteria:
- Aged 18 years and older

Exclusion Criteria:
- Non-English speaker

c. Vulnerable Populations

CV Wizard – Clinical Decision Support Tool (Study Aim 1)
While individual patients are not being recruited/enrolled in this study, the CV Wizard tool may be used with individual patients as part of their routine care. Below is a list of vulnerable population categories and whether they will be included/excluded with regard to use of the CV Wizard tool:
- Children: Excluded
- Pregnant Women: Excluded (when identified as having a current pregnancy diagnosis); may be incidentally included, but not in a focused or targeted manner
- Neonates: Excluded
- Prisoners: Excluded (this study does *not* include any clinics that serve the prison population)
- Decisionally Impaired Adults: May be incidentally included, but not in a focused or targeted manner—just as part of the overall qualified sample

NOTE: Children younger than 18 years will be excluded, as different clinical guidelines apply to children with CVD risk. The study will not preferentially recruit any vulnerable populations. However, the criteria listed above may result in the inclusion of pregnant women and/or decisionally impaired adults. It is important to systematically address CV risk in these populations, as these patients may be at risk for elevated CV risk and often have been underrepresented in previous research.

Interviews with Clinic Staff/Patients and Provider Survey (Study Aims 2 & 3)
Below is a list of vulnerable population categories and whether they will be included/excluded with regard to clinic staff/patient interviews and provider surveys:
- Children: Excluded
- Pregnant Women: May be incidentally included, but not in a focused or targeted manner—just as part of the overall qualified sample
- Neonates: Excluded
- Prisoners: Excluded

- Decisionally Impaired Adults: May be incidentally included, but not in a focused or targeted manner—just as part of the overall qualified sample.

d. **Setting**

1. **OCHIN, Inc.** – OCHIN, Inc. is a non-profit, community-based health center-controlled network. Its members (>480 CHCs, in 19 states) share characteristics of other CHCs, so results will be generalizable to many CHCs. As the nation's largest CHC network with a single EHR system, OCHIN pioneered the development of EHR tools for CHCs. OCHIN's member CHCs share a single Epic© EHR, which is unduplicated, centrally maintained, and network-wide. Data are standardized and quality-checked: thus, validated data are already linked between all study sites.

   Procedures Performed
   - Recruit member clinics to take part in the study
   - Randomize clinics to intervention arm
   - Complete necessary programming to enable CV Wizard tool to accept OCHIN data
   - Perform validity testing to ensure CV Wizard works correctly with OCHIN's EHR data
   - Engage OCHIN's Patient Engagement Panel to obtain patient feedback on CV Wizard
   - Develop implementation training materials
   - Provide implementation support to participating clinics
   - Assist in the collection of qualitative data (e.g., webinar recordings, issues log, provider survey administration, etc.)
   - Conduct quantitative data analysis
   - Ensure all required MOUs for participating clinic have been fully executed; also ensure that modified/amended MOUs have been fully executed before the modification is implemented

   IRB Review Requirements – OCHIN will rely on the Kaiser Permanente Northwest IRB for all IRB review.

2. **HealthPartners Institute (HPI)** – HPI is one of the largest medical research and education centers in the Midwest. As part of HealthPartners integrated care system, the Institute uses research and education to accelerate improvements in quality, experience and affordability for members, patients, and the community. HPI owns the CV Wizard tool that will be used in this study.

   Study Procedures Performed
   - Work with OCHIN programmers to ensure CV Wizard can accept OCHIN data
   - Receive data from OCHIN through a secure file transfer to calculate the inputs necessary to populate the CV Wizard output.
   - Send back output data so CV Wizard recommendations can be displayed in OCHIN's EHR for clinical use at participating clinics.
   - Send output data file to OCHIN via secure file transfer that can be used for analyses and monitoring of CV Wizard use in the participating study clinics.
   - Updating CV Wizard tool as needed/requested by OCHIN and study clinics

IRB Review Requirements – HPI will rely on the Kaiser Permanente Northwest IRB for all IRB review.

3. **Center for Health Research—Northwest** – The Center for Health Research–Northwest (CHR-NW) is an academic-model organization that conducts independent research in a wide variety of areas including health services, public health, behavioral health, obesity and weight loss, mental health, maternal health, cost-effectiveness, cancer screening, genetics and genomics, and many others. In addition to conducting studies with our own databases and with KP's EMRs, CHR-NW researchers participate in a variety of formal and informal research networks. We collaborate locally with scientists and physicians at Oregon Health & Science University, and nationally with other Kaiser Permanente health plans and research divisions. We also undertake research in partnership with the Portland, Oregon-based OCHIN network of community health centers, and through our participation in networks such as HCSRN, CHARN, CRN, PCORnet, and many others. These networks allow our investigators to share data across multiple health systems and engage in multi-site research projects that access millions of individual health records.

   Study Procedures Performed
   - Provide coordination across study sites
   - Manage overall study budget and subcontract awards
   - Obtain IRB approval for study; submit continuing reviews; submit study modifications as needed
   - Prepare and submit all required reports to funder (e.g., recruitment reports; yearly progress reports)
   - Oversee DSMB creation and coordinate meetings
   - Conduct qualitative data analysis
   - Support subcontract sites with study tasks/deliverables as needed

   IRB Review Requirements – CHR-NW will rely on the Kaiser Permanente Northwest IRB for all IRB review.

e. **Recruitment Methods**

Clinic Recruitment (Aim 1)
OCHIN's Research Associate/Project Manager will work with KPCHR and the OCHIN research team to recruit CHCs. OCHIN's clinical leadership (including CEOs of its member CHC organizations) may also help with recruitment, as needed. Recruitment will be targeted to optimize diversity in clinic baseline characteristics.

OCHIN utilizes a variety of recruitment methods when they invite CHCs to participate in research studies. Recruitment activities for this study may include:
- Verbal conversations, in-person or by phone, with CHCs that the OCHIN research team has standing relationships with
- Sending an introductory recruitment email to allow clinics to opt-in to the study, with study-related attachments:
    - Video summarizing the CV Wizard tool use in a clinical setting
    - Slide deck with background information about the tool

    - One-page summary document with a high-level description of the tool and the study
- Presentation of recruitment materials at standing OCHIN webinars and meetings where members are in attendance. Any interested CHCs will be encouraged to contact study staff to discuss potential participation.

Clinic Staff Recruitment (Aim 2)
OCHIN and CHR's research teams, working by email and phone with clinic contacts identified during the recruitment process, will identity and recruit those staff members from clinics who have experience using CV Wizard, for phone interviews. The team will perform similar liaison and recruitment activities for any necessary follow-up interviews.

Case Study & Supplement Site Recruitment (Aim 3)

- **Clinics** – Once clinic-level recruitment from study aim 1 is complete, an OCHIN Research Associate/Project Manager will identify and engage clinics to be case study and/or supplement sites. A separate Memorandum of Understanding (MOU) outlining the participation requirements for this portion of the study will be executed for these sites. Each clinic will be asked to appoint a staff member to be the liaison between researchers and clinic staff.
- **Staff Interviews** – The KPCHR qualitative research team, supported by OCHIN study staff, will work with the clinic liaison to identify and recruit clinic staff to observe and/or interview for qualitative data collection activities. Interviews may take place either in-person (when site visits occur), or by telephone.
- **Patient Interviews** – Patients may be recruited by clinic staff or a member of the research team.
    - Recruitment by Clinic Staff: If CV Wizard is used during a patient encounter while research staff are onsite, clinic staff will inform the patient that a researcher would be interested in talking with them about their perceptions of the acceptability/utility of CV Wizard and related discussion with the provider. If CV Wizard is used during a patient encounter while research staff are *not* onsite, clinic staff may use a variety of methods to help recruit or identify patients for recruitment by the research team: (1) they may give the patient a flyer during an in-person visit; (2) they may send the flyer via post mail to a patient (in cases where the visit was virtual or the patient was identified after the visit); (3) they may indicate the patient is a good fit by (a) responding to a BPA alert in the EHR (a prompt that will fire when an eligible encounter is detected) or (b) using a smartphrase in the after visit summary (AVS) that indicates CV Wizard was used during the encounter; and/or (4) they may message the patient (e.g., via email or MyChart account) to share contact information for the research team (e.g., electronically sending the flyer, using the email message template provided by the research team).
    - Recruitment by Research Staff: If CV Wizard is used during a patient encounter that is being observed as part of a site visit, research staff may directly invite the patient to participate in the interview. If CV Wizard is used during a patient encounter when the research team is not onsite (e.g., virtual visits; post-covid qualitative data collection), research staff will rely on clinic staff recommendations (e.g., direct referral, positive responses to the BPA alert) and/or other indicators of CV Wizard use (e.g., smartphrase entry) to identify potential patients to recruit for the

interviews. Once identified, the OCHIN research team will use a variety of methods to follow up with patients (e.g., phone calls, email, text message, and/or post mail).

- NOTES:
  - The same recruitment flyer will be used for case study and supplement clinics.
  - Only OCHIN research team members will conduct the initial outreach to patients.
  - All recruitment materials will provide the patient with information on how to contact the CHR qualitative research team if the patient is interested in completing the interview by telephone. Patients calling the study phone number will be asked to leave a message, and research staff will return phone calls to complete recruitment and conduct the interview.

Provider Survey Recruitment (Aims 2 & 3)
The OCHIN team will email clinic contacts to introduce the provider survey. Clinic contacts will be asked to distribute the survey to the providers in their clinics via email, using the message template provided.

f. Consent Process

Clinic Consent (Aim 1)
Once a clinic agrees to participate, OCHIN's Research Associate/Project Manager will follow up with a Memorandum of Understanding (MOU) that outlines the study timeline and participation expectations. This MOU is signed by OCHIN's leadership (either the CEO or CFO) and leadership at the participating CHC. A separate MOU will be executed for the two (2) pilot sites that will be testing the tool prior to the go-live date for Arm 1 clinics.

Clinic Staff/Provider Consent (Aim 2)

- Clinic staff recruited for interviews will be provided with a study fact sheet (typically via email) that outlines the purpose of the research, study procedures, and contact information for the researchers and clinic liaison(s). As the risk to CHC staff is minimal, we will ask for their verbal consent for all qualitative data collection activities. We will also record interviews with staff permission; in these cases, we will document verbal consent as part of the audio recording.
- Clinic staff invited to complete the provider survey will be presented with text in the survey invitation that contains the necessary elements of consent. Because the risk to providers is minimal, we will infer consent if the survey is completed.

Case Study & Supplement Site Consent (Aim 3)

- **Clinics** – A separate MOU outlining the participation requirements for this portion of the study will be executed for case study and supplement sites.
- **Staff Interviews** – Clinic staff recruited for *in-person* interviews (at case study sites) will be provided with a study fact sheet that outlines the purpose of the research, study procedures, and contact information for the researchers and clinic liaison(s). They will be informed that participation in the interview is voluntary. Clinic staff recruited for *telephone interviews* (at case study and supplement clinic sites) will be read introductory text containing the required elements of consent (see provider/MA interview guides). As the risk to CHC staff is minimal, we will ask for their verbal consent

for all qualitative data collection activities. We will also record interviews with permission from staff. When permission is given to record interviews, verbal consent will be documented on the audio recording. *While PHI will not be solicited, it is possible that staff may inadvertently include PHI in their response to a question.*

- **Patient Interviews** – Patients recruited for in-person interviews (at case study sites) will be given the study fact sheet and will be informed that participation in the interview is voluntary. Patients recruited for telephone interviews (at case study and supplement sites) will be read introductory text containing the required elements of consent (see patient interview guide). Because risk is minimal, and some patients will be interviewed by telephone, we will ask the patient to provide verbal consent for participation. We will also record interviews with patient permission. When permission is given to record interviews, verbal consent will be documented on the audio recording. *Given that the interview will revolve around the patient's perceptions of the CV Wizard tool and use of the tool itself is capturing health information, it is possible that PHI will be included on the recording.*
- **Observations** – We will conduct observation of workflows, patient encounters, and clinic meetings relevant to CV Wizard during in-person case study site visits. Unlike the staff and patient interviews, these observations will not be audio recorded; instead, researchers will take field notes. When opportunities to observe patient encounters arise, the patient will be introduced to research staff and verbal consent will be obtained prior to the observation (note: the study fact sheet will not be presented during observation, as this has the potential to interfere with normal clinic workflow). *While PHI may be observed by research staff, it will not be written down as part of the field notes.*

Justification for Waiver of Documentation of Consent:

- Clinic Staff/Providers – The research involves minimal risk to clinic staff. All staff will be informed that participation in the interview is voluntary and may be discontinued at any time. In addition, the research does *not* include procedures for which written consent is normally required outside of the research context (e.g., quality improvement efforts).
- Patients – The research involves minimal risk to patients. While all patients invited to participate in an interview are, by definition, at increased risk of CVD, the research does *not* involve more than minimal risk. Patients will be informed that participation in the interview is voluntary. At any time, patients may choose to not answer specific questions or discontinue the interview, and all such requests will be immediately respected.

Justification for Waiver of HIPAA Authorization:

Patients – Interviews will include discussion of the patient's health information. While interviewers will not intentionally solicit PHI, it is possible that a patient might share this information during the semi-structured interview. While we could not practicably carry out this portion of the research (i.e., obtaining patient perceptions of the CV Wizard tool) without the possibility of collecting PHI, the PHI that might be collected is not the focus of our data collection efforts. Given that we will not be analyzing PHI, we will request that any identifiers be removed during the transcription process. The transcriptionist will be instructed to delete any identifiable patient information in the transcript and

replace with the type of information removed (e.g., [*Name*]) and to delete the recording once the transcription is complete. Only transcribed interviews will be shared with the research teams at OCHIN and/or HealthPartners.

6. Study Procedures

a. **Study Aim 1**

Clinic Identification, Recruitment & Assignment
Clinics will be included in the recruitment pool for this study if they (1) provide primary care to adults with high CVD risk annually, and (2) have been using OCHIN's EHR for at least 18 months. Once all clinics have been recruited as outlined in Section 5.e., they will be assigned to either arm 1 or arm 2. Clinics were grouped based on the baseline number of encounters each service area had, percent of patients with hypertension, and percent of patients who use tobacco. Five randomly generated groupings were created and the grouping that created the most even distribution across both Arms was then selected to assign service areas and the clinics within each service area to an Arm.

Programming CV Wizard to Accept OCHIN Data
Working with HealthPartners' programmers who previously disseminated CV Wizard to other large care systems, OCHIN's programmers will (1) build tables for data extraction (*e.g.,* medication, laboratory, diagnostic/problem list codes, vitals, smoking); (2) develop the interconnect routines needed to transmit EHR data to the CV Wizard web service, save response information in EHR flow sheets, and display the CDS results in the EHR; (3) create the alert to prompt CDS use in targeted patients; (4) develop "smart tools" (*e.g.*, dot phrases) to facilitate documenting CV Wizard use and results in EHR encounter notes; (5) program the dynamic order sets that are tailored to facilitate clinical actions recommended for each patient; (6) create CDS use rate feedback reports; and (7) develop a process to manage provider feedback on clinical aspects of CV Wizard.

Validity Testing
Since 2007, CV Wizard's algorithms have been continuously modified, and re-validated after each modification. OCHIN programmers will validate that CV Wizard works correctly with data sent from OCHIN's EHR as follows:

(1) OCHIN staff who routinely validate all changes made to the EHR will perform standard validation of data extracts for accuracy and completeness. This will include creating 'test patients' with variation in CVD risk factors and location of relevant data in the EHR, to assess how well CV Wizard performs with these patients. The validation team will also try to 'break' the CDS by testing how it performs with incomplete data. This will occur in a 'copy' of the Epic production environment used for testing EHR tools before they go into production.
(2) We will conduct preliminary pilot work with 2-3 CHC clinician advisors to assess how CV Wizard performs in real (not test) patient data. We will ask them to identify 20-30 of their high CVD risk patients, run those patients' data through CV Wizard's algorithms, and review CV Wizard's results for accuracy and clinical plausibility.
(3) Next, CV Wizard will be activated in pilot clinics served by clinician advisors, to further identify potential errors; these sites will be excluded from subsequent study

randomization.

(4) Further quality checks will occur throughout the study, via CV Wizard's feedback mechanism and built-in website monitoring routes designed to identify aberrant web service input or output. Any changes needed to the data sent to the CV Wizard website, as identified through these processes, will be reviewed by OCHIN / HealthPartners.

Patient Engagement

CV Wizard's patient-facing component is designed to support patient engagement in CVD risk management. To improve this aspect of the tool, we will engage OCHIN's Patient Engagement Panel (PEP). This panel is comprised of >20 patients from OCHIN member CHCs who meet regularly to contribute input on research conducted at OCHIN. In months 1-6, we will meet with the PEP monthly to engage them in a user-centered process on whether and how to redesign the CV Wizard 'Patient View'.

Data Collection

All outcome variables collected for analyses align with RE-AIM (Reach, Effectiveness, Adoption, Implementation, Maintenance), a widely used framework for evaluating implementation success. Below are details about the specific outcomes and measures for each. Please refer to Table 6 further below for additional information.

Primary Outcome Measures

- **Study Aim 1:** Compare patients' change in reversible risk in the Arm 1 vs. Arm 2 CHCs (in months 18-35)
- **Study Aim 2**: Assess whether the revised implementation support materials expedite CDS adoption
- **Study Aim 3:** Use mixed methods to identify multi-level barriers / facilitators to adoption of the CDS tool

Secondary Outcome Measures

- **Reach: Encounters affected** – % clinic encounters where CV Wizard suggests running the full risk assessment tool, i.e., identified a target patient.
- **Effectiveness (impact): Patient outcomes – 10-year pooled ASCVD risk score** – change in ASCVD risk score from baseline to last visit of study period (American College of Cardiology-ACC/ American Heart Association - AHA) 10-year pooled ASCVD risk score, 40-75 year olds
- **Effectiveness (impact): Patient outcomes - last BP ≤140/90** – Control of individual CVD risk factors: last BP ≤140/90
- **Effectiveness (impact): Patient outcomes - last A1c≤8** – Control of individual CVD risk factors: last A1c≤8
- **Effectiveness (impact): Patient outcomes – last LDL<100** – Control of individual CVD risk factors: last LDL<100
- **Effectiveness (impact): Patient outcomes - not current smoker** – Control of individual CVD risk factors: not current smoker
- **Effectiveness (impact): Patient outcomes - last BMI ≤25** – Control of individual CVD risk factors: last BMI≤25
- **Adoption: CDS uptake** – % of encounters where care team member opts to run the CV Wizard risk assessment (explored as both view and print rate and just print rate)

- **Implementation: User perceptions** – Perceived ease of use, usefulness, acceptability of CV Wizard; intent to use it.
- **Maintenance over time** – All measures over 2.5 years of follow-up, Arm 1; 1.5 years, Arm 2.

Table 6. Impact and uptake of the CV Wizard system: Quantitative Measures	
**Outcomes, per RE-AIM**	**Measurement**
**Reach:**<br>Encounters affected	% clinic encounters where CV Wizard suggests running the full risk assessment tool, *i.e.*, identified a target patient
**Effectiveness (impact):**<br>Patient outcomes	**(i) change in ASCVD risk score** (reversible risk; ACC/AHA 10-year pooled ASCVD risk score, 40-75 year olds; Framingham 30-year CVD risk score, 20-39 year olds).<br>**(ii) Control of individual CVD risk factors**: last BP ≤140/90; last A1c≤8; last LDL<100; not current smoker; last BMI≤25);
**Adoption**: CDS uptake	% of encounters where care team member opts to run the CV Wizard risk assessment
**Implementation:** User perceptions	Perceived ease of use, usefulness, acceptability of CV Wizard; intent to use it; see 3.3.f.ii
**Maintenance** over time	All measures over 2.5 years of follow-up, Arm 1; 1.5 years, Arm 2
**Potential covariates**	**Measurement**
Patient demographic characteristics	Age; gender; race / ethnicity; primary language; poverty level; insurance status at visit or insurance type through study period; # visits to that site / provider in last year; whether visit was with patient's primary care provider; provider type. Others TBD based on input from clinician advisors.
Other patient comorbid conditions	Renal function: diagnosed with end stage renal disease or chronic kidney disease; Charlson Comorbidity Score (Modified): Indicator of serious comorbid conditions that may shorten life expectancy, modified to exclude CV components; depression 296.xx, 311.xx diagnosis. Other relevant comorbidities TBD.
Visit type	New patient or established patient
Provider type	Provider type (PCP or other); # in patient panel
Other clinic factors	See Table 5; data from baseline survey
**CVD care / process**	**Measurement**
Recommended care that is ordered.	Whether care suggestions were acted on within 7 days of the encounter; *e.g.*, if CV Wizard suggests starting a statin, we will ask whether a prescription was issued.

b. **Study Aim 2 & 3**

Develop Training Materials

Research team members will work with OCHIN's training and QI teams, with CHC clinician input, to develop an initial set of resources for implementing CV Wizard in CHC workflows. These materials will be augmented over time, as described below, but at first will include information on how CV Wizard works, how it might be integrated into CHC workflows, and how care team members can use CV Wizard to present/discuss CVD risks with patients in a manner that minimizes patient discomfort, reduces potential adverse effects, and maximizes patient engagement.

Implementation Support

CV Wizard's impact will be affected by the CHCs' ability to implement it and achieve ongoing use at targeted visits. We will provide iterative, adaptive, pragmatic approach to providing the study CHCs with implementation support, as follows:

(1) When Arm 1 CHCs implement CV Wizard, they will receive the basic implementation

support that OCHIN's QI team provides with any new EHR functions (e.g., written materials, a live monthly webinar describing CV Wizard and suggested workflows for its use). We will also provide monthly CV Wizard use-rate reports to the Arm 1 CHC providers and clinic managers. CDS use rates are based on proportion of targeted visits where the CDS is viewed and / or printed.

(2) For the next 18 months, we will review monthly use rates of CV Wizard in the Arm 1 CHCs. The OCHIN practice facilitator will contact operational leaders at the CHCs with low adoption rates to ask about barriers to uptake, and what might help address these barriers; this aligns with the QI support that OCHIN regularly provides its member CHCs. We will work iteratively with OCHIN's Implementation and Training teams to develop and provide additional support as needed to address identified barriers to adoption and sustained use at the desired level (80% of targeted visits). The specifics of this ongoing support will be determined based on CHC need. However, based on our team's expertise/experience and the implementation science literature, we anticipate that some CHCs may need additional training on integrating CDS tools with clinic workflows; harnessing use rate data to guide subsequent efforts; the evidence-based guidelines underlying the CDS; and how to use CV Wizard to engage patients. Such trainings will be provided to clinic leaders, providers, managers, QI coordinators and administrators at the study CHCs via written materials and monthly webinars. If indicated, we will also provide academic detailing relating to the CDS, and customized implementation plans. These resources will become part of our revised implementation materials. We will also conduct monthly interactive webinars to answer questions, identify additional barriers to uptake/needed training, and enable peer-to-peer learning in the Arm 1 CHCs. If there are CHCs for whom these approaches do not enhance uptake, we will confer with CHC clinicians, OCHIN's QI team, and our team's implementation scientists to identify and implement alternative strategies.

(3) Arm 2 clinics will receive the revised implementation materials when CV Wizard is activated in their EHR. We will conduct a similar process to attempt to further improve these materials. We will collect quantitative data on which staff members from each study CHC take part in each training activity, and qualitative data on perceptions of the training strategies.

<u>Data Collection</u>

The mixed methods process evaluation will explore "what works, for whom and in what circumstances" related to CDS uptake, guided by our logic model and the Technology Acceptance Model (TAM). Our 3-tiered approach to data collection follows recent guidance on process evaluation of complex interventions which advocates complementing collecting key variables from all sites with in-depth data from purposively selected samples. This balances collecting data detailed enough to enable transferability (degree to which results can be applied to other contexts) and pragmatism (what is possible).

(1) <u>All Clinics</u> –We will collect the following information as it pertains to all clinics participating in the study: (1) baseline clinic data (e.g., location, patient characteristics, clinic characteristics, clinic size, etc.); (2) all 'trouble-tickets' relevant to CV Wizard submitted to OCHIN via its member help request system; (3) all user feedback submitted via CV Wizard's feedback mechanism; (4) all exchanges from the implementation

support webinars; and (5) feedback from the practice facilitator and others involved with implementation support.

We will also collect attendee evaluations of all webinars. This pragmatic, adaptive approach will let us explore how to help diverse CHCs implement CDS, fine-tune materials and strategies for doing so, identify key barriers to adoption of CDS, and inform developing a guide for CHCs implementing CV Wizard.

**SUPPLEMENT ADDENDUM**	
**Process outcomes**	**Description**
**Encounter patterns**	(i) Total clinical encounters; % in-person, by video, or by phone<br>(ii) % of patients whose encounters were *all* in-person, all VC, or a mix of both<br>(iii) % encounters where CV Wizard alerts that there are CDS suggestions, *i.e.*, identified a target patient
**Missed / delayed encounters**	(i) % of scheduled appointments that are missed / cancelled, by encounter type<br>(ii) Appointment wait times (time from scheduling call to appointment), by encounter type
**Tool use**	% of encounters where care team member 'used' (viewed) suggested CV Wizard CDS, by encounter type (among encounters occurring after CV Wizard was activated at the clinic)
**Primary outcomes**	**Description**
**CVD risk data up to date**	(i) % patients with up-to-date BP documented in EHR by 6 months post-index visit, and (ii) % patients with up-to-date A1c data documented in EHR by 6 months post-index visit, by encounter type<br>*Note:* These outcomes will be adjusted for # of encounters in follow-up period. Analyses of A1c updates will account for whether prior A1c measure indicated lack of control and need for more frequent monitoring.<br>*Note:* We will consider using coding of a hypertension diagnosis at index and follow-up encounters as a proxy for updated BP.
**Effectiveness** (all eligible patients)	*Needed care provided within 7 days of encounter*, by encounter type; e.g., If CV Wizard suggested (or would have suggested, if used) starting a statin, was prescription issued: YN; If an A1c test, was the test ordered: YN; If a referral to a nutritionist, was the referral made: YN; If medications to help quit smoking, was prescription issued; etc.
**Effectiveness** (subset of patients with an index encounter and ≥1 encounters ≥6 months post-index)	*Change in CVD risk over time,* measured as:<br>(i) Decrease in total reversible CVD risk score (ACC/AHA 10-year pooled ASCVD risk score[64-67])<br>(ii) Change from BP at index visit to increased, decreased, or no change, by end of follow-up period.<br>(iii) Among patients with *uncontrolled* A1c (>8%) at index encounter, % in control by end of follow-up period; among patients with *controlled* A1c (≤8%) at index, % who had a 'relapse' by end of follow-up.
**Potential covariates**	**Description**
Patient characteristics	Age; gender; race / ethnicity; poverty; insurance status at index; # encounters to site / provider, prior year
Comorbid conditions	Severe mental illness or diabetes diagnosis at index visit
Index characteristics	New patient to the clinic: YN

Post-index encounters	# in-person / VC encounters between index visit and end of follow-up period; was the encounter with the PCP: YN; visit's primary purpose; whether visit was for an urgent need: YN; patient new vs. established
Provider factors	Degree (MD, RN, PA, etc.); Prescribing privileges: YN; # in patient panel
**Independent vars.**	**Description**
Encounter type	In-person vs. VC telephone vs. VC video
CV Wizard use	CV Wizard 'used' (viewed / printed) when it alerted that CDS suggestions applied to a given patient: YN

(2) Selected Clinics

- We will conduct semi-structured phone interviews with care team members involved with implementing/using CV Wizard, about 10 months post-implementation. Follow-up interviews will be conducted as needed, to explore perceptions, acceptance, and use of CV Wizard and the implementation support provided. Sites will be purposively sampled for diversity in CV Wizard use rates.
- We will administer a provider survey that asks about use of the tool and solicits feedback. The survey will be programmed in REDCap and sent to clinic contacts via email. The clinic contact will be asked to email the providers at their clinics directly to request survey completion. Providers are informed (via the consent language that accompanies the survey) that (1) all responses/results are anonymous, and (2) we will aggregate non-identifiable response data to provide feedback to their clinic's study champion. NOTE: This survey may be administered to some clinics more than once over the course of the study period.

(3) Case Study/Supplement Clinics

- For case study sites, we will perform an in-depth ethnographic case study, which may include observation/personal interaction to explore the dynamics underlying implementation outcomes. We will follow the Arm 1 case study clinics for 2.5 years, the Arm 2 case study clinics for 1.5 years. Methods may include naturalistic observation (of workflows, patient encounters, clinic meetings), key informant and in-depth interviews, and collecting relevant artifacts (process maps, communications, etc.).
- For supplement sites, we will perform interviews with CHC staff and patients. Interviews will occur ≥3 months after CV Wizard is activated at a given clinic, and we will follow these clinics for one year.

7. Data Analysis

a. Analysis Plan

Overview

In this service area pragmatic clinical trial, we will implement CV Wizard via a staggered process. We will compare patients' CVD outcomes in the Arm 1 vs. Arm 2 CHCs, in months 18-35 (Aim 1); assess whether the revised implementation support materials expedite CDS adoption (Aim 2); and use mixed methods to identify multi-level barriers/facilitators to adoption of the CDS tool (Aim 3).

Quantitative Data

To be included in Aim 1 analyses a patient must have at least one visit in the 6 months after the Arm 1 implementation date at an Arm 1 or Arm 2 CHC and at least one additional visit at a study clinic in the 12-month post-index visit period. The first visit is referred to as the index visit. We will describe those who had inadequate follow-up data (did not have at least one additional visit in the post-index period). For each study subject, CVD risk score / risk factor management status will be calculated at index visit and each encounter during follow-up. While the tool can be run for patients of any age, we will restrict our analyses to patients 40-75 years of age.

We will test for between-group differences in baseline characteristics such as age, sex, race and ethnicity, insurance type, visit count, and health status using chi-square, t-test, and nonparametric Wilcoxon rank-sum tests as appropriate. We will also test for association between baseline characteristics and our outcomes of interest. We will examine the number of patients seen in each service area and clinic and each Arm and compare the characteristics of Arm 1 patients for whom the tool was used to those in Arm 1 for whom the tool was not used and to Arm 2 patients. Because the two arms were selected based on clinic size, percent of patients with a diagnosis for hypertension and the percent of patients that use tobacco, we anticipate there will be differences between the two groups resulting in the need to adjust for these differences. We will also examine characteristics at the service area and clinic-level in Arm 1 for whom the tool was used compared to Arm 1 service areas / clinics for whom the tool was not used and Arm 2 service areas / clinics.

Unadjusted trends in the monthly use and view rates for each of the study CHCs will be explored with use rates defined as: the number of encounters where CV Wizard was printed / number of targeted encounters with high-CVD risk patients per month and view rates defined as the number of encounters where CV Wizard was viewed / number of targeted encounters with high-CV risk patients per month.

We will also examine overall rates of each of the impact outcomes (BMI in normal range, controlled blood pressure, controlled LDL, controlled HbA1c, and non-smoking status) pre/post tool use to assess any changes in patterns for patients in Arm 1 for whom the tool was used compared to those in Arm 1 for whom the tool was not used as well as to Arm 2 patients. The date the tool was first used for each patient will be assigned as the start of the post-period.

Extent of adoption of a CDS tool like CV Wizard directly impacts its population-level impact. To differentiate between the tool's population level impact versus impact when used, we will conduct intent to treat (ITT) analyses to compare all targeted patients at intervention versus control clinics. We will also conduct effect of treatment on the treated (ETOT; also called per protocol) analyses limited to intervention CHC patients for whom the CV Wizard tool was used (results viewed / printed out) at the index visit, compared to intervention clinic patients for whom it was not used, and separately, to control clinic patients. The ITT analyses test the hypotheses that targeted patients in intervention clinics will have significantly lower CVD risk scores and higher rates of CVD risk factor control, at visits in the year post-index visit period, compared to similar patients in control clinics. The ETOT analyses test the hypotheses that targeted patients in intervention clinics for whom the CV Wizard CDS tool results are viewed and / or printed will have significantly lower CVD risk scores and higher rates of CVD risk factor control, at visits in the year post-index visit period, compared to similar patients in control clinics, and compared to patients in intervention clinics for whom the tool alerted but was ignored at the index visit.

Analyses will be stratified by first-time and repeat patients to see if there is a difference in tool use as well as examine whether the time between visits effects tool use among repeat patients.

Intent to Treat Analysis
Differences in change in outcomes will assessed using multi-level mixed models. Intra-class correlation coefficients will be obtained at the patient, clinic, and CHC organization levels for each outcome showed that much organization-level variance could be attributed to the clinic level. Thus, clustering was accounted for by random effects for clinic and patient, and a fixed effect for organization. Analyses will adjust for fixed effects: distribution of eligible patients by age, race / ethnicity, gender, rural / urban status, and federal poverty level (FPL) at index visit; number of ambulatory visits during the follow-up period; and time from index to last visit. These variables were selected *a priori* and will be confirmed by descriptive analyses, to determine whether there are significant differences between baseline intervention and control organization and patient characteristics. After checking distributions, negative binomial mixed-models or poisson mixed-model will be used for reversible risk analyses, and linear-mixed models for all other outcomes.

Patients diagnosed with diabetes during the follow-up period will be excluded from reversible risk analyses because diabetes might be diagnosed more often with CDSS use. Presence of diabetes raises reversible risk estimations even if CVD risk factor control was addressed; this exclusion will remove the possibility of confounding based on likelihood on new diabetes diagnosis.

Effect of Treatment on the Treated Analysis
Per-protocol analyses require adjusting for loss to follow-up and off-protocol therapies or treatments, but if group differences vary too greatly, model misspecification can yield biased estimates. Propensity score methods are an alternative. We will match intervention organization patients in three categories of tool use (never used during follow-up, used once, used more than once) to control organization patients. Counts of tool use will include use at index visit or subsequent study period visits, excluding the last visit. Matching variables will include age, FPL, outcome of interest at baseline, race / ethnicity, sex, count of ambulatory care visits post-index visit, time from index visit to last study period visit, and clinic rural / urban status. Propensity scores will be estimated using nearest-neighbor matching with replacement.

As analyses in other settings indicated CV Wizard's potential for greater impact among patients with higher baseline risk, analyses of change in reversible risk will be stratified by baseline risk <10% or ≥10%. The assumption being that patients with lower baseline risk are less likely to improve. Additionally, we will run analyses by quartile of baseline reversible risk among patients for whom the tool was ever used during follow-up, matched to controls as described above. Analyses of change in reversible risk will include all patients meeting study inclusion / exclusion criteria; for other outcomes, analyses were restricted to patients with uncontrolled baseline risk. Linear mixed models will be used for all outcomes and checked for normality of residuals.

Supplement Addendum
(1) Descriptive Analyses (Aim 2a)

- We will conduct a descriptive analysis on the following process outcomes: (1) Encounter patterns; (2) Rates of missed or cancelled appointments at in-person vs. VC (telephone, video) encounters; (3) Appointment wait times by encounter type, and (4) CV Wizard use (in encounters where there are CV Wizard CDS suggestions by encounter type.
- We will use standard statistical tests (e.g., chi-square, t-tests) to describe measures in the periods pre- vs. post-COVID's onset.

(2) Regression Analyses (Aim 2b)

- Encounter is the unit of analysis.
- We will assess associations between encounter type and: (1) Action taken on needed care plan components; (2) Up-to-date documentation of BP and A1c by 6 months post-index visit (accounting for whether BP data were taken in person or reported by the patient); and (3) Change in CVD risk factors by end of follow-up period. To do so, we will conduct adjusted regression analyses using longitudinal electronic health record data.
- To assess *action taken on needed care,* data for each factor will be extracted at each visit in the study period. We will use adjusted logistic regression models, with a dichotomous outcome of action taken or not taken.
- To assess *impact on individual CVD risk factors*, data for each factor will be extracted at index visit and all visits in the follow-up period (coded as controlled vs. not).
- Generalized linear mixed models (GLMM) with time nested within patients will be used to determine if risk factor control differs across encounter types, to estimate the effect of encounter type on change over time in CVD risk score and BP and A1c control. All encounters for each eligible patient will be included. CVD risk is influenced by factors amenable to clinical intervention (*e.g.*, BP) and others that are irreversible (age); absent intervention, overall risk should increase with age. These analyses will be adjusted for or stratified by potential confounders at the patient, provider, and encounter level. This will include accounting for whether post-index visit encounters were in-person or VC, and the reason for the encounter, as in-person encounters in the post-COVID period may be for different reasons than VC encounters.
- To assess whether CV Wizard moderated the relationship between encounter type and CVD outcomes, we will also conduct these analyses using only encounters where CV Wizard alerted the user that there are CDS suggestions. We will compare outcomes between encounters where CV Wizard was used (viewed or printed out) vs. not used, by including its use as a variable in the models described above.
  Data on race / ethnicity and gender will be obtained for all study patients; analyses will adjust or stratify for them as indicated.

Statistical Power

To conservatively calculate power for our main outcome (CVD risk score) we estimated 60 clinics with >35 high-CVD risk patients, randomized to two arms; then, using means and standard deviations for this measure from previous studies, we varied effect size associated with the group x time interaction and intraclass correlation. We examined a 1.5%, 2%, 3% greater absolute reduction in risk score over time in intervention vs. control CHCs. Aggressive management can reduce 10-year CVD risk by an absolute 4-5% in high-risk patients in a short time. We do not expect so large an effect and have power to detect smaller effects. **Supplement Addendum (Aim 2b).** This is a 2x2 repeated measures design with two groups of subjects measured at two time points. The primary goal is to compare change across time in group 1 (in-person index encounters) to change across time in group 2 (VC index encounters). A

conservative estimate (based on preliminary results from the parent study) of 6000 patients in group 1 and 6000 in group 2 achieve 100% power to detect a difference in mean changes in CVD risk score of 5.0, with a standard deviation of 9.1 at the first time point, a standard deviation of 9.1 at the second time point, and a correlation between measurement pairs of 0.200. The significance level (alpha) is 0.050 using a two-sided, two-sample t-test.

<u>Qualitative Data</u>
Using a realist approach our process evaluation will explore "what works, for whom and in what circumstances" related to uptake/impact of the CDS system. It will be guided by our logic model and the TAM's conceptualization of the relationship between user perception and CDS acceptance/use. We will identify mechanisms of change (barriers/facilitators; impact of implementation strategy) at the clinician and health care system levels and assess how contextual factors impact outcomes. **Supplement Addendum**: The parent study involves assessing how CV Wizard is used at in-person visits. We will account for the shift to VC by adding qualitative data on its use in VC. As in the parent study, process evaluation will be informed by the Technology Acceptance Model to understand (1) how CVD care takes place in VC and (2) the role of CDS in VC.

We will triangulate process data from all sources for a deep understanding of CV Wizard's acceptance/use in the CHCs. We will look at areas of consistency/inconsistency in these data (where different data sources show the same thing, or do not). A grounded theory approach coupled with an immersion-crystallization process will be used to identify themes and patterns in the qualitative data. We will emphasize factors influencing use of CV Wizard and its impact on care decisions. Data collection and analyses will be parallel and iterative, letting us identify salient constructs and knowledge gaps while implementation is ongoing. The study team will meet regularly to discuss and integrate qualitative and quantitative process evaluation and outcome data to help identify factors that affect success. The integration of mixed methods data will provide a more complete, nuanced understanding of the impact and use of the CV Wizard system than either method alone, and permit examination of the reliability and validity of the data sources. **Supplement Addendum:** Supplement data will be added to an existing NVIVO database to allow integration of findings. It will also be flagged to facilitate sub-analyses specific to this supplement's goals. To incorporate data from this study while allowing new themes to be considered, we will use an immersion-crystallization process which entails multiple iterations of data immersion, reflection, and code development and application, to identify new data themes or patterns related to the use of CV Wizard, and its impact on CVD risk management, in the VC context.

b. Sharing of Results with Subjects

If the CV Wizard decision support tool is used during a patient encounter, the provider has the option to print the provider-facing report. If the provider wishes to discuss the results with the patient, he/she may print the patient-facing report and give a copy to the patient or post to clinic's patient portal (if available). To ensure patient confidentiality (i.e., the correct patient receives the printout), the patient's name is printed on the top of the report.

c. Data and Specimen Banking

Not applicable.

8. Privacy, Confidentiality, and Data Security

Quantitative Data Management, Sharing and Protection
OCHIN uses safeguards similar to those used at CHR to ensure confidentiality when handling data. All patient-related study data will be de-identified and unique patient identification codes will be used, and all data sources will be linked through a secure relational database at OCHIN. Data sent to CV Wizard, and then returned to OCHIN for analysis, will be de-identified and protected as described in the Security Measures section below. Only aggregate data will be shared with CHR through a secure data transfer web site, as necessary. The CV Wizard CDS system is a web service that uses input EHR data to identify eligible patients, compute CV risks, prioritize CV risk reduction, and provide treatment recommendations based on a complex set of evidence-based algorithms. A web-based display of the results is then provided to the primary care providers.

Qualitative Data Management, Sharing and Protection
Qualitative data will be securely stored at both CHR and OCHIN. All data work will be done on password-protected workstations, in a secure environment (HIPAA-compliant). At CHR, only study team members have access to the saved data. At OCHIN, access to study data is limited to the research department.

Data will be catalogued within 1 week of collection and will be entered in QSR NVivo. Data sharing agreements/protections will be set as necessary, and electronic data transfers will use a secure website or encrypted email. Hard copies of fieldnotes will be stored in a locked office at KPCHR that only research staff can access.

Audio recordings of staff/patient interviews will be stored on a secure file server that is accessible via password protected computers. Audio recordings will be transcribed by a KP/CHR-approved vendor. Audio files and transcripts will be transferred between CHR and the transcriptionist using a secure file transfer website. All audio files will be destroyed at the end of the study.

When research staff travel to/from the case study site clinics, they will follow the CHR procedure for physically moving data. The data being transferred may include encrypted audio recordings and notebooks containing fieldnotes (e.g., observation date/time, provider/staff name, patient encounter activity, etc.). Because clinic visits may take place at various locations and times throughout the day, there may be some instances (e.g., early morning or late afternoon/evening) that necessitate study staff transporting study materials to their home. In these cases, staff will:

(1) Place all data in a secured pouch with the appropriate labeling outlined in the CHR procedure for physically moving data.
(2) Transport data in the trunk of their vehicle and commute directly home.
(3) Bring the data into their home and place in a secure location.
(4) Transport data directly to KPNW facility.

Storage of Recruitment Data
As outlined in Section 5.e (Case Study & Supplement Site Recruitment), we may identify patients for qualitative interviews using a BPA alert in the EHR (where providers flag patients) or by pulling data on smartphrase use in the after-visit summary (AVS) that indicates CV Wizard was used during an encounter. In both cases, patient data will need to be accessed and stored for recruitment and

tracking purposes. We will store the following data in OCHIN's instance of REDCap: patient name, encounter date, referring provider, clinic name, mailing address, phone number, email address, and notes entered by the study team related to recruitment efforts (e.g., outreach type, date of outreach, etc.). NOTE: Only OCHIN staff will have access to this information.

Additional Information about Sensitive Data
We plan to pull data related to mental health diagnoses (depression, anxiety, and severe mental illness) to determine whether it explains variance in CV Wizard tool use. This data will be pulled from the EHR (i.e., this information will *not* be collected directly from patients that are participating in the qualitative interviews). We will follow the same safeguards for this sensitive data as we do for all data collected in this study (see sections on data management, sharing, and protection; confidentiality; and security). This data will be collected (as applicable) for all clinics participating in the study. Given that individual patients are not enrolled for this aim of the study, there is no consent form or PRA to which this information should be added. NOTE: Each OCHIN member clinic has business use agreements with OCHIN to handle and manage PHI from their clinical data, and each CHC agrees that their electronic health record data may be used in research as part of their membership agreement.

Confidentiality Measures
All CHR investigators and project staff, all HealthPartners data managers, and all OCHIN staff who work on research projects and handle human subjects data/have human subjects contact, sign confidentiality pledges and receive IRB and HIPAA training/certification.

Security Measures
Multiple measures are in place to ensure the security of PHI. Data transfer to and from the EHR (at OCHIN), the web service (at Health Partners), and the web display (accessed at OCHIN member CHCs) uses a Simple Object Access Protocol (SOAP) with Secure Sockets Layer (SSL) encryption over a Hypertext Transfer Protocol Secure (HTTPS) computer network. There is a double firewall in the Web service so that once the data flow through the initial Web service firewall, the data cross another firewall into a new secure pathway that once again employs SOAP, SSL, and HTTPS to process the data and provide recommendations. This includes sending the data through a batch server for more efficient processing, but all within the double-firewall Web service. Limited clinical data for all adults are initially extracted from the EHR to determine CDS eligibility.

We estimate that 15-25% of study clinic patients may meet the eligibility criteria at which point more clinical information is extracted and full processing through the CV Wizard clinical algorithms occurs and is displayed back to the providers. If/when the CV Wizard tool is used, to ensure patient confidentiality, the name of the patient is printed on the CDS sheet given to the patient and their care provider at the clinic visit. This is needed to avoid mixing up printed pages and giving protected health information (PHI) to the wrong patient. It is also desirable to have each patient's name on this sheet of paper to assure the patient that the information on the paper is related to their own health state and not someone else's. The technical aspects of this system are also relevant; the printout is controlled from the CDS Web site, so the Web site must have the patient's name and other PHI.

With the analytic databases, measures will be taken to protect PCPs and patients from the risk of breach of confidentiality: A unique study ID code unrelated to the EHR record number or other study subject-specific information will be assigned to each patient and provider study subject and

used to link data from various sources needed for analysis. A crosswalk table linking this code number to a provider PCP or patient name or medical record number will be destroyed within 12 months of completion of the linked databases needed for study analyses. To minimize the risk that a PCP will act wrongly on the basis of information provided through CDS developed for this study, communication to providers will have a written explanation that the CDS is a suggestion, not a mandate, and that the action should only be taken if judged to be clinically appropriate by the treating provider on the basis of the patient's current clinical status and preferences.

9. Provisions to Monitor the Data to Ensure the Safety of Subjects

This study involves data from OCHIN, and through OCHIN, patient data from enrolled CHCs. The web service for CV Wizard© is hosted by Health Partners. All EHR data from OCHIN clinics are stored at OCHIN. Each clinic has business use agreements with OCHIN to handle and manage PHI from their clinical data. EHR data will be linked using OCHIN's unique patient identification codes, and data sources will be linked into a secure relational database at OCHIN. When data linkages have been completed, the data will be fully de-identified. Data analysis will be conducted at OCHIN. De-identified, aggregate data will be shared with study team members from CHR and HealthPartners.

Yearly datasets will be created with data from CV Wizard's web server, imported into SAS, and securely maintained at OCHIN. Data will be extracted for all target patients seen in OCHIN study CHCs and stored (limited data set) in a Web-based secure repository. Data from subsequent encounters is stored and linked to the same patient. If necessary, to share PHI, we will use a secure data transfer website with access limited to appropriate members of the research team. IRB and HIPAA approval will be obtained for all study steps. CHR and OCHIN have expertise in high quality data management and project operations and multisite research collaborations. Data structures are typically designed to separate personal identifiers from other critical data, further enhancing protections. CHR, HealthPartners, and OCHIN standards meet or exceed requirements for patient data safety established in the federal HIPAA guidelines. Data structures will derive from the data confidentiality, security, and privacy standards that CHR/OCHIN/HealthPartners have in place to meet or exceed all current HIPAA requirements. These standards exceed Level 1 requirements, and meet the majority of Level 2 requirements, as specified by DHHS in its Automated Information Systems Security Handbook. All analyses will be carried out using SAS® version 9 or later. Quality control will begin with real-time, inter-field checks in the data at OCHIN. Additional back-end checks (for missing data and logical inconsistencies) will be conducted to ensure the highest standards of data reliability. We will examine the distribution and measurement properties of variables before making final decisions about analyses.

In addition, we will compose a data safety and monitoring board (DSMB) of three members with expertise in clinical trials methodology and the clinical domains addressed in the proposed research. Members will include one clinician with expertise in CV disease and treatment, one expert in information technology, and one statistical expert. The PI will participate in the DSMB meetings in a limited way, as recommended in NIH policy. The DSMB will provide input and guidance on the study evaluation and intervention protocols, including quality assurance and safety issues related to the protocols and intervention strategy, as well as data-handling activities. The DSMB will provide periodic input via email, conference calls, and annual meetings. The DSMB will convene for one meeting in project year 2 to review the study protocol and adopt a formal charter. They will then convene twice each year during project years 3-4 (the active intervention phase) and one final time in project year 5 to review study results. A special focus of interest will be the safety of patients

exposed to the study intervention. The intervention provides point-of-care CDS related to management of elevated CV risk and suggests evidence-based treatment options based on national guidelines, and further vetted by clinical leaders at OCHIN and HealthPartners. CDS recommendations provided as part of the intervention are designed to support clinicians' decision-making, not to override clinical judgment. Information related to adverse events will be collected by OCHIN and/or HealthPartners and sent in writing to KPCHR for reporting to the IRB.

10. Risks and Benefits

a. Risks to Subjects

Breach of Privacy/Loss of Confidentiality

One risk to clinic staff and patients is breach of privacy and subsequent loss of confidentiality, but this is extremely unlikely. All CHR, HealthPartners, and OCHIN staff are highly trained and appropriately certified in data security and confidentiality, and the current protection measures represent the cutting edge of electronic protection. All patient data used in analyses will be de-identified by OCHIN staff, who work with patient data from their member clinics on a regular basis and are appropriately trained in data security. Thus, we feel any risk due to loss of confidentiality is remote.

CV Wizard Recommendations

Another potential risk is the possibility that the CV Wizard CDS tool may provide advice (according to national evidence-based CVD guidelines) that is inappropriate for a given individual patient and, if applied without further checking the clinical status of the patient, could lead to erroneous therapy or adverse events. However, the recommendations generated by CV Wizard are evidence-based and operationalize current national and regional standards of care and, therefore, the risk of untoward consequences of such clinical actions is considered minimal. Moreover, this potential risk is routinely present in every clinical encounter in the healthcare system. All treatment recommendations will be evidence-based, but acting on them will ultimately be up to the provider's judgement; thus, we do not anticipate any clinical harm to patients based on patients/their providers seeing the CV Wizard recommendations. We have described below the methods used to minimize this risk. In addition, our qualitative data collection will include a focus on potential harms to patients – such as anxiety – that may be incurred by their exposure to the CV Wizard CVD risk assessment.

b. Potential Benefits to Subjects

Overall Benefits

This study is expected to yield several key benefits. It will determine whether and how clinical decision support (CDS) tools that address multiple aspects of guideline-based CVD prevention and care, have provider- and patient-facing elements (enabling patient engagement), and involve some workflow changes, can and will be adopted by, and will successfully increase rates of guideline-concordant care in CHCs. The use of sophisticated CDS in populations with persistent disparities is a long overdue, critical step towards addressing CVD risk/outcome disparities in socioeconomically vulnerable (i.e., CHC) populations in the US. This study will determine how persistent guideline-to-

practice gaps impacting high-risk/prevalence patients can be addressed using targeted, innovative, multi-level, team-based decision support tools. Thus, key benefits include: (1) Collecting, documenting and presenting data on prioritized CVD risk to care teams and patients may result in patients receiving care that ultimately improves their health. This potential benefit will incur no additional clinic visits or costs to patients. (2) The intervention will increase care teams' knowledge about CVD risks that may influence their patients' health. (3) This intervention may bring improvements in EHR functionality to CHCs that would otherwise not receive them. (4) Because this study develops and tests different ways of presenting CVD risk data in clinic EMRs, it will provide needed information on how prioritized CVD risk data can be used to improve care and services in community health center populations nationwide.

Findings will support further improvements in the US healthcare system to mitigate health disparities and will inform future efforts to bring cutting-edge CDS to CHCs. If the intervention significantly improves primary care with respect to identification or management of elevated CV risk factors in adults, the risk of CVD events and/or mortality related to elevated CV risk may be reduced later in life for large numbers of patients. If the intervention fails to improve identification or management of CV risk factors, that knowledge will also be important because it will direct the attention of investigators to other potentially more fruitful lines of investigation. Thus, regardless of specific findings, the results of this trial will provide important new knowledge that may ultimately contribute to improved care for adults with elevated CV risk.

<u>Patients and Clinic Staff</u>
Patients at the study CHCs will have no defined personal benefit from this project. However, the CVD reduction recommendations may help assist in better patient-provider communication around CVD risks and lead to improved treatment outcomes for high-risk patients.

Clinic staff at the study CHCs will have no defined benefits from participating in this project. However, the intervention is designed to optimize identification and management of adult subjects with elevated CV risk. Some providers exposed to this potentially useful CDS may use it to improve their clinical care during the study or after.

11. Costs to Participants

Patients will not incur any costs if CV Wizard is used during their office visit. Clinic staff and patients that participate in the qualitative interviews will not incur any costs.

12. Compensation to Participants

Case study site clinics will receive annual impact fees to compensate for staff time spent on qualitative data collection efforts. Clinics will receive $1000 for each year they participate as a case study site. Supplement study clinics that participate in qualitative activities will receive an impact fee of $1610 for their year of participation.

Patients who participate in Aim 2 and 3 qualitative interviews (either by phone or in-person) will each receive a $25 gift card. Staff who participate in Aim 2 and 3 qualitative interviews (either by phone or in-person) may receive a $25 gift card if their workplace allows them to

accept research incentives. Each MOU will outline the approved form of compensation, if any.

Supplement Addendum: Patients who take part in the supplement qualitative interviews will each receive a $30 gift card. Clinic staff that participate in supplement interviews will not be individually compensated. Instead, the clinic will be compensated with a greater clinic impact fee. NOTE: This approach has been taken for these clinics, as many staff are unable to accept research incentives.

Gift cards for telephone/remote interviews will either be post mailed or emailed (depending on gift card type). Any personal information collected for the purpose of sending a gift card will be stored electronically on the study file service at CHR, accessible only to study staff members.

13. Resources Available

No special resources or expertise are required to conduct this study.

14. Drugs or Devices

Not applicable.

15. Multi-Site Coordination

CHR will act as the coordinating center for this study. We will ensure that:

- All sites have the most current version of the protocol, consent document, and HIPAA authorization.
- All required approvals have been obtained at each participating site (including approval by the site's IRB of record if required).
- All protocol modifications have been approved and communicated to sites (including approval by the site's IRB of record, if required) before the modification is implemented.
- All engaged participating sites will safeguard data as required by local information security policies.
- All local site investigators conduct the study appropriately.
- All non-compliance with the study protocol or applicable requirements will be reported in accordance with local policy.
- Communication of problems, interim results, and study closure.

16. Community-Based Participatory Research

OCHIN's leadership strongly supports activating CV Wizard in its member CHCs, which aligns with OCHIN's ongoing efforts to improve care and outcomes in CHCs via EHR-based strategies. In all study steps, the study team will engage OCHIN's operational leadership (including a CHC clinician and informaticist). We will also engage OCHIN CHC clinicians via existing communication structures. OCHIN has a long history of engaging stakeholders in all system-wide efforts; CHC clinicians serve on standing committees that direct all changes made to OCHIN's EHR. These committees include:

OCHIN’s Executive Leadership Team, the Practice-based Research Network (PBRN) formed in 2006, the Clinical Operations Group, and the Clinical Review Advisory Committee. For this project, we will engage OCHIN leaders and clinicians by working directly with these groups to obtain their input and direction at key junctures.
