# Supplementary material for: Effect of Clinical Decision Support at Community Health Centers on the Risk of Cardiovascular Disease: A Cluster Randomized Clinical Trial
Source: JAMA Netw Open. 2022 Feb 4;5(2):e2146519. doi: 10.1001/jamanetworkopen.2021.46519 (PMC8817199; doi:10.1001/jamanetworkopen.2021.46519)
Supplement: Supplement 2. — eFigure 1. CV Wizard Provider View (Prioritizes Patient Risks and Provides Decision Support Suggestions) eFigure 2. CV Wizard Patient View (Prioritizes Patient Risks and Presents the Information Visually) eMethods. Reversible Risk Calculations eTable. Clinic Characteristics by Study Group at Baseline eReferences [file jamanetwopen-e2146519-s002.pdf]

Supplementary Online Content

Gold R, Larson AE, Sperl-Hillen JM, et al. Effect of clinical decision support at community health centers on the risk of cardiovascular disease: a cluster randomized clinical trial. *JAMA Netw Open*. 2022;5(2):e2146519. doi:10.1001/jamanetworkopen.2021.46519

eFigure 1. CV Wizard Provider View (Prioritizes Patient Risks and Provides Decision Support Suggestions)
eFigure 2. CV Wizard Patient View (Prioritizes Patient Risks and Presents the Information Visually)
eMethods. Reversible Risk Calculations
eTable. Clinic Characteristics by Study Group at Baseline
eReferences

This supplementary material has been provided by the authors to give readers additional information about their work.

eFigure 1. CV Wizard Provider View (Prioritizes Patient Risks and Provides Decision Support Suggestions)

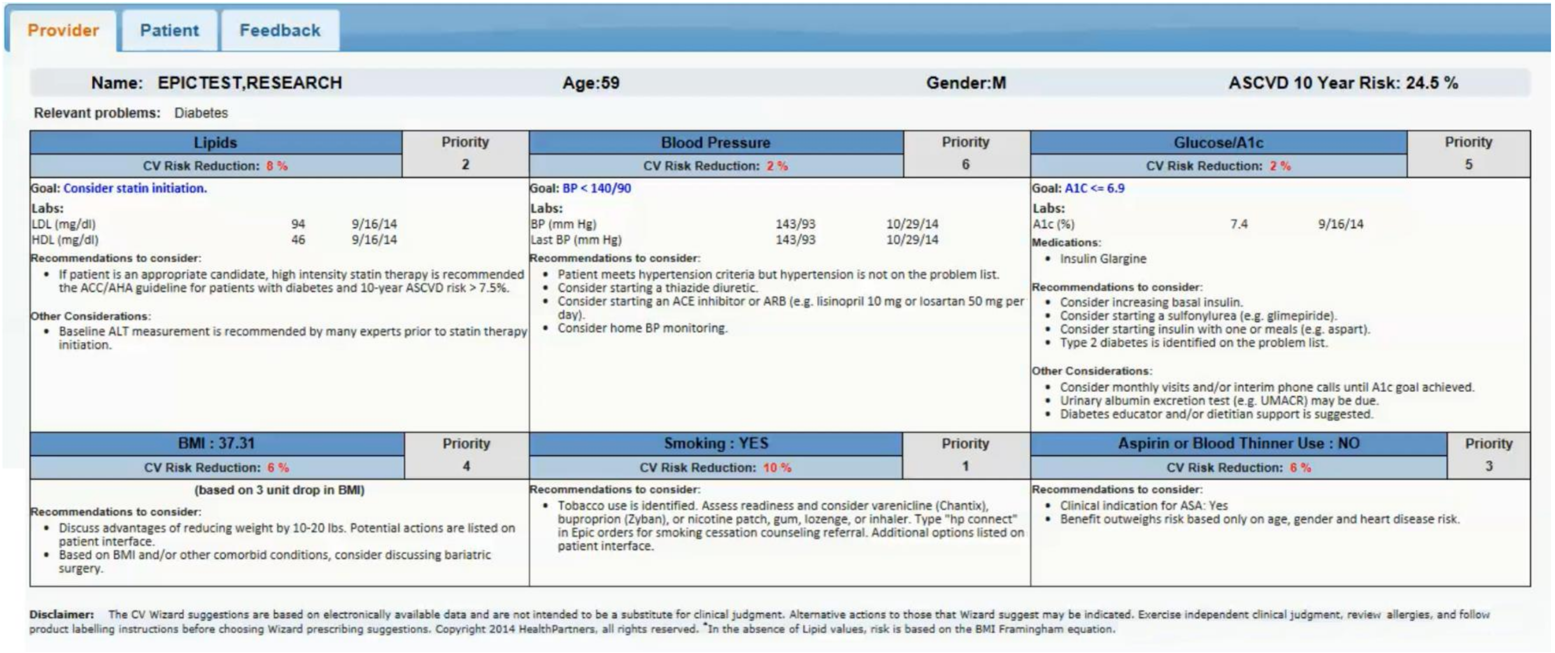

© 2022 Gold R et al. *JAMA Network Open*.

eFigure 2. CV Wizard Patient View (Prioritizes Patient Risks and Presents the Information Visually)

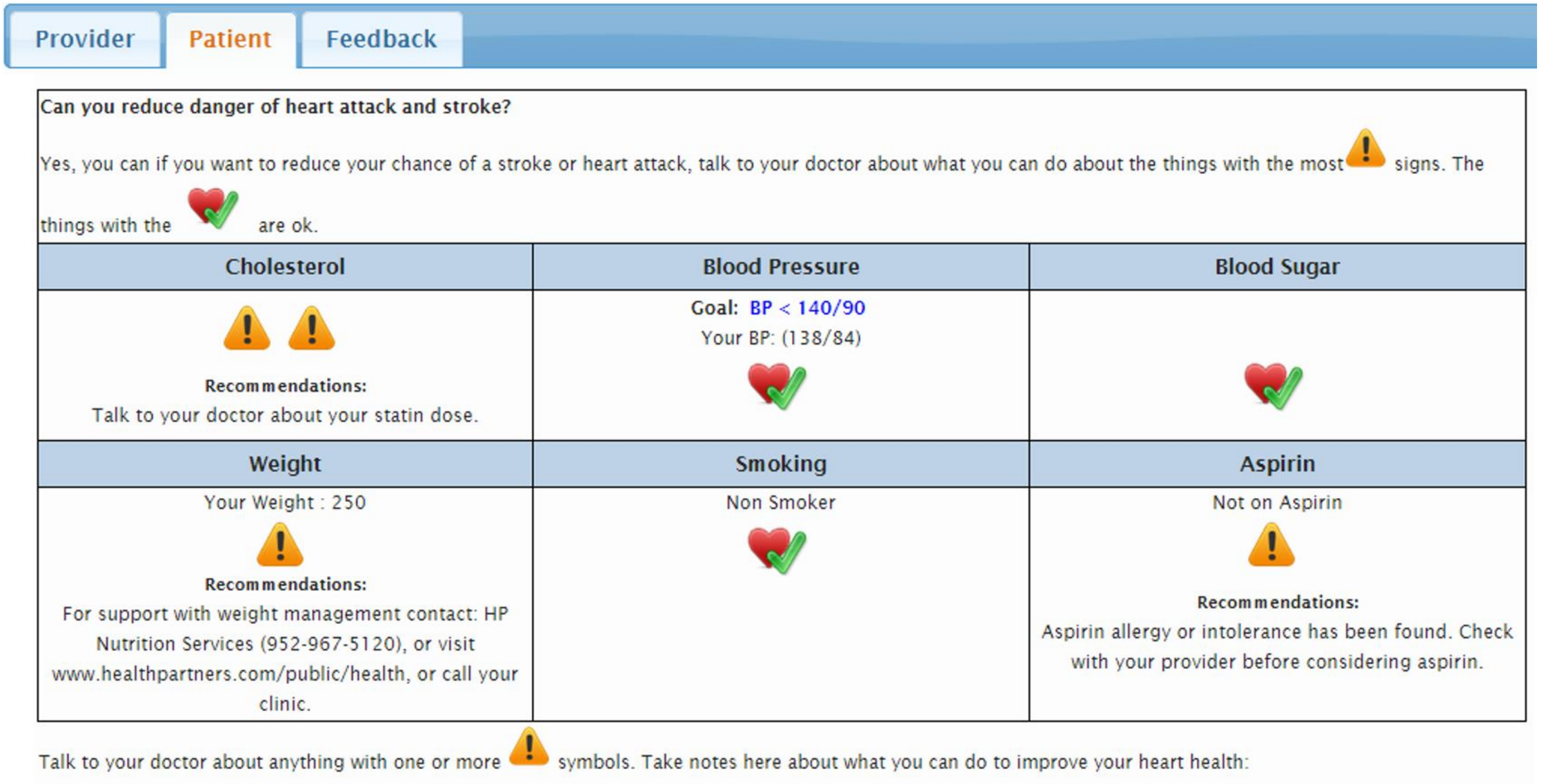

© 2022 Gold R et al. *JAMA Network Open*.

eMethods. Reversible Risk Calculations

Reversible CVD risk was calculated and used as follows. First, standardized risk equations were used to estimate the potential reduction in a given patient's age, sex, and race-specific CVD risk for up to six specific uncontrolled CVD risk factors: (1) SBP ≥140 mmHg, (2) A1c≥8%, (3) not on guideline-recommended dose of statin, (4) not on aspirin or equivalent if guideline recommended, (5) current smoker, or (6) body mass index (BMI, Kg/m2) ≥25.

The standardized risk equations were used to estimate the reduction in CVD risk that would be achieved for each uncontrolled CVD risk factor if it were to reach the following specified thresholds:[1-4] (a) decrease in CVD risk based on ACC/AHA 10-year CVD equations if SBP changed from an observed SBP≥140 mmHg to a hypothetical SBP of 139 mmHg; (b) decrease in CVD risk based on ACC/AHA 10-year CVD equation if Total Cholesterol and HDL-cholesterol (HDL) changed from observed values to what they would likely be with statin initiation or increase to guideline-recommended statin dose; (c) decrease in CVD risk based on ACC/AHA 10-year CVD equations if patient changed from current to non-smoking status; (d) decrease in CVD risk based on Framingham BMI risk equations with a hypothetical decrease of 3 BMI units down to a minimum possible BMI of 25; (e) decrease in CVD risk based on United Kingdom Prospective Diabetes Study (UKPDS) Outcomes Model 2 equations for a decrease in A1c≥8% to a hypothetical A1c of 7.9%; and (f) estimated CVD risk reduction based use of on ACC/AHA 10-year CVD equation as suggested by the United States Preventive Services Task Force guidelines on appropriate aspirin use that were in effect during the study period.

The estimated CVD reversible risks for each uncontrolled CVD risk factor were used in three ways. First, the risk-factor-specific reversible CVD risk was used to rank each uncontrolled CVD risk factor from high to low priority based on potential risk-factor-specific CVD risk reduction for each patient. Second, the risk-factor-specific reversible CVD estimates were summed (up to 6 uncontrolled CVD risk factors) to provide an estimate of reversible CVD risk at a given time point. In analyses related to reversible CVD risk, change in reversible CVD risk was calculated by subtracting the reversible risk at a defined follow-up point from the reversible CVD risk estimate at the index visit, such that negative values represent favorable changes in reversible CVD risk.

This method of estimated reversible CVD risk has been described previously and used in prior studies. One benefit of focusing clinical attention on patients with high reversible CVD risk, rather than all patients with high total CVD risk, is that some patients with high total CVD risk (e.g., older males with no controlled CVD risk factors) may have very low reversible CVD risk and would not derive much benefit from discussing their high but irreversible CVD risk. Using this approach to reversible CVD risk also includes risk factors not adequately addressed by the main ACC/AHA CVD risk equations, namely change in A1c, change in BMI, and aspirin use.

This approach also has several acknowledged limitations. For example, it assumes that the benefit of becoming a non-smoker, or of better BP or lipid control, are applied immediately, whereas it may take longer for these benefits to occur. However, prior work suggests that changes in BP control, lipid control, and aspirin use may affect mortality within one year for many patients.[5-7] With respect to using this method to rank uncontrolled CVD risk factors for clinical attention, although estimation of reversible CVD risk at the patient level is imprecise, it is likely superior to the demonstrably erroneous estimates of CVD benefits and risks that now prevail in primary care. Further, the priorities presented for consideration may focus discussion on evidence-based clinical options of potential benefit and avoid spending valuable time discussing non-evidence-based options. Thus, prioritization methods that estimate reversible CVD risk using standardized risk equations may be imperfect, but still superior in most cases to less-informed, intuitive estimates of benefit or risk made by providers or by patients.

© 2022 Gold R et al. *JAMA Network Open*.

eTable. Clinic Characteristics by Study Group at Baseline

	**Intervention N = 42 (% / SD)**	**Control N = 28 (% / SD)**
FQHC	30 (71)	28 (100)
Location		
Urban	35 (83.3)	15 (52.6)
Suburban	2 (4.8)	1 (3.6)
Rural	5 (11.9)	12 (42.9)
Avg Years on Current EHR	8.7 (3.1)	7.2 (3.0)
Provider type		
Primary Care	622 (43.6)	444 (48.6)
Mental/Behavioral Health	71 (5.0)	28 (3.1)
Visit Type		
Primary Care	519,953 (77.5)	365,770 (78.3)
Mental/Behavioral Health	4,663 (0.7)	1,440 (0.3)
Ratio new: established patients	1:13	1:14
Total encounters during study period	671,260	488,193

© 2022 Gold R et al. *JAMA Network Open*.

eReferences

1. Goff DC, Jr., Lloyd-Jones DM, Bennett G, et al. 2013 ACC/AHA guideline on the assessment of cardiovascular risk: a report of the American College of Cardiology/American Heart Association Task Force on practice guidelines. *J Am Coll Cardiol.* 2014;63(25 Pt B):2935-2959.
2. D'Agostino RB, Vasan RS, Pencina MJ, et al. General cardiovascular risk profile for use in primary care: the Framingham Heart Study. *Circulation.* 2008;117(6):743-753.
3. Pencina MJ, D'Agostino RB, Sr., Larson MG, Massaro JM, Vasan RS. Predicting the 30-year risk of cardiovascular disease: the Framingham Heart Study. *Circulation.* 2009;119(24):3078-3084.
4. U.S. Preventive Services Task Force (2016). Aspirin use for the primary prevention of cardiovascular disease and colorectal cancer: recommendations from the U.S. Preventive Services Task Force. *Ann Intern Med.* 2016;164(12).
5. Ho PM, Spertus JA, Masoudi FA et al. Impact of medication discontinuation on mortality after myocardial infarction. *Arch Intern Med* 2006;1661842- 1847.
6. O’Connor PJ. Improving medication adherence: challenges for physicians, payers, and policy makers. *Arch Intern Med*. 2006 Sept 25;166(17):1802-4. PMID: 17000934.
7. O’Connor PJ, Vazquez-Benitez G, Schmittdiel JA, Parker ED, Trower NK, Desai JR, Margolis KL, Magid DL. Benefits of early hypertension control on cardiovascular outcomes in patients with diabetes. *Diabetes Care*. 2013 Feb;36(2):322-7. PMCID: PMC3554277.

© 2022 Gold R et al. *JAMA Network Open*.
