# Supplementary material for: Effect of Clinical Decision Support at Community Health Centers on the Risk of Cardiovascular Disease: A Cluster Randomized Clinical Trial
Source: JAMA Netw Open. 2022 Feb 4;5(2):e2146519. doi: 10.1001/jamanetworkopen.2021.46519 (PMC8817199; doi:10.1001/jamanetworkopen.2021.46519)
Supplement: Supplement 3. — Data Sharing Statement [file jamanetwopen-e2146519-s003.pdf]

Data Sharing Statement

Gold. Effect of Clinical Decision Support at Community Health Centers on the Risk of Cardiovascular Disease. *JAMA Netw Open*. Published February 04, 2022. doi:10.1001/jamanetworkopen.2021.46519

Data
Data available: No

Additional Information
Explanation for why data not available: The findings presented in this manuscript are based on electronic health record (EHR) data from patients at OCHIN clinics participating in the CV Wizard research study. All EHR data are proprietary to the OCHIN clinics and thus will not be made directly available beyond the study team.
